# Long‐term efficacy and safety of biosimilar infliximab (CT‐P13) after switching from originator infliximab: open‐label extension of the NOR‐SWITCH trial

**DOI:** 10.1111/joim.12880

**Published:** 2019-04-12

**Authors:** G. L. Goll, K. K. Jørgensen, J. Sexton, I. C. Olsen, N. Bolstad, E. A. Haavardsholm, K. E. A. Lundin, K. S. Tveit, M. Lorentzen, I. P. Berset, B. T. S. Fevang, S. Kalstad, K. Ryggen, D. J. Warren, R. A. Klaasen, Ø. Asak, S. Baigh, I. M. Blomgren, Ø. Brenna, T. J. Bruun, K. Dvergsnes, S. O. Frigstad, I. M. Hansen, I. S. H. Hatten, G. Huppertz‐Hauss, M. Henriksen, S. S. Hoie, J. Krogh, I. P. Midtgard, P. Mielnik, B. Moum, G. Noraberg, A. Poyan, U. Prestegård, H. U. Rashid, E. K. Strand, K. Skjetne, K. A. Seeberg, R. Torp, C. M. Ystrøm, C. Vold, C. C. Zettel, K. Waksvik, B. Gulbrandsen, J. Hagfors, C. Mørk, J. Jahnsen, T. K. Kvien

**Affiliations:** ^1^ Department of Rheumatology Diakonhjemmet Hospital Oslo Norway; ^2^ Department of Gastroenterology Akershus University Hospital Lørenskog Norway; ^3^ Research Support Services CTU Oslo University Hospital Oslo Norway; ^4^ Department of Medical Biochemistry Oslo University Hospital Radiumhospitalet Oslo Norway; ^5^ Institute of Clinical Medicine University of Oslo Oslo Norway; ^6^ Department of Gastroenterology Oslo University Hospital Rikshospitalet Oslo Norway; ^7^ K.G. Jebsen Coeliac Disease Research Centre University of Oslo Oslo Norway; ^8^ Department of Dermatology Haukeland University Hospital Bergen Norway; ^9^ Department of Dermatology Oslo University Hospital Rikshospitalet Oslo Norway; ^10^ Department of Gastroenterology Ålesund Hospital Ålesund Norway; ^11^ Department of Rheumatology Haukeland University Hospital Bergen Norway; ^12^ Department of Rheumatology University Hospital of Northern Norway Tromsø Norway; ^13^ Department of Dermatology Sankt Olav's Hospital Trondheim Norway; ^14^ Department of Gastroenterology Gjøvik Hospital Gjøvik Norway; ^15^ Department of Dermatology Haugesund Hospital Haugesund Norway; ^16^ Department of Gastroenterology Haugesund Hospital Haugesund Norway; ^17^ Department of Gastroenterology Sankt Olav's Hospital Trondheim Norway; ^18^ Department of Gastroenterology Sørlandet Hospital Kristiansand Norway; ^19^ Department of Gastroenterology Bærum Hospital Bærum Norway; ^20^ Department of Rheumatology Helgelandssykehuset Mo I Rana Norway; ^21^ Department of Dermatology Førde Hospital Førde Norway; ^22^ Department of Gastroenterology Telemark Hospital Skien Norway; ^23^ Department of Gastroenterology Østfold Hospital Fredrikstad Norway; ^24^ Department of Rheumatology Sørlandet Hospital Kristiansand Norway; ^25^ Department of Rheumatology Levanger Hospital Levanger Norway; ^26^ Department of Rheumatology Bodø Hospital Bodø Norway; ^27^ Department of Rheumatology Førde Hospital Førde Norway; ^28^ Department of Gastroenterology Oslo University Hospital Ullevål Oslo Norway; ^29^ Department of Gastroenterology Sørlandet Hospital Arendal Norway; ^30^ Department of Rheumatology Kongsvinger Hospital Kongsvinger Norway; ^31^ Department of Gastroenterology Lillehammer Hospital Lillehammer Norway; ^32^ Department of Rheumatology Østfold Hospital Moss Norway; ^33^ Department of Rheumatology Revmatismesykehuset Lillehammer Norway; ^34^ Department of Gastroenterology Vestfold Hospital Tønsberg Norway; ^35^ Department of Gastroenterology Hamar Hospital Hamar Norway; ^36^ Department of Gastroenterology Elverum Hospital Elverum Norway; ^37^ Department of Gastroenterology Bodø Hospital Bodø Norway; ^38^ Department of Rheumatology Betanien Hospital Skien Norway; ^39^ Patient representative Norges Psoriasis‐ og eksemforbund Trondheim Norway; ^40^ Patient representative Landsforeningen for fordøyelsessykdommer Oslo Norway; ^41^ Patient representative Norsk Revmatikerforbund Oslo Norway; ^42^ Institute of Cancer Research and Molecular Medicine Faculty of Medicine Norwegian University of Science and Technology Trondheim Norway

**Keywords:** biosimilar, chronic inflammatory disease, drug costs, health economics, infliximab, switching

## Abstract

**Background and objectives:**

The 52‐week, randomized, double‐blind, noninferiority, government‐funded NOR‐SWITCH trial demonstrated that switching from infliximab originator to less expensive biosimilar CT‐P13 was not inferior to continued treatment with infliximab originator. The NOR‐SWITCH extension trial aimed to assess efficacy, safety and immunogenicity in patients on CT‐P13 throughout the 78‐week study period (maintenance group) versus patients switched to CT‐P13 at week 52 (switch group). The primary outcome was disease worsening during follow‐up based on disease‐specific composite measures.

**Methods:**

Patients were recruited from 24 Norwegian hospitals, 380 of 438 patients who completed the main study: 197 in the maintenance group and 183 in the switch group. In the full analysis set, 127 (33%) had Crohn's disease, 80 (21%) ulcerative colitis, 67 (18%) spondyloarthritis, 55 (15%) rheumatoid arthritis, 20 (5%) psoriatic arthritis and 31 (8%) chronic plaque psoriasis.

**Results:**

Baseline characteristics were similar in the two groups at the time of switching (week 52). Disease worsening occurred in 32 (16.8%) patients in the maintenance group vs. 20 (11.6%) in the switch group (per‐protocol set). Adjusted risk difference was 5.9% (95% CI −1.1 to 12.9). Frequency of adverse events, anti‐drug antibodies, changes in generic disease variables and disease‐specific composite measures were comparable between arms. The study was inadequately powered to detect noninferiority within individual diseases.

**Conclusion:**

The NOR‐SWITCH extension showed no difference in safety and efficacy between patients who maintained CT‐P13 and patients who switched from originator infliximab to CT‐P13, supporting that switching from originator infliximab to CT‐P13 is safe and efficacious.

## Graphical abstract

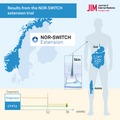

http://onlinelibrary.wiley.com/doi/10.1111/joim.12896/full

**Abbreviations**
ADAb: anti‐drug antibodies
AE: adverse event
ASDAS: Ankylosing Spondylitis Disease Activity Score
BASDAI: Bath Ankylosing Spondylitis Disease Activity Index
CDAI: Clinical Disease Activity Index
CD: Crohn's disease
DAS28: Disease Activity Score in 28 joints
DLQI: Dermatology Life Quality Index
FAS: full analysis set
HBI: Harvey–Bradshaw index
IBD: inflammatory bowel diseases
IBDQ: Inflammatory Bowel Disease Questionnaire
MHAQ: Modified Health Assessment Questionnaire
PASI: Psoriasis Area and Severity Index
PMS: Partial Mayo Score
PPS: per‐protocol set
PROM: patient‐reported outcome measure
PsAID: Psoriatic Arthritis Impact of Disease
PsA: psoriatic arthritis
Ps: plaque psoriasis
RAID: Rheumatoid Arthritis Impact of Disease
RA: rheumatoid arthritis
SDAI: Simplified Disease Activity Index
SpA: spondyloarthritis
UC: ulcerative colitis

## Introduction

Biologic agents such as the TNF‐α inhibitor infliximab have had a substantial, positive impact on the treatment of many chronic immune‐mediated inflammatory disorders, including inflammatory bowel diseases (IBD), inflammatory rheumatic diseases and chronic plaque psoriasis 1. However, access to these biologic treatments varies globally, and drug availability is limited in many countries due to the drug costs 2. Biosimilars are reproductions of their originator counterparts, are usually less expensive and therefore provide a potential opportunity to improve patient access. An increasing number of biosimilars are coming into clinical use 3, and so far, no clinical studies have shown any unexpected effects of starting patients on a biosimilar instead of an originator drug 4, 5, 6, 7, 8.

However, to achieve a significant impact on health budgets, it is not sufficient that only new patients starting biologic therapy are prescribed biosimilars. For this, it is also necessary that patients on current originator treatment switch to biosimilar agents. Since biosimilars cannot be exact copies of the original molecule, there are lingering concerns about switching stable patients to a biosimilar when they are doing well on an originator. So far, only a few randomized studies have examined the impact of switching patients from stable originator treatment to a biosimilar 9, 10, 11, 12, 13. The NOR‐SWITCH study was the first randomized controlled trial examining switching from an originator product to a biosimilar in stable, long‐term infliximab‐treated patients 14. Efficacy and safety were assessed in patients randomized (1 : 1) to switch to CT‐P13 or continue treatment with infliximab originator for a study period of 52 weeks 14. All six relevant diagnoses were included. The primary outcome measure was occurrence of disease worsening, with a number of disease‐specific secondary outcome measures. Serum drug levels of infliximab, as well as development of anti‐drug antibodies (ADAb), were assessed in every patient at each study visit. The NOR‐SWITCH study showed that switching from originator to biosimilar infliximab was not inferior to continued treatment with the originator and supported switching for nonmedical reasons, that is to reduce costs 14.

Very few randomized, industry‐independent studies on the effects of switching exist. Here, we present results from the 26‐week extension of the NOR‐SWITCH study, where all patients received open‐label treatment with CT‐P13. The efficacy, safety and immunogenicity of switching to CT‐P13 in patients treated with infliximab originator in the main study were compared to patients on continuous CT‐P13 treatment, providing a different comparator group to that of the main study. Also, the NOR‐SWITCH extension adds valuable, independent data on the longer‐term efficacy and safety of biosimilar CT‐P13.

## Methods

### Setting and participants

Patient recruitment and the inclusion and exclusion criteria for the NOR‐SWITCH study have previously been described in detail 14. Patients with a diagnosis of Crohn's disease (CD), ulcerative colitis (UC), spondyloarthritis (SpA), rheumatoid arthritis (RA), psoriatic arthritis (PsA) or plaque psoriasis (Ps) who had completed the main study period (week 0 to week 52) were recruited to a further 26‐week follow‐up period. All patients received verbal and written information about the extension study and signed an informed consent. After completion of the main study period, patients continued with open‐label CT‐P13 treatment at 34 participating centres (17 gastroenterology, 12 rheumatology and five dermatology hospital departments).

### Study design

The NOR‐SWITCH main study was designed as a 52‐week randomized, double‐blind, parallel‐group, multicentre, noninferiority comparative phase IV study. Full details on randomization, masking and statistical analyses have been published (Data S1) 14. The 26‐week extension study recruited 380 consenting patients who were all given open‐label CT‐P13 with an unchanged treatment regimen regarding dose and infusion intervals. Treatment given during the main study period remained blinded for all participants until 17 October 2016. On this date, information on main study intervention was made available to study personnel, who then passed this information on to patients at the discretion of the treating physician. At this point, there were only 68 participants (33 in the maintenance group, 35 in the switch group) who had still not completed their last visit in the extension study.

The study was conducted in compliance with the Declaration of Helsinki and the International Conference on Harmonization Guidelines for Good Clinical Practice. The study protocol and patient consent documents were approved by an independent ethics committee (Regional Committees for Medical and Health Research Ethics South East; reference number 2014/848), by appropriate institutional review boards and by the Norwegian Medicines Agency (reference number 14/07192‐11).

A project group including representatives from all three relevant specialities and with patient representatives from all three relevant patient organizations planned and conducted the study.

### Study assessments

Data were collected at every infusion visit using an electronic case report form similar to the main study (Viedoc^®^). Clinical assessment was performed by a trained study nurse and/or physician. Blood samples for protocol‐specified laboratory tests were obtained before infusions, including measurement of trough drug concentrations and ADAb, and for storage in a biobank. The patients with IBD were asked to deliver a faecal sample for calprotectin measurements after each visit (CalproLab; Calpro AS, Oslo, Norway, Buhlmann Laboratories AG, Basel, Switzerland). Full details about the collected variables can be found in the main study report 14. The main composite measures for the six diseases were Harvey–Bradshaw index (HBI) for CD, Partial Mayo Score (PMS) for UC, Ankylosing Spondylitis Disease Activity Index (BASDAI) for SpA, Disease Activity Score in 28 joints (DAS28) for RA and PsA, and Psoriasis Area and Severity Index (PASI) for Ps 14. The number of study visits differed according to treatment regimen.

### End‐points

The primary end‐point was disease worsening during the 26‐week follow‐up, defined by worsening in disease‐specific composite measures and/or a consensus on disease worsening between investigator and patient leading to major change in treatment (the same as in the main study) 14. Disease worsening according to disease‐specific composite measures was defined as ∆HBI ≥4 and HBI level ≥7 points for CD patients, ∆PMS >3 and PMS level ≥5 for UC patients, ∆ASDAS ≥1.1 and ASDAS level >2.1 for SpA patients, ∆DAS28 of ≥1.2 and DAS28 level >3.2 for RA and PsA patients, and ∆PASI ≥3 and PASI level ≥5 for Ps patients. ∆ indicates change from time of switching (baseline week 52) to the end of the 26‐week follow‐up period (study end minus baseline).

The secondary end‐points (for details, see the main study report 14) included changes in investigator and patient global assessments (study end (week 78) minus baseline extension study (week 52)), changes in erythrocyte sedimentation rate (ESR) as well as C‐reactive protein (CRP), overall remission status based on the main composite measures, and study drug discontinuation. Prespecified secondary end‐points for CD and UC were change and remission status of HBI and PMS, respectively, as well as changes in faecal calprotectin levels. In SpA, change in ASDAS and achievement of ASDAS inactive disease were prespecified. Secondary end‐points for RA and PsA included achievement of remission according to DAS28, Clinical Disease Activity Index (CDAI) and Simplified Disease Activity Index (SDAI) as well as ACR/EULAR remission criteria. Other secondary end‐points were changes in DAS28, CDAI and SDAI. In Ps, we prespecified the following as secondary end‐points: PASI complete clearance, mild‐to‐moderate disease, remission and change in PASI score.

Secondary end‐points also included patient‐reported outcome measures (PROMs): SF‐36 (change in each of the eight domains as well as the physical and mental component scores), ∆EQ‐5D and WPAI (change in absenteeism, presenteeism, work productivity loss and activity impairment). Changes in disease‐specific PROMs were also secondary end‐points (CD/UC: Inflammatory Bowel Disease Questionnaire (IBDQ); SpA/RA/PsA: Modified Health Assessment Questionnaire (MHAQ) score; SpA: Bath Ankylosing Spondylitis Disease Activity Index (BASDAI); RA: Rheumatoid Arthritis Impact of Disease (RAID) score; PsA: Psoriatic Arthritis Impact of Disease (PsAID) score; Ps: Dermatology Life Quality Index (DLQI)).

Measures of safety included clinical and laboratory adverse events (AEs). Coding of AEs was performed according to the Medical Dictionary for Regulatory Activities (meddra, v.13.0). Safety measures further included vital signs, laboratory data, infusion reactions, drug concentrations (trough measurements) and ADAb assessments.

INX/CT‐P13 trough serum levels and ADAb (for INX and CT‐P13) were analysed using in‐house target‐based assays automated on the AutoDELFIA^®^ (PerkinElmer, Waltham, MA) immunoassay platform 14. ADAb were not analysed in samples with INX/CT‐P13 levels above 5 mg L^−1^, since high drug concentrations cause interference in the assays for ADAb.

### Statistical analysis

The end‐points of this trial were primarily addressed in the per‐protocol set (PPS) and secondarily in the full analysis set (FAS). The PPS population consisted of subjects without major protocol deviations throughout the extension study, whilst the FAS population comprised all subjects entering the extension study.

Analysis of the primary end‐point (as well as other binary outcomes) was performed using logistic regression, with intervention group (switch versus maintenance) as a covariate. Continuous outcomes were analysed using linear mixed‐effect models, treating time as a categorical variable and including a time‐by‐treatment group interaction. Severely skewed variables, such as CRP, were log‐transformed prior to model estimation. Analyses of both binary and continuous outcomes were adjusted for diagnosis and duration of infliximab use at the time of switch (baseline extension study, week 52).

Missing data for remission and disease worsening in the FAS (*n* = 2) were imputed with last observation carried forward. For the primary end‐point, we applied the same noninferiority margin as in the main study (−15%) as a reference for the assessment of similarity between the groups.

### Role of the funding source

The funder of the study (Norwegian Ministry of Health and Care Services) had no role in study design, data collection, data analysis, data interpretation, writing of this article or decision to submit. All authors reviewed and approved the final manuscript. The corresponding author (GLG) together with the statistician (JS) had full access to all the data in the study and had final responsibility for the decision to submit for publication.

## Results

**Figure 1:**
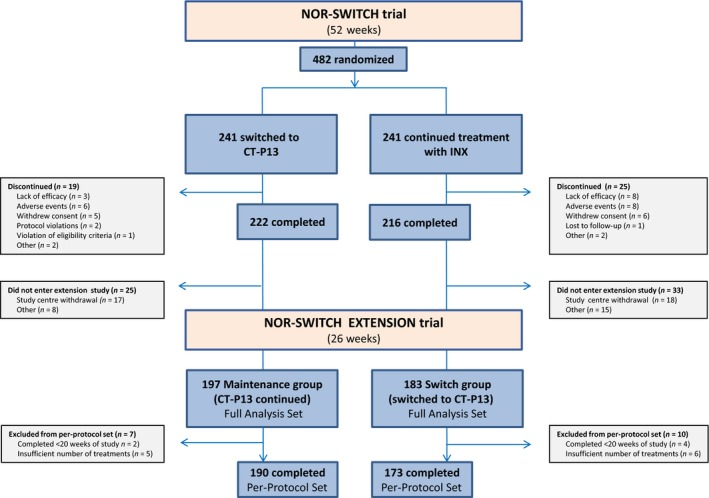
Patient disposition in the NOR‐SWITCH main and extension study.

Between October 2014 and July 2016, 482 patients were enrolled and randomized into the NOR‐SWITCH main study at 40 study centres. Based on the randomization, 241 patients continued treatment with infliximab originator and 241 switched treatment from infliximab originator to CT‐P13. Between 19 October 2015 and 15 June 2016, 380 of the 438 patients who completed the 52‐week main trial continued into the 26‐week extension study (Fig. 1). Six of the 40 former study centres did not recruit patients to the extension phase, this being the main reason for patient discontinuation (Fig. 1). The patients treated with CT‐P13 in the main trial continued this treatment in the extension phase (maintenance group) whereas patients treated with originator infliximab in the main trial switched to CT‐P13 at extension study baseline at week 52 (switch group). Consequently, the maintenance group consisted of patients treated with CT‐P13 for 18 months and the switch group received CT‐P13 for 26 weeks during the extension part of the study.

FAS included 197 patients in the maintenance group and 183 patients in the switch group, and PPS consisted of 190 and 173 patients in the maintenance group and the switch group, respectively (Fig. 1).

The mean age at inclusion in the extension phase was 48.4 years (SD 14.9), and 142 (37%) patients were female. The mean duration of ongoing infliximab originator treatment was 7.5 years (SD 3.6), and the majority of patients (298, 78%) were biologic treatment naïve prior to the ongoing infliximab treatment (Table 1). The extension study cohort consisted of 127 (33%) patients with CD, 80 (21%) with UC, 67 (18%) with SpA, 55 (15%) with RA, 20 (5%) with PsA and 31 (8%) with Ps (Table 1). In general, the two treatment groups were similar for major baseline demographic and disease characteristics, as shown in Table 1 (FAS) and in Table S1 (PPS), Appendix, page 3. Both the general and the disease‐specific measures indicated low disease activity at baseline (Table 1). Comparing the extension cohort to the main study cohort at randomization (week 0) did not demonstrate any major differences regarding disease distribution, demographics or disease characteristics (Tables S3 and S4). In the same way, comparing the dropouts with the included patients in each study group did not display any major differences between groups (Table S5).

**Table 1:** Demographics and characteristics at extension study baseline (week 52) (full analysis set)

	Maintenance group (n = 197)	Switch group (n = 183)
**Demographics**		
Age (years)	48.8 (14.9)	48 (14.3)
Female	64 (33%)	78 (43%)
Disease duration (years)	18.1 (10.6)	17.3 (10.4)
Duration of ongoing infliximab treatment (years)	7.7 (3.8)	7.4 (3.5)
**Previous biological therapy**		
TNF α inhibitors		
Not used	158 (80%)	140 (77%)
Used one	31 (16%)	33 (18%)
Used two	6 (3%)	10 (5%)
Used three or more	2 (1%)	0
Other biologicals	0	2 (1%)
Concomitant immunosuppressive therapy^a^	97 (49%)	75 (41%)
Concomitant use of prednisolone	5 (3%)	7 (4%)
**Diagnoses**		
Crohn's disease	65 (33%)	62 (34%)
Ulcerative colitis	42 (21%)	38 (21%)
Spondyloarthritis	38 (19%)	29 (16%)
Rheumatoid arthritis	27 (14%)	28 (15%)
Psoriatic arthritis	9 (5%)	11 (6%)
Psoriasis	16 (8%)	15 (8%)
**General baseline characteristics**		
Erythrocyte sedimentation rate (mm/h)	8 (4–17)	7 (4–15)
C‐reactive protein (mg/L)	2 (1–5)	2 (1–5)
Patient's global assessment of disease activity (0–10)	2 (0–4)	2 (0–3)
Physician's global assessment of disease activity (0–10)	2 (0–4)	2 (0–3)
EQ‐5D Index score	0.8 (0.2)	0.8 (0.2)
**Disease‐specific baseline characteristics**		
Crohn's disease		
Harvey‐Bradshaw index	1 (0–4)	1 (0.2–4)
Ulcerative colitis		
Partial Mayo score	0 (0–0)	0 (0–1)
Crohn's disease and ulcerative colitis		
Faecal calprotein (mg/kg)	79 (24–273)	56 (21–209)
Spondyloarthritis		
HLA‐B27 positive^b^	26/30 (87%)	19/21 (91%)
Bath Ankylosing Spondylitis Disease Activity Index	3.2 (1.8)	2.6 (1.6)
Ankylosing Spondylitis Disease Activity Score	1.9 (0.8)	1.7 (0.7)
Rheumatoid arthritis		
Anti‐citrullinated protein antibody positive	13/17 (77%)	17/21 (81%)
Rheumatoid factor positive^b^	17/22 (77%)	19/27 (70%)
Disease Activity Score in 28 joints with CRP	2.4 (0.9)	2.8 (0.9)
Psoriatic arthritis		
Disease Activity Score in 28 joints with CRP	2.1 (1.1)	2.9 (1.8)
Clinical Disease Activity Index	4.7 (3.4)	6.6 (10)
Simplified Disease Activity Index	4.8 (3.4)	6.9 (10.2)
Spondyloarthritis, rheumatoid arthritis and psoriatic arthritis		
Modified Health Assessment Questionnaire	0.4 (0.4)	0.4 (0.4)
Chronic plaque psoriasis		
Psoriasis Area and Severity Index	2.1 (1.4)	1.3 (1.2)

Data are n (%), mean (SD) or median (IQR). TNF, tumour necrosis factor; HLA, Human leukocyte antigen; EQ5D, EuroQol questionnaire time trade‐off (UK weighted). ^a^Immunosuppressive concomitant medication includes methotrexate, leflunomide, sulfasalazine, azathioprine and mercaptopurine. ^b^Data were missing for some patients.

**Figure 2:**
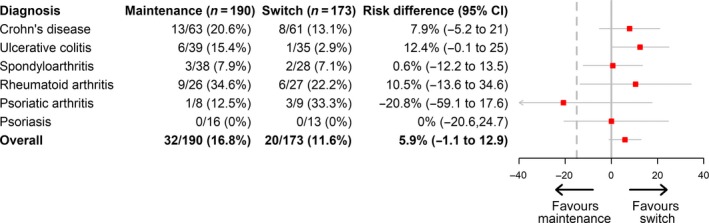
Forest plot of risk difference according to disease. The figure shows data for the per‐protocol set. Risk difference is adjusted for treatment duration of infliximab originator at extension study baseline (week 52).

Disease worsening occurred in 32 (16.8%) patients in the maintenance group and in 20 (11.6%) patients in the switch group (PPS; Fig. 2). The 95% confidence interval (CI) of the adjusted risk difference (5.9%) was −1.1 to 12.9, which was clearly within the prespecified noninferiority margin of −15%. The findings regarding risk of disease worsening in the FAS were consistent, as shown in Appendix, Figure S1. When comparing disease worsening after 26 weeks in patients switched from originator to biosimilar in the main study to results after 26 weeks in the current extension study, results were similar (Table S6).

At study end (week 78), 116 (61.1%) patients in the maintenance group and 117 (67.6%) patients in the switch group were in clinical remission. The adjusted rate difference was −4.9% (95% CI −13.4 to 3.7) (PPS; Figure S2, Appendix).

Disease state at baseline and changes in the generic disease variables and disease‐specific composite measures from extension phase baseline (week 52) to the end of follow‐up (week 78) were generally similar in both groups, both in the PPS (Table 2) and in the FAS (Appendix, Table S2).

**Table 2:** Secondary efficacy endpoints in the per‐protocol set

	Baseline extension study (52 weeks)		Study end (78 weeks)		Difference at 78 weeks (95% CI)
	Maintenance group (n = 190)	Switch group (n = 173)	Maintenance group (n = 190)	Switch group (n = 173)	
Change variables^a^					
Physician's global assessment of disease activity	1.26 (1.47)	1.11 (1.48)	1.45 (1.55)	1.15 (1.51)	0.13 (−0.13 to 0.4)
Patient's global assessment of disease activity	2.32 (2.07)	1.98 (1.9)	2.58 (2.26)	1.88 (1.96)	0.48 (0.16 to 0.8)
Erythrocyte sedimentation rate (mm/h), log10	0.93 (0.41)	0.88 (0.39)	0.89 (0.4)	0.86 (0.38)	0 (−0.05 to 0.05)
C‐reactive protein (mg/L), log10	0.34 (0.48)	0.32 (0.45)	0.31 (0.48)	0.33 (0.4)	−0.02 (−0.1 to 0.05)
Calprotectin (mg/kg), log10	1.98 (0.67)	1.87 (0.62)	2.12 (0.71)	1.92 (0.62)	0.2 (0.03 to 0.37)
Harvey‐Bradshaw Index (Crohn's disease)	2.49 (2.9)	2.77 (3.18)	2.93 (3.24)	2.44 (3.28)	0.57 (−0.2 to 1.33)
Partial Mayo Score (ulcerative colitis)	0.44 (1.07)	0.71 (1.1)	0.88 (1.55)	0.47 (0.82)	0.44 (−0.13 to 1.01)
ASDAS (spondyloarthritis)	1.9 (0.82)	1.69 (0.69)	2.13 (0.85)	1.79 (0.61)	0.2 (−0.06 to 0.46)
DAS28 (rheumatoid arthritis and psoriatic arthritis)	2.29 (0.97)	2.87 (1.26)	2.48 (1.54)	2.62 (1.16)	0.19 (−0.33 to 0.71)
CDAI (rheumatoid arthritis and psoriatic arthritis)	4.35 (3.23)	6.22 (6.61)	6.81 (7.47)	6.45 (6.74)	1.92 (−1.07 to 4.91)
SDAI (rheumatoid arthritis and psoriatic arthritis)	4.71 (3.25)	6.55 (6.69)	7.41 (7.95)	6.84 (6.79)	2.13 (−1.86 to 6.12)
PASI (chronic plaque psoriasis)	2.12 (1.39)	0.95 (0.85)	1.49 (0.89)	1.25 (0.88)	−0.28 (−0.87 to 0.31)
State variables^b^					
HBI remission (Crohn's disease)	46 (73%)	43 (70%)	41 (65%)	46 (75%)	−0.12 (−0.27 to 0.04)
PMS remission (ulcerative colitis)	37 (95%)	32 (91%)	32 (84%)	30 (86%)	−0.01 (−0.17 to 0.16)
ASDAS inactive disease (spondyloarthritis)	7 (18%)	8 (32%)	7 (18%)	6 (21%)	−0.05 (−0.25 to 0.15)
DAS28 remission status (rheumatoid arthritis and psoriatic arthritis)	22 (65%)	16 (46%)	20 (59%)	22 (61%)	−0.02 (−0.26 to 0.21)
CDAI remission status (rheumatoid arthritis and psoriatic arthritis)	12 (35%)	9 (26%)	9 (35%)	7 (25%)	0.08 (−0.16 to 0.33)
SDAI remission status (rheumatoid arthritis and psoriatic arthritis)	15 (44%)	14 (40%)	11 (42%)	8 (29%)	0.11 (−0.14 to 0.36)
ACR/EULAR remission status (rheumatoid arthritis and psoriatic arthritis)	10 (29%)	8 (23%)	7 (21%)	11 (31%)	−0.11 (−0.32 to 0.1)
PASI complete clearance (chronic plaque psoriasis)	2 (12%)	4 (31%)	1 (6%)	1 (8%)	−0.01 (−0.2 to 0.17)
PASI mild to moderate (chronic plaque psoriasis)	16 (100%)	13 (100%)	16 (100%)	13 (100%)	0 (0 to 0)
PASI remission (chronic plaque psoriasis)	15 (94%)	13 (100%)	16 (100%)	13 (100%)	0 (0 to 0)
Patient‐reported outcome measures					
SF‐36 physical functioning	84.18 (20.11)	83.26 (21.47)	83.25 (21)	83.15 (22.12)	−0.71(−2.78 to 1.37)
SF‐36 role limitation physical	67.34 (39.32)	67.02 (40.27)	66.64 (38.23)	70.78 (38.03)	−3.7 (−9.8 to 2.4)
SF‐36 pain	73.14 (23.2)	74.93 (22.57)	73.65 (21.54)	75.05 (22.05)	0.08 (−3.24 to 3.41)
SF‐36 general health	61.91 (23.1)	64.12 (24.55)	62.42 (23.09)	62.2 (23.61)	2.01 (−0.41 to 4.43)
SF‐36 emotional well‐being	78.33 (17.31)	77.77 (17.23)	79.25 (15.32)	79.01 (16.41)	0.37 (−1.85 to 2.6)
SF‐36 role limitation emotional	73.33 (37.56)	80.32 (33.04)	77.17 (34.67)	81.06 (32.46)	0.25 (−5.49 to 6)
SF‐36 social functioning	82.49 (22.13)	83.83 (23.34)	82.96 (19.97)	88.08 (17.67)	−3.54 (−6.79 to −0.28)
SF‐36 energy fatigue	53.49 (25.67)	53.11 (23.78)	52.3 (23.24)	54.74 (23.76)	−2.63 (−5.62 to 0.37)
SF‐36 physical component summary score	47.3 (9.61)	46.9 (10.8)	46.55 (10.13)	46.72 (10.69)	−0.39 (−1.61 to 0.83)
SF‐36 mental component summary score	48.03 (11.48)	49.03 (10.57)	48.89 (10.34)	50.19 (9.99)	−0.47 (−1.98 to 1.05)
EQ‐5D index	0.78 (0.21)	0.78 (0.23)	0.8 (0.19)	0.79 (0.22)	0.01(−0.02 to 0.04)
WPAI percent work missed due to specified problem (absenteeism)	7.37 (20.49)	4.51 (15.3)	8.2 (21.4)	6.89 (21.65)	0.95(−4.27 to 6.18)
WPAI percent work impaired while working due to specified problem (presenteeism)	20.35 (22.83)	14 (19.49)	16.85 (19.86)	14.7 (21)	2.31(−2.21 to 6.83)
WPAI percent overall work impairment due to specified problem	23.78 (26.6)	15.83 (22.11)	20.3 (24.09)	17.17 (24.31)	1.87 (−3.67 to 7.41)
WPAI percent activity impairment due to specified problem	26.47 (26.48)	22.88 (24.81)	25.33 (24.31)	21.6 (22.42)	1.87 (−1.97 to 5.71)
IBDQ total score (Crohn's disease and ulcerative colitis)	188.26 (27.97)	189.38 (27.76)	185.54 (28.3)	191.43 (27.08)	−4.79 (−9.21 to −0.36)
MHAQ (spondyloarthritis, rheumatoid arthritis, psoriatic arthritis)	0.38 (0.36)	0.36 (0.41)	0.39 (0.36)	0.35 (0.41)	0.05 (−0.03 to 0.12)
BASDAI (spondyloarthritis)	3.23 (1.76)	2.38 (1.44)	3.81 (1.67)	2.62 (1.6)	0.43 (−0.05 to 0.92)
RAID total score (rheumatoid arthritis)	2.78 (1.68)	2.82 (1.74)	2.83 (1.49)	2.48 (1.39)	0.31 (−0.29 to 0.92)
PsAID total score (psoriatic arthritis)	2.2 (1.7)	2.92 (2.3)	2.16 (2.04)	3.65 (2.43)	−0.26 (−1.19 to 0.68)
DLQI total score (chronic plaque psoriasis)	2.31 (4.63)	1.08 (2.14)	1.73 (3.25)	1.46 (2.44)	−0.31 (−1.16 to 0.55)


MHAQ, Modified Health Assessment Questionnaire; BASDAI, Bath Ankylosing Spondylitis Disease Activity Index; ASDAS, Ankylosing Spondylitis Disease Activity Score; DAS28, Disease Activity Score in 28 joints with CRP; CDAI, Clinical Disease Activity Index; SDAI, Simplified Disease Activity Index; ACR/EULAR, American College of Rheumatology/European League Against Rheumatism; PASI, Psoriasis Area and Severity Index; SF‐36, RAND Short Form Health Survey t‐scores using Norwegian norms; EQ‐5D, EuroQol questionnaire time trade‐off UK weighted; WPAI, Work Productivity and Impairment Questionnaire; IBDQ, Inflammatory Bowel Disease Questionnaire; RAID, Rheumatoid Arthritis Impact of Disease; PsAID, Psoriatic Arthritis Impact of Disease; DLQI, Dermatology Life Quality Index. ^a^Data are mean (SD) at baseline and mean (SD) change from baseline (follow‐up minus baseline). Difference is adjusted treatment difference of change from baseline with 95% CI. ^b^Data are N (%) of state at baseline and study end. Difference is adjusted treatment difference at study end.

**Figure 3:**
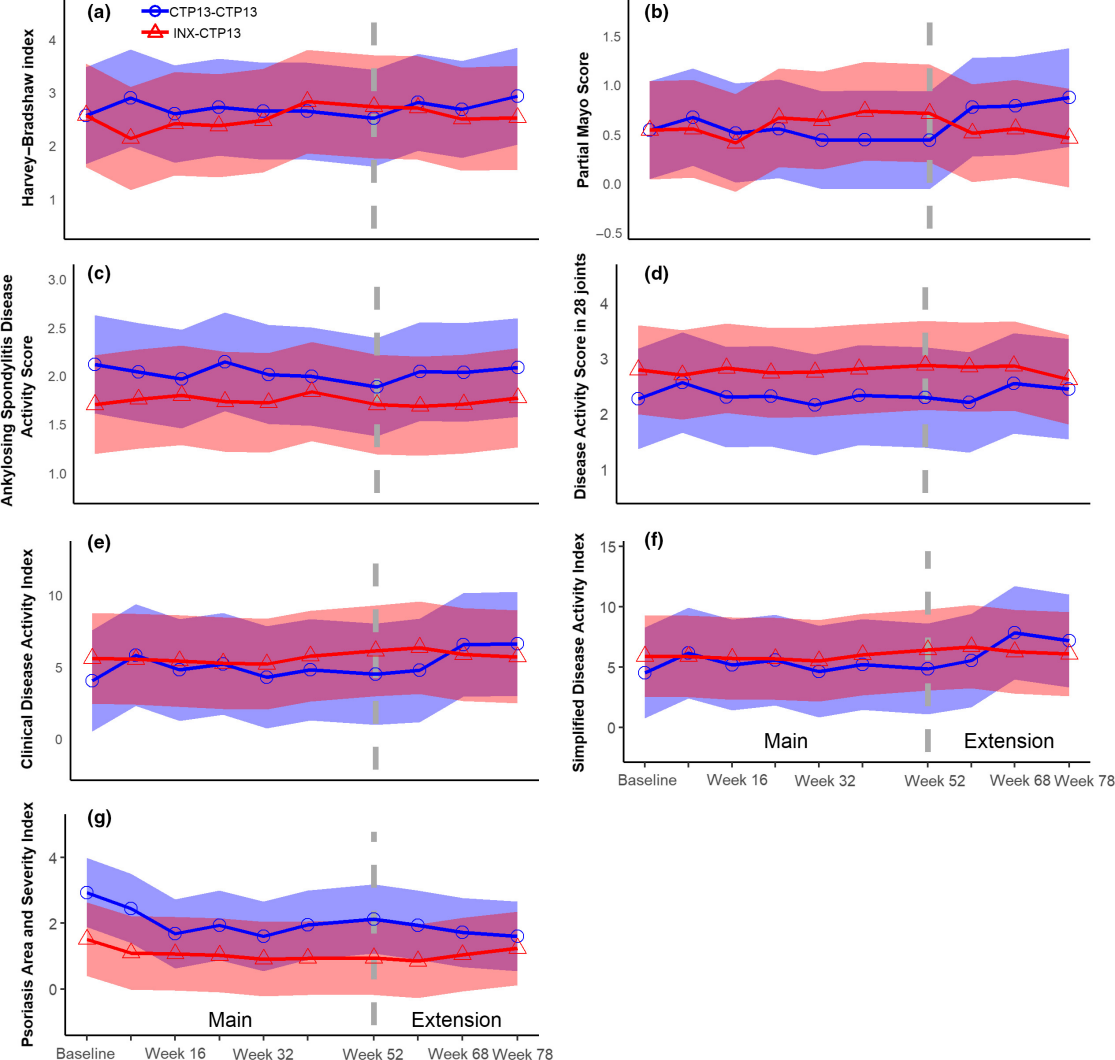
Change in disease‐specific composite measures during 78 weeks of follow‐up in the NOR‐SWITCH main and extension study in the per‐protocol set. (a) HBI for Crohn's disease. (b) PMS for UC. (c) Ankylosing Spondylitis Disease Activity Score for SpA. (d) Disease Activity Score in 28 joints with CRP for RA and PsA. (e) Clinical Disease Activity Index for RA and PsA. (f) Simplified Disease Activity Index for RA and PsA. (g) PASI for psoriasis. The coloured areas display the 95% confidence intervals from a mixed model adjusted for treatment duration of infliximab originator at main study baseline (week 0).

Figure 3 shows no differences between the two treatment groups for disease‐specific composite measures in each of the six diseases during follow‐up. Likewise, changes in PROMs were similar between the groups in the PPS (Table 2). However, we noted statistically significant differences for three of the end‐points in both the PPS and FAS (Tables 2 and S2, Appendix) (SF‐36 social functioning in favour of the maintenance group, and patient's global assessment of disease activity and IBDQ total score in favour of the switch group).

Time from the extension phase baseline to disease worsening (PPS; Figure S3, Appendix), occurrence of drug discontinuation (FAS; Table S2, Appendix) and time from baseline to drug discontinuation (FAS; Figure S4, Appendix page 7) were similar between groups.

Similar numbers of patients in the maintenance and switch groups reported at least one adverse event during follow‐up, 87 (44%) and 74 (40%), respectively (Table 3). The most frequent adverse events were infections. No deaths and no suspected unexpected serious adverse reactions occurred. Notably, there was no difference in the occurrence of infusion reactions between the two treatment arms.

**Table 3:** Treatment‐ emergent adverse events in the safety population

	Maintenance group (n = 197)	Switch group (n = 183)
Overview		
SUSAR	0	0
Serious adverse events	17/14 (7%)	9/8 (4%)
Adverse event	145/87 (44%)	118/74 (40%)
Adverse events leading to study drug discontinuation^a^	2 (1%)	1 (0.5%)
Most frequent treatment emerging adverse events		
Nasopharyngitis	19/19 (10%)	8/7 (4%)
Gastroenteritis	6/5 (3%)	3/3 (2%)
Urinary tract infection	7/7 (4%)	2/2 (1%)
Rash	3/3 (2%)	4/3 (2%)
Elevated liver enzymes	3/2 (1%)	3/2 (1%)
Iron deficiency	4/3 (2%)	2/1 (1%)
Influenza‐like illness	3/3 (2%)	2/2 (1%)
Infusion related reaction	3/3 (2%)	2/2 (1%)
Respiratory tract infection	4/4 (2%)	1/1 (1%)
Treatment emerging serious adverse events by system organ class		
Cardiac disorders	2/2 (1%)	1/1 (1%)
Gastrointestinal disorders	1/1 (1%)	1/1 (1%)
General disorders and administration site conditions	‐	1/1 (1%)
Infections and infestations	2/2 (1%)	3/3 (2%)
Musculoskeletal and connective tissue disorders	2/2 (1%)	–
Neoplasms benign, malignant and unspecified	1/1 (1%)	–
Nervous system disorders	1/1 (1%)	–
Renal and urinary disorders	1/1 (1%)	2/1 (1%)
Respiratory, thoracic and mediastinal disorders	1/1 (1%)	–
Surgical and medical procedures	3/3 (2%)	–

Data are number of events/number of patients (%). SUSAR, suspected unexpected serious adverse reaction. ^a^Patients could have other primary reason for study drug discontinuation.

Trough drug concentrations were similar in both groups during follow‐up (FAS; Figure S5, Appendix). ADAb were observed at any time‐point during the 26‐week extension study (week 52 to 78) in 5 (2.5%) patients in the maintenance group and in 4 (2.2%) patients in the switch group (FAS).

## Discussion

The NOR‐SWITCH study addresses the important issue of whether stable patients on infliximab can be safely switched to biosimilar CT‐P13, maintaining a prolonged response with minimal adverse effects and no increased immunogenicity. The main study showed noninferiority of switching from originator to biosimilar 14. The extension phase of this study addresses the need for independent clinical studies on switching, comparing patients on long‐term CT‐P13 treatment versus those being switched from infliximab originator to CT‐P13.

Disease worsening during the extension phase occurred at a similar rate in the two groups, with no significant difference amongst those switched at main study baseline and those switched at extension study baseline. The individual disease groups show a somewhat diverse pattern, but even without adjusting for multiple testing, none of these subgroups shows any significant difference between long‐term CT‐P13 treatment and a recent switch to CT‐P13. We warn against placing too much emphasis on findings in individual patient groups as patient numbers are small and no definite conclusions can be drawn for individual diseases in the NOR‐SWITCH extension study, as in the main trial. Concerns have been raised about borderline noninferiority in CD in the main study, but the subgroup analyses for CD and other diseases did not raise any concerns in this extension study. On the contrary, the occurrence of disease worsening was numerically lower in the switch versus the maintenance group overall and also in the CD subgroup.

The secondary end‐points in the current extension study showed small but significant differences between the study groups for patient global assessment, SF‐36 social functioning and IBDQ total score. There were no new safety signals during the extension study period, and adverse events were evenly distributed between study arms. Importantly, there was no difference in the occurrence of infusion reactions following a switch to CT‐P13, and these were rare occurrences in both study arms. This observation is in agreement with findings from the main NOR‐SWITCH study 14.

Immunogenicity analyses after switching between different versions of infliximab may be of particular interest, as this molecule is thought to be more immunogenic than other TNF‐α inhibitors 15. Thus, assessing ADAb to infliximab could be an especially sensitive test for altered immunogenicity after switching from originator to biosimilar agent. No significant differences in immunogenicity were detected during the entire 78‐week trial period. Serum drug levels remained very similar as were the ADAb detected in the two study arms. Thus, the current extension study data suggest that there is no increase in immunogenicity reaction over a prolonged treatment period after switching to CT‐P13. This is in agreement with data from the PLANETRA and PLANETAS extension studies 9, 10. It is also in agreement with previous publications suggesting that ADAb development is most frequent early after starting treatment 16.

The NOR‐SWITCH trial results serve as a reminder that disease worsening occurs in a substantial number of patients even when treatment has been stable over many years. This is also corroborated by real‐life DANBIO data 17. Disease worsening in an individual patient should therefore not necessarily be attributed to a recent switch to a specific biosimilar. It is notable that the rate of disease worsening over 6 months in the extension study is approximately half of the disease worsening rate seen over the 12‐month observation in the main study, indicating a constant rate of disease worsening in stable, long‐term patients.

The nocebo effect is a concern when switching from originator to biosimilar medicines 18, 19. The DANBIO register provides switching data after a mandated, nonmedical switch was done nationwide in Denmark, following a decision by Danish health authorities 17, 20. One might reasonably expect the nocebo effect to be very pronounced under these circumstances. Retention rates in the DANBIO data were slightly lower in the switched patients than in a historical cohort, and the authors suggest this might be due to a nocebo effect. Overall, however, the DANBIO switch data show a surprisingly moderate nocebo effect 17. In the same way, Razanskaite *et al*. 21 demonstrated no deterioration in clinical and patient‐reported outcomes after an open prospective switch in 143 CD and UC patients. On the other hand, a recently published open prospective switch study including 222 patients with RA, SpA and PsA displayed a 25% retention rate 6 months after switching due to PROM, demonstrating a significant nocebo effect 19. Our patients were volunteers and remained blinded to what treatment had been given during the main study period. Perhaps for these reasons, we do not see a clearly delineated nocebo effect in the NOR‐SWITCH patients.

A strength of the current extension study is the large proportion of patients included from the main study period. The main reason patients did not continue into the extension phase was that some study centres did not have the resources necessary to participate further. Only eight and nine patients in each study arm, respectively, did not enter the extension study for reasons other than study centre nonparticipation. Data collection from participating patients was comprehensive, with immunogenicity assessed at every visit.

The main weakness of the current study is that it does not allow for statistically meaningful analysis within each disease. The primary outcome is the occurrence of disease worsening, which relies on differing composite measures for each disease. This was also highlighted as a limitation and extensively discussed in the main publication. Also, the extension study period is fairly short with just 26 weeks, giving us a total of 18 months of observation time for patients switched to CT‐P13. We cannot rule out the possibility that there is a gradual loss of effect over longer periods of time. Extension studies with etanercept biosimilar have shown a reassuringly robust response up to 2 years 11, though this is a low‐immunogenicity agent.

We conclude that after 6 months, patients switched to CT‐P13 from the originator do as well as patients on CT‐P13 treatment for 18 months. Also, a similar efficacy and safety profile can be expected 18 months after a switch to biosimilar CT‐P13 as after 12 months of continuous originator infliximab or 12 months of biosimilar CT‐P13. We did not observe any increase in immunogenicity, SAEs and no loss of efficacy during the extension phase.

The extension of the NOR‐SWITCH study supports that switching from originator to CT‐P13 does not have a negative impact on efficacy, safety and immunogenicity. These results can be expected to enhance confidence in the use of biosimilars in patients with rheumatic diseases, IBD and psoriasis 22. This should reduce the cost of biologic treatments. The consequence for health policies will hopefully be that patients in many areas of the world will have better access to biologic agents, at a lower cost than today. Further, with lower cost for biologic medicines, funds might become available for new innovative drugs in current nonresponders.

## Conflict of interest statements

GLG reports personal fees from AbbVie, Biogen, Eli Lilly, MSD, Novartis, Pfizer, Roche, Sandoz, Orion Pharma, Celltrion and Boehringer Ingelheim. KKJ reports personal fees from Tillott, Intercept and Celltrion. NB reports personal fees received from Orion Pharma, Roche, Napp Pharmaceuticals, Pfizer and Takeda. ICO reports grants from Norwegian Ministry of Health and Care Services during the conduct of the study and personal fees from Pfizer. EAH reports grants from AbbVie, Pfizer, UCB, Roche and MSD. IPB reports personal fees from AbbVie, MSD, Takeda, Hospira and Ferring. KEAL reports grants from MSD and personal fees from Takeda, Orion, AbbVie, Pfizer and MSD. KST reports personal fees and nonfinancial support from AbbVie, Orion, Novartis, Janssen, Celgene, Mundipharma, Pfizer, MSD and Shire and nonfinancial support from CSL Behring. CM reports personal fees from Novartis Norge AS, LEO Pharma AS, ACO Hud Norge AS, Celgene AS, AbbVie and Galderma Nordic AB. JJ reports personal fees from MSD, AbbVie, Celltrion, Orion Pharma, Takeda, Napp Pharm, AstroPharma, Hikma and Pfizer. TKK reports grants from Norwegian Ministry of Health and Care Services during the conduct of the study and personal fees from AbbVie, Biogen, BMS, Boehringer Ingelheim, Celltrion, Eli Lilly, Epirus, Janssen, Merck‐Serono, MSD, Mundipharma, Novartis, Oktal, Orion Pharma, Hospira/Pfizer, Roche, Sandoz and UCB Pharma.

## Author contributions

TKK was the principal investigator who conceived and designed the study, interpreted data and drafted and critically revised the report. He has had full access to all the data in the study.

GLG contributed to the study design, oversaw the implementation of the study at all rheumatology centres, provided support to study personnel at each rheumatology site during the study, contributed to data interpretation and drafted and critically revised the manuscript. She took final responsibility for the decision to submit for publication.

KKJ contributed to the study design and data interpretation, oversaw the implementation of the study at all gastroenterology centres, provided study support to all gastroenterology centres throughout the study and drafted and critically revised the manuscript.

JJ, CM, EAH and KST contributed to the study conception, helped design the study and interpret the data and critically revised the manuscript.

JS was study statistician. He performed the analyses, contributed to data interpretation and drafted and critically revised the manuscript.

ICO was study statistician. He contributed to the study conception, helped design the study, led the development of the electronic CRF and contributed support to all sites during the study. He performed some analyses, contributed to data interpretation and drafted and critically revised the manuscript.

NB helped design the study, facilitated laboratory analyses of infliximab and ADAb, developed the assay for ADAb and planned and organized the study biobank. He helped interpret data and critically revised the manuscript.

KEAL contributed to the study conception and design, in particular contributing to planning and interpretation of the immunogenicity analyses, and critically revised the manuscript.

ML oversaw the implementation of the study at all dermatology centres, contributed to data interpretation and drafted and critically revised the report.

IPB, BTSF, SK and KR contributed to the study conception, helped design the study and critically revised the report.

DJW developed the assay for infliximab drug levels, provided reagents for anti‐drug antibody analysis, was instrumental in setting up the infrastructure for these analyses, helped interpret data and critically revised the report.

RK performed the immunogenicity analyses, helped organize the study biobank and critically revised the report.

JH, BG and KW were user representatives and contributed to the design of the trial and data collection from a service user perspective, were crucial in distributing study information to relevant patient populations, and critically revised the report.

All the remaining authors were main investigators at each study site, who implemented the study at their site, collected the data and critically revised the report.

All authors made substantial contributions to the conception or design of the study, or the acquisition, analysis or interpretation of the data; commented on drafts of this paper; and approved the final version. All authors agree to be accountable for all aspects of the work in ensuring that questions related to the accuracy or integrity of any part of the work are appropriately investigated and resolved.

## Supporting information


**Appendix**
**Figure S1.** Disease worsening in the Full Analysis Set.
**Figure S2.** Remission in the Per Protocol Set.
**Figure S3.** Time from baseline extension study (week 52) to disease worsening in the Per Protocol Set.
**Figure S4.** Time from baseline extension study (week 52) to drug discontinuation in the Full Analysis Set.
**Figure S5.** Serum trough concentration from baseline main study (week 0) to end of extension study (week 78) in the safety population.
**Table S1**. Demographics and characteristics at extension study baseline (week 52) (Per Protocol Set).
**Table S2**. Secondary efficacy endpoints in the Full Analysis Set.
**Table S3**. Demographics and baseline characteristics in main and extension study (Full Analysis Set).
**Table S4**. Demographics and characteristics in the extension population at baseline main study (week 0) and baseline extension study (week 52) (Full Analysis Set).
**Table S5**. Comparison of patients entering extension study versus those that did not (dropouts).
**Table S6**. Comparison of disease worsening at 26 weeks of matched subjects in main and extension period for Remsima and Remicade groups (PPS).
**Data S1.** Study protocol
