# Supplementary material for: Long‐term efficacy and safety of biosimilar infliximab (CT‐P13) after switching from originator infliximab: open‐label extension of the NOR‐SWITCH trial
Source: J Intern Med. 2019 Apr 12;285(6):653–69. doi: 10.1111/joim.12880 (PMC6850326; doi:10.1111/joim.12880)
Supplement: Supplementary file 1 — Appendix Figure S1. Disease worsening in the Full Analysis Set. Figure S2. Remission in the Per Protocol Set. Figure S3. Time from baseline extension study (week 52) to disease worsening in the Per Protocol Set. Figure S4. Time from baseline extension study (week 52) to drug discontinuation in the Full Analysis Set. Figure S5. Serum trough concentration from baseline main study (week 0) to end of extension study (week 78) in the safety population. Table S1. Demographics and characteristics at extension study baseline (week 52) (Per Protocol Set). Table S2. Secondary efficacy endpoints in the Full Analysis Set. Table S3. Demographics and baseline characteristics in main and extension study (Full Analysis Set). Table S4. Demographics and characteristics in the extension population at baseline main study (week 0) and baseline extension study (week 52) (Full Analysis Set). Table S5. Comparison of patients entering extension study versus those that did not (dropouts). Table S6. Comparison of disease worsening at 26 weeks of matched subjects in main and extension period for Remsima and Remicade groups (PPS). Data S1. Study protocol [file JOIM-285-653-s001.docx]

Supplementary appendix

Supplement to: Goll GL, Jørgensen KK, Sexton J, et al. **Long-term efficacy and safety of biosimilar infliximab (CT-P13) after switching from originator infliximab: Open-label extension of the NOR-SWITCH trial**

**Table of contents**

Section 1. Supplementary tables

Section 2. Supplementary figures

Section 3. Study protocol

**Section 1. Supplementary tables**

| Table S1. Demographics and characteristics at extension study baseline (week 52) (per-protocol Set) | | |
| --- | --- | --- |
|  | **Maintenance**  **Group**  **(n=190)** | **Switch**  **Group**  **(n=173)** |
| **Demographics** |  |  |
| Age (years) | 49.1 (15) | 48.3 (14.5) |
| Female | 63 (33%) | 74 (43%) |
| Disease duration (years) | 18.1 (10.6) | 17.3 (10.5) |
| Duration of ongoing infliximab treatment (years) | 7.7 (3.8) | 7.4 (3.6) |
| **Previous biological therapy** |  |  |
| TNF α inhibitors |  |  |
| Not used | 151 (80%) | 134 (78%) |
| Used one | 31 (16%) | 32 (19%) |
| Used two | 6 (3%) | 7 (4%) |
| Used three or more | 2 (1%) | 0 |
| Other biologicals | 0 | 2 (1%) |
| Concomitant immunosuppressive therapy* | 93 (49%) | 71 (41%) |
| Concomitant use of prednisolone | 5 (3%) | 7 (4%) |
| **Diagnoses** |  |  |
| Crohn's disease | 63 (33%) | 61 (35%) |
| Ulcerative colitis | 39 (21%) | 35 (20%) |
| Spondyloarthritis | 38 (20%) | 28 (16%) |
| Rheumatoid arthritis | 26 (14%) | 27 (16%) |
| Psoriatic arthritis | 8 (4%) | 9 (5%) |
| Psoriasis | 16 (8%) | 13 (8%) |
| **General baseline characteristics** |  |  |
| Erythrocyte sedimentation rate (mm/h) | 8.5 (4-17) | 8 (4-16) |
| C-reactive protein (mg/L) | 2 (1-5) | 2 (1-5) |
| Patient's global assessment of disease activity (0-10) | 2 (0.2-4) | 2 (0-3) |
| Physician's global assessment of disease activity (0-10) | 2 (0.2-4) | 2 (0-3) |
| EQ-5D Index score | 0.8 (0.2) | 0.8 (0.2) |
| **Disease-specific baseline characteristics** |  |  |
| Crohn's disease |  |  |
| Harvey-Bradshaw index | 1 (0-4) | 1 (0-4) |
| Ulcerative colitis |  |  |
| Partial Mayo score | 0 (0-0) | 0 (0-1) |
| Crohn's disease and ulcerative colitis |  |  |
| Faecal calprotein (mg/kg) | 85 (24-279) | 57 (21-201) |
| Spondyloarthritis |  |  |
| HLA-B27 positive^β^ | 26/30 (87%) | 18/20 (90%) |
| Bath Ankylosing Spondylitis Disease Activity Index | 3.2 (1.8) | 2.4 (1.4) |
| Ankylosing Spondylitis Disease Activity Score | 1.9 (0.8) | 1.7 (0.7) |
| Rheumatoid arthritis |  |  |
| Anti-citrullinated protein antibody positive | 13/16 (81%) | 17/21 (81%) |
| Rheumatoid factor positive^β^ | 17/21 (81%) | 19/26 (73%) |
| Disease Activity Score in 28 joints with CRP | 2.4 (0.9) | 2.9 (0.9) |
| Psoriatic arthritis |  |  |
| Disease Activity Score in 28 joints with CRP | 2 (1.2) | 3.1 (1.9) |
| Clinical Disease Activity Index | 4.1 (3.2) | 7.8 (10.9) |
| Simplified Disease Activity Index | 4.3 (3.3) | 8 (11) |
| Spondyloarthritis, rheumatoid arthritis and psoriatic arthritis |  |  |
| Modified Health Assessment Questionnaire | 0.4 (0.4) | 0.4 (0.4) |
| Chronic plaque arthritis |  |  |
| Psoriasis Area and Severity Index | 2.1 (1.4) | 1 (0.9) |
| Data are n (%), mean (SD) or median (IQR). TNF=tumour necrosis factor. HLA=Human leukocyte antigen. EQ5D=EuroQol questionnaire time trade-off (UK weighted). *Immunosuppressive concomitant medication includes methotrexate, leflunomide, sulfasalazine, azathioprine and mercaptopurine. ^β^Data were missing for some patients. | | |

| Table S2. Secondary efficacy endpoints in the full-analysis set | | | | | |
| --- | --- | --- | --- | --- | --- |
|  | **Baseline extension study (52 weeks)** | | **Study end**  **(78 weeks)** | | |
|  | **Maintenance group (n=197)** | **Switch group (n=183)** | **Maintenance group**  **(n=197)** | **Switch group**  **(n=183)** | **Difference at 78 weeks (95% CI)** |
| **Change variables*** |  |  |  |  |  |
| Physician's global assess-ment of disease activity | 1.26  (1.47) | 1.11  (1.48) | 1.45  (1.55) | 1.15 (1.51) | 0.13 (-0.13 to 0.4) |
| Patient's global assessment of disease activity | 2.32  (2.07) | 1.98  (1.9) | 2.58  (2.26) | 1.88 (1.96) | 0.48 (0.16 to 0.8) |
| Erythrocyte sedimentation rate (mm/h), log10 | 0.93  (0.41) | 0.88  (0.39) | 0.89  (0.4) | 0.86 (0.38) | 0 (-0.05 to 0.05) |
| C-reactive protein (mg/L), log10 | 0.34  (0.48) | 0.32  (0.45) | 0.31  (0.48) | 0.33 (0.4) | -0.02 (-0.1 to 0.05) |
| Calprotectin (mg/kg), log10 | 1.98  (0.67) | 1.87  (0.62) | 2.12  (0.71) | 1.92 (0.62) | 0.2 (0.03 to 0.37) |
| Harvey-Bradshaw Index (Crohn's disease) | 2.49  (2.9) | 2.77  (3.18) | 2.93  (3.24) | 2.44 (3.28) | 0.57 (-0.2 to 1.33) |
| Partial Mayo Score (ulcerative colitis) | 0.44  (1.07) | 0.71  (1.1) | 0.88  (1.55) | 0.47 (0.82) | 0.44 (-0.13 to 1.01) |
| ASDAS (spondyloarthritis) | 1.9  (0.82) | 1.69  (0.69) | 2.13  (0.85) | 1.79 (0.61) | 0.2 (-0.06 to 0.46) |
| DAS28 (rheumatoid arthritis and psoriatic arthritis) | 2.29  (0.97) | 2.87  (1.26) | 2.48  (1.54) | 2.62 (1.16) | 0.19 (-0.33 to 0.71) |
| CDAI (rheumatoid arthritis and psoriatic arthritis) | 4.35  (3.23) | 6.22  (6.61) | 6.81  (7.47) | 6.45 (6.74) | 1.92 (-1.07 to 4.91) |
| SDAI (rheumatoid arthritis and psoriatic arthritis) | 4.71  (3.25) | 6.55  (6.69) | 7.41  (7.95) | 6.84 (6.79) | 2.13 (-1.86 to 6.12) |
| PASI (chronic plaque psoriasis) | 2.12  (1.39) | 0.95  (0.85) | 1.49  (0.89) | 1.25 (0.88) | -0.28 (-0.87 to 0.31) |
| **State variables**† |  |  |  |  |  |
| HBI remission (Crohn's disease) | 46  (73%) | 43  (70%) | 41  (65%) | 46  (75%) | -0.12 (-0.27 to 0.04) |
| PMS remission (ulcerative colitis) | 37  (95%) | 32  (91%) | 32  (84%) | 30  (86%) | -0.01 (-0.17 to 0.16) |
| ASDAS inactive disease (spondyloarthritis) | 7  (18%) | 8  (32%) | 7  (18%) | 6  (21%) | -0.05 (-0.25 to 0.15) |
| DAS28 remission status (rheumatoid arthritis and psoriatic arthritis) | 22  (65%) | 16  (46%) | 20  (59%) | 22  (61%) | -0.02 (-0.26 to 0.21) |
| CDAI remission status (rheumatoid arthritis and psoriatic arthritis) | 12  (35%) | 9  (26%) | 9  (35%) | 7  (25%) | 0.08 (-0.16 to 0.33) |
| SDAI remission status (rheumatoid arthritis and psoriatic arthritis) | 15  (44%) | 14  (40%) | 11  (42%) | 8  (29%) | 0.11 (-0.14 to 0.36) |
| ACR/EULAR remission status (rheumatoid arthritis and psoriatic arthritis) | 10  (29%) | 8  (23%) | 7  (21%) | 11  (31%) | -0.11 (-0.32 to 0.1) |
| PASI complete clearance (chronic plaque psoriasis) | 2.12  (1.39) | 0.95  (0.85) | 1.49  (0.89) | 1.25 (0.88) | -0.28 (-0.87 to 0.31) |
| PASI mild to moderate (chronic plaque psoriasis) | 16  (100%) | 13  (100%) | 16  (100%) | 13 (100%) | 0 (0 to 0) |
| PASI remission (chronic plaque psoriasis) | 15  (94%) | 13  (100%) | 16  (100%) | 13 (100%) | 0 (0 to 0) |
| Study drug discontinuation |  |  | 0  (0%) | 4  (2%) | 0.02 (-0.01 to 0.05) |
| **Patient-reported outcome measures** | | | | | |
| SF-36 physical functioning | 84.18  (20.11) | 83.26 (21.47) | 83.25  (21) | 83.15 (22.12) | -0.71 (-2.78 to 1.37) |
| SF-36 role limitation physical | 67.34  (39.32) | 67.02 (40.27) | 66.64  (38.23) | 70.78 (38.03) | -3.7 (-9.8 to 2.4) |
| SF-36 pain | 73.14  (23.2) | 74.93 (22.57) | 73.65  (21.54) | 75.05 (22.05) | 0.08 (-3.24 to 3.41) |
| SF-36 general health | 61.91  (23.1) | 64.12 (24.55) | 62.42  (23.09) | 62.2 (23.61) | 2.01 (-0.41 to 4.43) |
| SF-36 emotional well-being | 78.33  (17.31) | 77.77 (17.23) | 79.25  (15.32) | 79.01 (16.41) | 0.37 (-1.85 to 2.6) |
| SF-36 role limitation emotional | 73.33  (37.56) | 80.32 (33.04) | 77.17  (34.67) | 81.06 (32.46) | 0.25 (-5.49 to 6) |
| SF-36 social functioning | 82.49  (22.13) | 83.83 (23.34) | 82.96  (19.97) | 88.08 (17.67) | -3.54 (-6.79 to -0.28) |
| SF-36 energy fatigue | 53.49  (25.67) | 53.11 (23.78) | 52.3  (23.24) | 54.74 (23.76) | -2.63 (-5.62 to 0.37) |
| SF-36 physical component summary score | 47.3  (9.61) | 46.9  (10.8) | 46.55  (10.13) | 46.72 (10.69) | -0.39 (-1.61 to 0.83) |
| SF-36 mental component summary score | 48.03  (11.48) | 49.03 (10.57) | 48.89  (10.34) | 50.19 (9.99) | -0.47 (-1.98 to 1.05) |
| EQ-5D index | 0.78  (0.21) | 0.78  (0.23) | 0.8  (0.19) | 0.79 (0.22) | 0.01 (-0.02 to 0.04) |
| WPAI percent work missed due to specified problem (absenteeism) | 7.37  (20.49) | 4.51  (15.3) | 8.2  (21.4) | 6.89 (21.65) | 0.95 (-4.27 to 6.18) |
| WPAI percent work impaired while working due to specified problem (presenteeism) | 26.47  (26.48) | 22.88 (24.81) | 25.33  (24.31) | 21.6 (22.42) | 1.87 (-1.97 to 5.71) |
| WPAI percent overall work impairment due to specified problem | 23.78  (26.6) | 15.83 (22.11) | 20.3  (24.09) | 17.17 (24.31) | 1.87 (-3.67 to 7.41) |
| WPAI percent activity impairment due to specified problem | 26.47  (26.48) | 22.88 (24.81) | 25.33  (24.31) | 21.6 (22.42) | 1.87 (-1.97 to 5.71) |
| IBDQ total score (Crohn's disease and ulcerative colitis) | 188.26  (27.97) | 189.38 (27.76) | 185.54  (28.3) | 191.43 (27.08) | -4.79 (-9.21 to -0.36) |
| MHAQ (spondyloarthritis, rheumatoid arthritis, psoriatic arthritis) | 0.38  (0.36) | 0.36  (0.41) | 0.39  (0.36) | 0.35 (0.41) | 0.05 (-0.03 to 0.12) |
| BASDAI (spondyloarthritis) | 3.23  (1.76) | 2.38  (1.44) | 3.81  (1.67) | 2.62 (1.6) | 0.43 (-0.05 to 0.92) |
| RAID total score (rheumatoid arthritis) | 2.78  (1.68) | 2.82  (1.74) | 2.83  (1.49) | 2.48 (1.39) | 0.31 (-0.29 to 0.92) |
| PsAID total score (psoriatic arthritis) | 2.2  (1.7) | 2.92  (2.3) | 2.16  (2.04) | 3.65 (2.43) | -0.26 (-1.19 to 0.68) |
| DLQI total score (chronic plaque psoriasis) | 2.31  (4.63) | 1.08  (2.14) | 1.73  (3.25) | 1.46 (2.44) | -0.31 (-1.16 to 0.55) |
| MHAQ=Modified Health Assessment Questionnaire. BASDAI=Bath Ankylosing Spondylitis Disease Activity Index. ASDAS=Ankylosing Spondylitis Disease Activity Score. DAS28=Disease Activity Score in 28 joints with CRP. CDAI=Clinical Disease Activity Index. SDAI= Simplified Disease Activity Index.  ACR/EULAR=American College of Rheumatology/European League Against Rheumatism. PASI=Psoriasis Area and Severity Index. SF-36=RAND Short Form Health Survey t-scores using Norwegian norms. EQ-5D=EuroQol questionnaire time trade-off UK weighted. WPAI=Work Productivity and Impairment Questionnaire. IBDQ=Inflammatory Bowel Disease Questionnaire. RAID=Rheumatoid Arthritis Impact of Disease. PsAID=Psoriatic Arthritis Impact of Disease. DLQI=Dermatology Life Quality Index. *Data are mean (SD) at baseline and mean (SD) change from baseline (follow-up minus baseline). Difference is adjusted treatment difference of change from baseline with 95% CI. †Data are N (%) of state at baseline and study end. Difference is adjusted treatment difference at study end. | | | | | |

**Section3. Supplementary figures**

**Figure S1 Disease worsening in the Full Analysis Set**


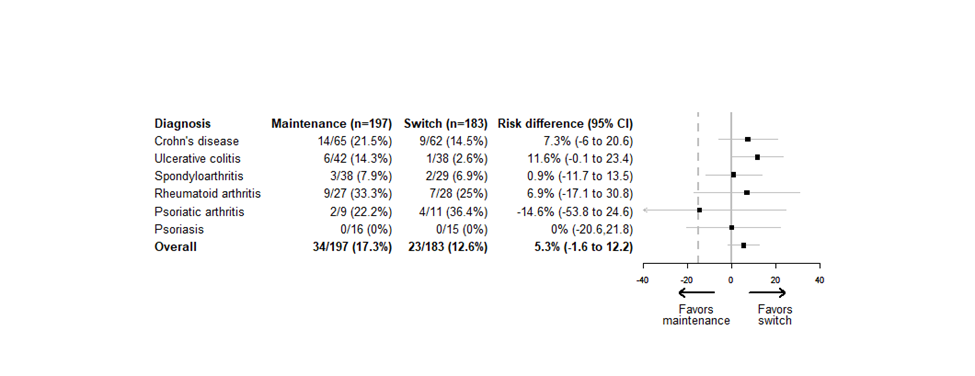


**Figure S2 Remission in the Per Protocol Set**


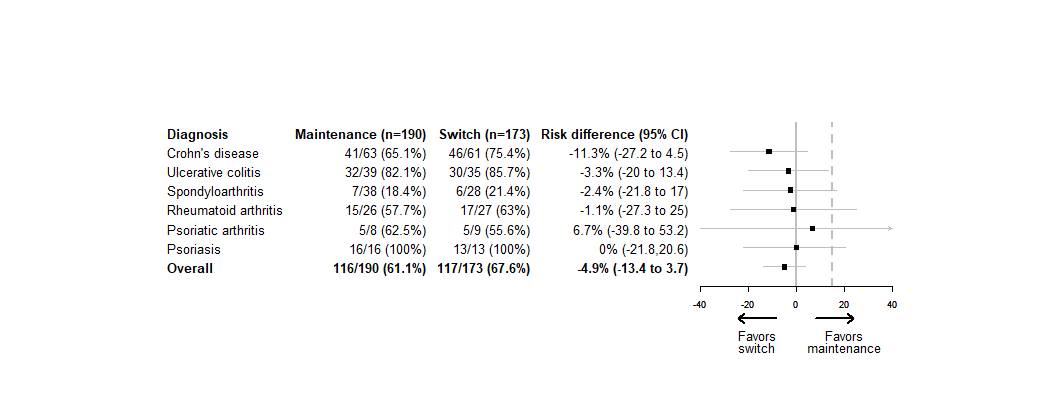


**Figure S3 Time from baseline extension study (week 52) to disease worsening in the Per Protocol Set**


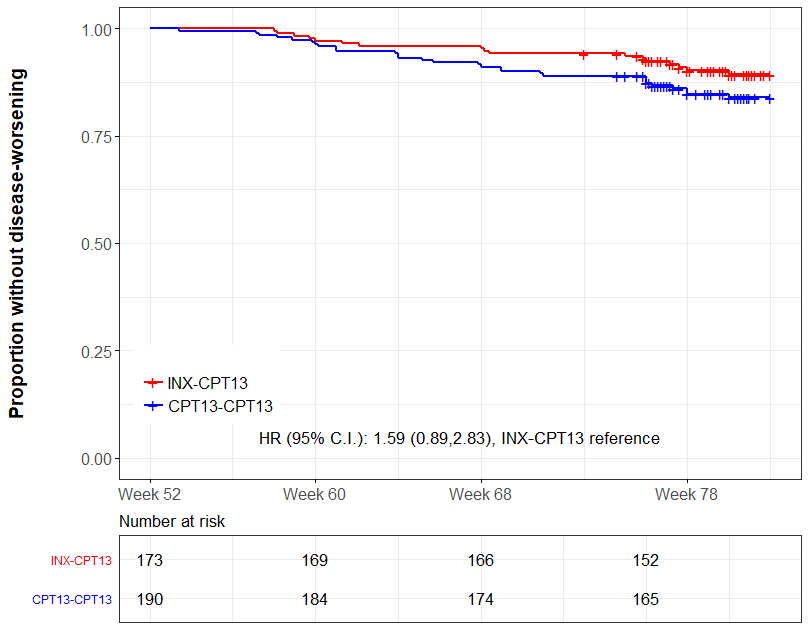


**Figure S4 Time from baseline extension study (week 52) to drug discontinuation in the Full Analysis Set**


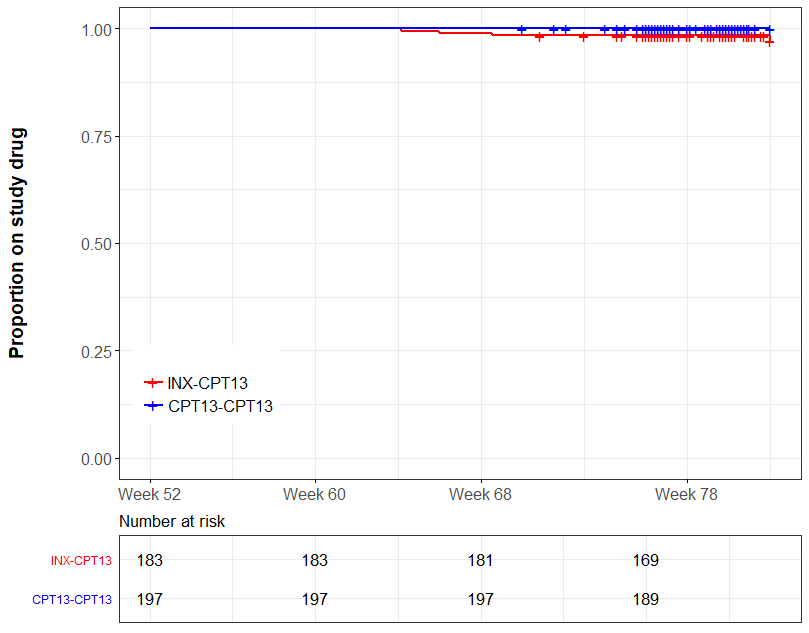


**Figure S5 Serum trough concentration from baseline main study (week 0) to end of extension study (week 78) in the safety population**


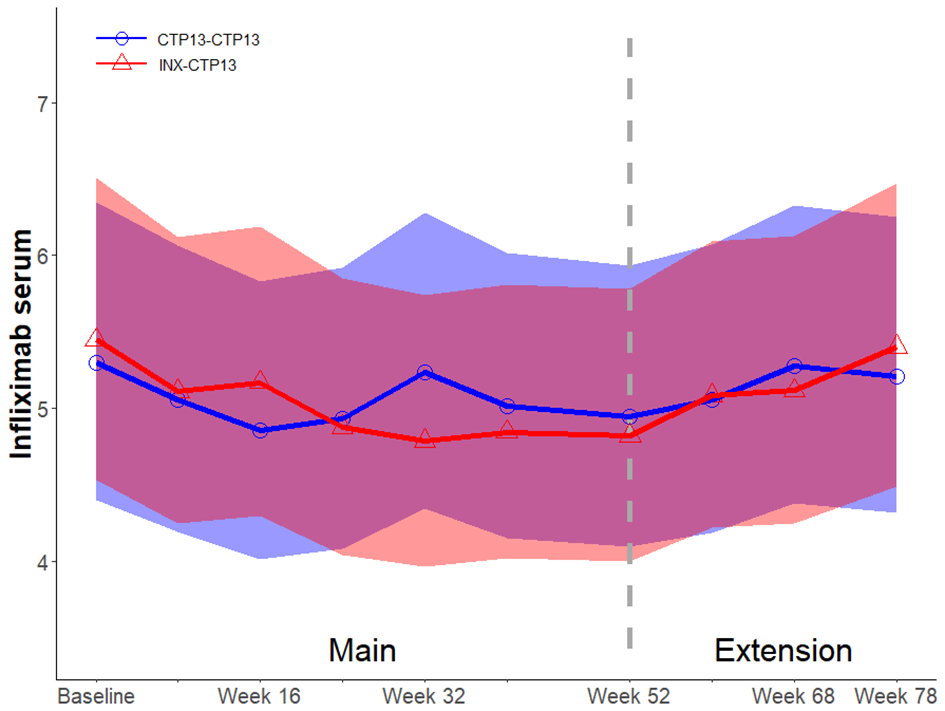


Section 3. Study protocol

A randomized, double-blind, parallel-group study to

evaluate the safety and efficacy of switching from innovator infliximab to biosimilar infliximab

compared with continued treatment with innovator infliximab in patients with rheumatoid arthritis, spondyloarthritis, psoriatic arthritis, ulcerative colitis, Crohn’s disease and chronic plaque psoriasis

The NOR-SWITCH study

**Protocol Identification Number:**  DIA2014-1

**EudraCT Number:** 2014-002056-40

| **SPONSOR:** | **Diakonhjemmet Hospital AS**  Contact person:  Professor Kåre Birger Hagen, PhD  Box 23 Vinderen, 0319 Oslo  Tel : 22 45 15 00  E-mail: k.b.hagen@medisin.uio.no |
| --- | --- |

| **PRINCIPAL INVESTIGATOR (PI):** | **Tore K. Kvien, MD PhD**  Dept. of Rheumatology  Diakonhjemmet Hospital, Box 23 Vinderen, 0319 Oslo  Tel:  E-mail: t.k.kvien@medisin.uio.no |
| --- | --- |

| **PROTOCOL VERSION NO. 2.0 21-07-2015** |
| --- |
| **Second Amendment** |

# CONTACT details

| **Sponsor:** | **Diakonhjemmet Hospital AS**  Contact person:  Professor Kåre Birger Hagen, PhD  Box 23 Vinderen, NO-0319 Oslo  Tel :+47 22 45 15 00  E-mail: k.b.hagen@medisin.uio.no |
| --- | --- |
| **Coordinating Investigator:** | **Tore K. Kvien, MD PhD**  Dept. of Rheumatology  Diakonhjemmet Hospital, Box 23 Vinderen, NO-0319 Oslo  Tel:+47 22 45 15 00  E-mail: t.k.kvien@medisin.uio.no |
| **Clinical Coordinator** | **Espen A. Haavardsholm, MD, PhD**  Dept. of Rheumatology  Diakonhjemmet Hospital, Box 23 Vinderen, NO-0319 Oslo  Tel: +47 22 45 15 00  E-mail: [e.a.haavardsholm@medisin.uio.no](mailto:e.a.haavardsholm@medisin.uio.no) |
| **Biostatistician** | **Inge Christoffer Olsen, PhD**  Dept. of Rheumatology  Diakonhjemmet Hospital, Box 23 Vinderen, NO-0319 Oslo  Tel: +47 22 45 15 00  E-mail: inge.christofferolsen@diakonsyk.no |
| **Participating centers:** | Diakonhjemmet Sykehus, Oslo  Universitetssykehuset i Nord-Norge, Tromsø  St. Olavs Hospital, Trondheim  Haukeland Universitetssykehus Helse Bergen HF, Bergen  Sørlandet Sykehus Kristiansand HF, Kristiansand  Sørlandet sykehus HF, Arendal  Haugesund Sanitetsforenings Revmatismesykehus, Haugesund  Martina Hansens Hospital, Sandvika  Sykehuset Østfold HF, Fredrikstad  Sykehuset Østfold HF, Moss  Revmatismesykehuset, Lillehammer  Betanien Hospital, Skien  Nordlandssykehuset HF, Bodø  Akershus Universitetssykehus, Lørenskog  Oslo Universitetssykehus, Oslo  Sykehuset Telemark, Skien  Ålesund sjukehus, Ålesund  Sykehuset Innlandet, Hamar  Sykehuset Innlandet, Gjøvik  Sykehuset Innlandet, Elverum  Sykehuset Innlandet, Lillehammer  Sykehuset Innlandet, Kongsvinger  Helse Nord-Trøndelag Sykehuset Levanger, Levanger  Bærum Sykehus, Bærum  Helgelandssykehuset, Mo i Rana  Helse Fonna HF Haugesund sjukehus, Haugesund  Helse Førde HF, Førde  Lovisenberg sykehus, Oslo  Nordlandssykehuset, Bodø  Sykehuset Vestfold, Tønsberg |
| **Monitor:** | **Department of Clinical Research Support, Oslo University Hospital**  Kirkeveien 166, Søsterhjemmet, Oslo  Tel : +47 23 01 68 81 |
| **Central laboratory** | **Department of Medical Biochemistry, Oslo University Hospital Radiumhospitalet**  Ullernschausseen 70, NO-0310 Oslo  Fax: +47 22 73 07 25 |

# Signature page

| Title | A randomized, double-blind, parallel-group study to evaluate the safety and efficacy of switching from innovator infliximab to biosimilar infliximab compared with maintained treatment with innovator infliximab in patients with rheumatoid arthritis, spondyloarthritis, psoriatic arthritis, ulcerative colitis, Crohn’s disease and chronic plaque psoriasis: The NOR-SWITCH study |
| --- | --- |
| Protocol ID no: | NOR-SWITCH study |
| EudraCT no: | 2014-002056-40 |

***I hereby declare that I will conduct the study in compliance with the Protocol, ICH GCP and the applicable regulatory requirements:***

| **Name** | **Title** | **Role** | **Signature** | **Date** |
| --- | --- | --- | --- | --- |
| Kåre Birger Hagen | PT, PhD, Prof. | Sponsor |  | 19-05-2015 |
| Tore K. Kvien | MD, PhD, Prof. | Coordinating Investigator |  | 19-05-2015 |
| Inge Christoffer Olsen | MSc, PhD | Biostatistician |  | 19-05-2015 |
|  |  | Local Principal Investigators |  |  |

# PROTOCOL SYNOPSIS

| **Title of study** | A randomized, double-blind, parallel-group study to evaluate the safety and efficacy of switching from innovator infliximab to biosimilar infliximab compared with maintained treatment with innovator infliximab in patients with rheumatoid arthritis, spondyloarthritis, psoriatic arthritis, ulcerative colitis, Crohn’s disease and chronic plaque psoriasis: The NOR-SWITCH study |
| --- | --- |
| **Short title** | The NOR-SWITCH study |
| **Control treatment** | Innovator infliximab (Remicade®): INX  INX is an immunoglobulin (IgG1 chimeric human-murine monoclonal antibody) TNF inhibitor used to treat autoimmune diseases. The dose of INX will be identical to the last dose of INX before randomization. |
| **Investigational Medical Product** | Biosimilar infliximab (Remsima®): CT-P13  CT-P13 is an immunoglobulin (IgG1 chimeric human-murine monoclonal antibody) TNF inhibitor, bio-similar to innovator infliximab. The dose of CT-P13 will be identical to the last dose of INX before randomization |
| **EudraCT No.** | 2014-002056-40 |
| **Phase of development** | Phase IV |
| **Study Centers Planned** | This is a national, multi-centre study with approximately 35 study centres planned in all health regions in Norway |
| **Study Period** | Estimated date of first patient enrolled: October 1st 2014  Anticipated recruitment period: October 1st 2014 - July 15th 2015  Estimated date of last patient completed: January 15th 2016 |
| **Treatment Duration** | 52 weeks with a 26-week follow-up period |
| **Study objectives** | Primary:  To assess if CT-P13 is non-inferior to innovator infliximab (INX) with regard to disease worsening in patients who have been on stable INX treatment for at least 6 months  Secondary:   - To assess the safety and immunogenicity of CT-P13 compared to INX in patients who have been on stable INX treatment for at least 6 months - To compare the efficacy of CT-P13 to INX in patients who have been on stable INX treatment for at least 6 months applying generic and disease-specific outcome measures   Exploratory:   - To compare cost-effectiveness of CT-P13 to INX in patients who have been on stable INX treatment for at least 6 months - To assess the safety and efficacy of switching from INX to CT-P13 compared to continued treatment with CT-P13 during a 26-week follow-up period after completing the 52-week randomized period of NOR-SWITCH |
| **Study Endpoints** | Primary endpoint:  Occurrence of disease worsening during the 52-week study period based on disease specific efficacy assessment scores  Secondary endpoints:  Generic:   - Time from randomization to disease worsening - Patient and Physician Global assessment of disease activity - Occurrence of drug discontinuation - Time from randomization to drug discontinuation   Disease-specific:   - Inflammation assessed by biochemical parameters - RA and PsA: DAS28, MHAQ, RAID (for RA), PsAID (for PsA) - SpA: ASDAS, MHAQ, BASDAI - UC: Partial Mayo score, IBDQ - CD: HBI, IBDQ - Chronic plaque psoriasis: PASI, DLQI   Exploratory endpoints:   - EQ-5D - SF-36 - WPAI-GH - Use of health care resources   The same endpoints will be used in the 26-week follow-up period. |
| **Study Design and Description** | A randomized, double-blind, controlled, parallel-group, multicenter, phase IV, non-inferiority comparative study |
| **Study population** | Norwegian adult male and female patients with a clinical diagnosis of either rheumatoid arthritis, spondyloarthritis, psoriatic arthritis, ulcerative colitis, Crohn’s disease or chronic plaque psoriasis receiving stable treatment with innovator infliximab during the last 6 months |
| **Main Inclusion Criteria** | 1. A clinical diagnosis of either rheumatoid arthritis, spondyloarthritis, psoriatic arthritis, ulcerative colitis, Crohn’s disease or chronic plaque psoriasis 2. Male or non-pregnant, non-nursing female 3. >18 years of age at screening 4. Stable treatment with innovator infliximab (Remicade®) during the last 6 months 5. Subject capable of understanding and signing an informed consent form 6. Provision of written informed consent |
| **Main Exclusion Criteria** | 1. Major co-morbidities, such as severe malignancies, severe diabetes mellitus, severe infections, uncontrollable hypertension, severe cardiovascular disease (NYHA class 3 or 4), severe respiratory diseases and/or other diseases including inflammatory conditions for which infliximab is contra-indicated 2. Change of major co-medication during the last 2 months prior to randomization:   RA, SpA and PsA: Initiation of systemic corticosteroids or synthetic DMARDs or other medication which according to the investigator would interfere with the stability of the disease.  UC and CD: Initiation of systemic corticosteroids or an immunosuppressant or other medication which according to the investigator would interfere with the stability of the disease  Chronic plaque psoriasis: Initiation of methotrexate, cyclosporin or other medication which according to the investigator would interfere with the stability of the disease   1. Inadequate birth control, pregnancy, and/or breastfeeding. Adequate contraception includes oral, injected or implanted hormonal methods of contraception, placement of an intrauterine device or system, vasectomized partner or sexual abstinence. 2. Psychiatric or mental disorders, alcohol abuse or other substance abuse, language barriers or other factors which makes adherence to the study protocol impossible 3. Change in treatment with innovator infliximab (Remicade®) during the last 6 months due to disease related factors, not including dose/frequency adjustments due to drug concentration measurements 4. For patients with UC and CD: Functional colostomy or ileostomy. Extensive colonic resection with less than 25 cm of the colon left in situ. |
| **Sample Size** | Five-hundred (500) patients are planned to be recruited. The sample size is based on the following assessment:  If there is truly no difference between INX and CT-P13 treatment on the proportion of patients with disease worsening after 52 weeks, 394 patients (197 in each arm) are required to be 90% sure that the upper limit of a one-sided 97.5% confidence interval (or equivalently a two-sided 95% confidence interval) will exclude a difference in favor of INX of more than 15%. In order to reach 394 patients in the primary per-protocol population we need to randomize 492 patients to compensate for an estimated 20% protocol violators |
| **Randomization Procedure** | Eligible patients will be allocated in a 1:1 ratio between INX and CT-P13 treatment, using a computer randomization procedure stratified by clinical diagnosis. Randomization will be performed electronically through the eCRF (Viedoc). Patients entering the 26-week follow-up period will all be treated with open-label CT-P13 |
| **Efficacy Assessments** | Efficacy variables include occurrence of disease worsening, composite disease activity scores, visual analogue scales, utility measures, inflammation assessed by biochemical parameters, health related quality of life, work participation |
| **Safety Assessments** | Physical examination and vital signs, laboratory tests, record of adverse events and serious adverse events, serum drug concentrations and immunogenicity |
| **Other Assessments** | Resource utilization and cost-effectiveness/cost-utility assessments |
| **Statistical methods and data analysis** | The main analysis is planned when all patients have concluded the study, all data have been entered, verified and validated and the database has been locked.  The primary variable will be analyzed using a logistic regression model with treatment group, stratification factors and treatment duration of INX at baseline as explanatory variables. The primary analysis will be performed on the per-protocol population. The null hypothesis to be tested is that the difference in proportion of patients experiencing disease worsening is at least 15% in favor of INX compared to CT-P13. The primary variable will be evaluated by the 95% confidence limits. A conclusion of non-inferiority will be made if the 95% confidence limits of the estimated treatment difference fully lie within the non-inferiority margin of 15%.  For the 26-week follow-up period we will compare the patients switching from INX to CT-P13 with the patients continuing on CT-P13. |

# TABLE OF CONTENTS

CONTACT details 2

Signature page 4

PROTOCOL SYNOPSIS 5

TABLE OF CONTENTS 9

List of Abbreviations and Definitions of Terms 13

1 introduction 15

1.1 Background – Diseases 15

1.1.1 Rheumatic diseases 15

1.1.2 Inflammatory bowel diseases 15

1.1.3 Chronic plaque psoriasis 16

1.2 Background - Therapeutic Information 16

1.3 Pre-Clinical & Clinical Experience with Investigational Medicinal Product (IMP) 17

1.4 Rationale for the Study and Purpose 17

2 STUDY OBJECTIVES and related endpoints 19

3 Overall STUDY Design 20

4 STUDY POPULATION 20

4.1 Selection of Study Population 20

4.2 Number of Patients 20

4.3 Inclusion Criteria 20

4.4 Exclusion Criteria 21

4.5 Specification of stable treatment 21

5 TREATMENT 22

5.1 Drug Identity, Supply and Storage 22

5.2 Dosage and Drug Administration 22

5.3 Duration of Therapy 22

5.4 Premedication and Monitoring 22

5.5 Schedule Modifications 22

5.6 Concomitant Medication 22

5.7 Subject Compliance 22

5.8 Drug Accountability 22

5.9 Drug Labeling 23

5.10 Subject Numbering 23

6 STUDY procedures 23

6.1 Flow Chart 23

6.2 By Visit 23

6.2.1 Screening evaluation 23

6.2.2 Baseline visit 24

6.2.3 Regular visit 24

6.2.4 Non-scheduled visit in case of disease worsening 24

6.2.5 End of Study Visit 25

6.2.6 Withdrawal Visit 25

6.2.7 After End of Treatment (Follow-up) 25

6.3 Criteria for Patient Discontinuation 25

6.4 Procedures for Discontinuation 25

6.4.1 Patient Discontinuation 25

6.4.2 Treatment discontinuation 25

6.4.3 Trial Discontinuation 25

6.5 Laboratory Tests 26

6.6 Linkage to other registers 26

7 assessments 26

7.1 Efficacy Assessments 26

7.1.1 General efficacy assessments: 26

7.1.2 Disease specific efficacy assessments: RA, PsA 26

7.1.3 Disease specific efficacy assessments: SpA 27

7.1.4 Disease specific efficacy assessments: Ulcerative colitis 28

7.1.5 Disease specific efficacy assessments: Crohn’s disease 28

7.1.6 Disease specific efficacy assessments: Psoriasis 28

7.2 Assessment of Disease Worsening 28

7.2.1 Disease worsening in RA and PsA 28

7.2.2 Disease worsening in SpA 28

7.2.3 Disease worsening in ulcerative colitis 28

7.2.4 Disease worsening in Crohn’s disease 28

7.2.5 Disease worsening in psoriasis 28

7.2.6 Patient and investigator consensus on disease worsening 29

7.3 Immunogenicity and Serum Drug Concentration Assessments 29

7.4 Biobank samples 29

7.5 Safety and Tolerability Assessments 29

7.5.1 Physical examination 29

7.5.2 Vital signs 29

7.5.3 Laboratory assessments 29

7.6 Other Assessments 30

8 Safety MONItoring and reporting 31

8.1 Definitions 31

8.1.1 Adverse Event (AE) 31

8.1.2 Serious Adverse Event (SAE) 32

8.1.3 Suspected Unexpected Serious Adverse Reaction (SUSAR) 32

8.2 Expected Adverse Events 32

8.3 Disease Progression/Recurrence 32

8.4 Time Period for Reporting AE and SAE 32

8.5 Recording of Adverse Events 33

8.6 Reporting Procedure 33

8.6.1 AEs and SAEs 33

8.6.2 SUSARs 34

8.6.3 Annual Safety Report 34

8.6.4 Clinical Study Report 34

8.7 Procedures in Case of Emergency 34

9 Data management and monitoring 34

9.1 Electronic Case Report Forms (CRFs) 34

9.2 Source Data 35

9.3 Study Monitoring 35

9.4 Confidentiality 36

9.5 Database management 36

10 Statistical methods and data analysis 36

10.1 Determination of Sample Size 36

10.2 Randomization 37

10.2.1 Allocation- sequence generation 37

10.2.2 Allocation- procedure to randomize a patient 37

10.2.3 Blinding and emergency unblinding 37

10.3 Population for Analysis 38

10.4 Planned analyses 38

10.5 Statistical Analysis 39

10.5.1 Statistical model 39

10.5.2 Primary variable 39

10.5.3 Secondary/exploratory variables 40

10.5.4 Health economic analyses 41

10.5.5 Missing data 41

11 STUDY MANAGEMENT 41

11.1 Investigator Delegation Procedure 41

11.2 Protocol Adherence 41

11.3 Study Amendments 42

11.4 Audit and Inspections 42

12 Ethical and regulatory requirements 42

12.1 Ethics Committee Approval 42

12.2 Other Regulatory Approvals 42

12.3 Informed Consent Procedure 42

12.4 Subject Identification 42

13 Trial sponsorship and financing 43

14 Trial insurance 43

15 Publication policy 43

16 REFERENCES 43

17 APPENDICES 48

17.1 Trial flow chart 48

17.2 RAID questionnaire 49

17.3 PsAID Questionnaire 50

17.4 BASDAI questionnaire 52

17.5 Partial Mayo Score 53

17.6 Harvey-Bradshaw Index 54

17.7 MHAQ 55

17.8 IBDQ 56

17.9 DLQI 65

17.10 SF-36 67

17.11 EQ-5D 71

17.12 WPAI:GH 72

# List of Abbreviations and Definitions of Terms

| **Abbreviation or special term** | **Explanation** |
| --- | --- |
| ACR | American College of Rheumatology |
| AE | Adverse Event |
| ALP | Alkaline phosphatase |
| ALT | Alanine aminotransferase |
| AS | Ankylosing spondylitis |
| ASA | Aminosalicylate |
| ASAS | Assessment of SpondyloArthritis International Society |
| ASDAS | Ankylosing Spondylitis Disease Activity Score |
| AST | Aspartate transaminase |
| AZA | Azathioprine |
| BASDAI | Bath Ankylosing Spondylitis Disease Activity Index |
| bDMARD | Biological Disease-Modifying Anti-Rheumatic Drugs |
| CD | Crohn’s disease |
| CIOMS | Council for International Organizations of Medical Sciences |
| CRF | Case Report Form (electronic/paper) |
| CRP | C-reactive protein |
| CSA | Clinical Study Agreement |
| CTC | Common Toxicity Criteria |
| CTCAE | Common Terminology Criteria for Adverse Event |
| CTCAE | Common Terminology Criteria for Adverse Events version |
| CT-P13 | Biosimilar infliximab |
| DAE | Discontinuation due to Adverse Event |
| DAS28 | Disease Activity Score using 28 joints |
| DLQI | Dermatology Life Quality Index |
| DMARD | Disease-Modifying Anti-Rheumatic Drugs |
| DRG | Diagnosis related group |
| e-CRF | electronic Case Report Form |
| EMA | European medicines agency |
| EPJ | Electronic patient journal |
| ESR | Erythrocyte Sedimentation Rate |
| EULAR | European League Against Rheumatism |
| GCP | Good Clinical Practice |
| GI | Gastrointestinal |
| HBI | Harvey-Bradshaw Index |
| HRQOL | Health related quality of life |
| IB | Investigator’s Brochure |
| IBD | Inflammatory bowel diseases |
| IBDQ | Inflammatory Bowel Disease Questionnaire |
| ICF | Informed Consent Form |
| ICH | International Conference on Harmonization |
| IgG | Immunoglobulin G |
| IJD | Inflammatory Joint Diseases |
| IL | Interleukin |
| IMP | Investigational Medicinal Product (includes active comparator and placebo) |
| IND | Investigational New Drug |
| INF | Interferon |
| INX | Innovator infliximab |
| ISF | Investigator Site Files |
| MHAQ | Modified Health Assessment Questionnaire |
| MP | Mercaptopurine |
| MRI | Magnetic resonance imaging |
| NK | Natural killer |
| NorCRIN | Norwegian clinical research infrastructure network |
| NSAID | Non-steroidal anti-inflammatory drug |
| PASI | Psoriasis Area and Severity Index |
| PGA | Patient Global Assessment of Disease Activity |
| PhGA | Physician Global Assessment of Disease Activity |
| PRO | Patient reported outcome |
| PsA | Psoriatic arthritis |
| PsAID | Psoriatic Arthritis Impact of Disease |
| PUVA | Photochemotherapy |
| QALY | Quality-adjusted life year |
| RA | Rheumatoid arthritis |
| RAID | Rheumatoid Arthritis Impact of Disease |
| SAE | Serious Adverse Event |
| SD | Stable Disease |
| sDMARD | Synthetic Disease-Modifying Anti-Rheumatic Drugs |
| SDV | Source data verification |
| SF-36 | Short Form (36) Health Survey |
| SOP | Standard Operating Procedure |
| SpA | Spondyloarthritis |
| SmPC | Summary of Product Characteristics |
| SUSAR | Suspected Unexpected Serious Adverse Reaction |
| TMF | Trial master file |
| TNF | Tumor necrosis factor |
| TNFi | TNF inhibitor |
| UC | Ulcerative colitis |
| UVB | Ultraviolet B |
| WPAI:GH | Work Productivity and Activity Impairment Questionnaire: General Health |

#

# introduction

## Background – Diseases

The study will focus on three different rheumatic diseases, two inflammatory bowel diseases and chronic plaque psoriasis.

### Rheumatic diseases

Rheumatoid arthritis

Rheumatoid arthritis (RA) is the most frequent inflammatory rheumatic joint disease with a prevalence of about 0.5%[1] and an incidence of about 25 per 100 000.[2] The disease is approximately three times more common in females than males. It is characterized by inflammation of the synovial membrane, which may lead to functional disability, pain, reduced health related quality of life and damage of cartilage and bone. The overarching goal is to suppress inflammation to a level which will prevent development of disability and structural damage. The disease is classified according to the ACR/EULAR 2010 classification criteria.[3] A subset of patients with established disease and suppressed inflammation will not necessarily fulfill these classification criteria, but may be classified as RA if they fulfill the recently published definition of erosive disease.[4] Patients with RA may in addition to joint inflammation also have general disease manifestations like weight loss, fatigue and also suffer from extra-articular manifestations including increased risk of cardiovascular morbidity.

Ankylosing spondylitis

Ankylosing spondylitis (AS) is characterized by inflammation of the sacroiliac joints and to a variable extent also spinal inflammation and presence of radiographic abnormalities of the sacroiliac joint(s). It is more prevalent among males than females and the overall prevalence is around 0.2%.[5,6] Over time patients may develop erosive disease of the sacroiliac joints and bone formation in the spine which may lead to ankylosis. The typical symptoms are inflammatory back pain, which may be relieved by exercise and activity. The patients may in addition to the spinal manifestations have enthesitis, dactylitis, peripheral arthritis and uveitis. The diagnosis of non-radiographic spondyloarthritis (SpA) can also be established without radiographic abnormalities of the sacroiliac joints according to the 2009 ASAS criteria.[7] In Norway it is common practice to examine the patients with MRI and if MRI is positive the patient may be given a diagnosis of M45 or M46.8 and be treated according to the recommendations for AS, even if radiographic sacroiliitis has not been formally demonstrated.

Psoriatic arthritis

Psoriatic arthritis (PsA) is an inflammatory joint and/or spinal disease. The musculoskeletal manifestations accompany the skin disease psoriasis. The disease is very heterogeneous in its presentation and can clinically have many similarities to RA or to SpA. Sometimes PsA is classified as part of the SpA. PsA is usually diagnosed according to the CASPAR criteria[8] and has a prevalence of about 0.2%[6] It is equally distributed between male and females. The disease course is usually progressive leading to increasing joint deformities and disability.

### Inflammatory bowel diseases

Inflammatory bowel diseases (IBD) including ulcerative colitis (UC) and Crohn’s disease (CD) are chronic disorders involving mainly the gastrointestinal tract and characterized by chronic intestinal inflammation with huge impact on quality of life. UC involves the colon only, while in CD any part of the gastrointestinal tract may be affected. Environmental factors, gut dysbiosis, genetic susceptibility and an inappropriate immune response seem to be important contributors to the pathogenesis of these multifactorial diseases.[9] The incidence of IBD is particularly high in Norway and occurs mostly at a young age. Clinical manifestations of both CD and UC include diarrhea (typically bloody in patients with UC), as well as abdominal discomfort and pain, fecal urgency, and incontinence. Systemic features such as fever, weight loss, malaise, and fatigue are indicators of more severe disease. Extraintestinal manifestations such as uveitis, arthritis, AS, or primary sclerosing cholangitis may also be seen in conjunction with IBD. The diagnosis of UC or CD is usually made by histopathologic examination of endoscopic mucosal biopsy specimens obtained on ileocolonoscopy.

### Chronic plaque psoriasis

Psoriasis is a chronic, inflammatory, immune-mediated, systemic disease associated with high morbidity and increased mortality.[10,11] Prevalence varies worldwide from 0.9% (USA) to 11.4% (Norway).[12,13] The natural history of psoriasis is chronic with intermittent remissions and exacerbations, as well as periods of stable disease activity. Chronic plaque psoriasis is the most common subtype (90%), and is characterized by sharply demarcated plaques with erythema and silvery white scale.

It is estimated that 17-25% suffer from moderate to severe disease.[14] Between 6 and 42 % will develop psoriatic arthritis.[15] Severe psoriasis is associated with an increased risk to develop metabolic dysfunction and cardiovascular morbidity.[16] There are multiple subtypes of psoriasis. The diagnosis is clinical; histological confirmation is not necessary. The physical and psychological burden can, depending on the severity, cause quality of life impairment similar to other chronic conditions like cancer and heart disease.[17] Psoriasis has been associated with long-term work disability, loss of productivity and work absenteeism and burden on the health care systems.[18]

The pathogenesis of psoriasis is incompletely understood. The main pathophysiological characteristics of psoriasis are hyperproliferation of keratinocytes, abnormal adaptive and innate immune system reactions, and angiogenesis.[10,19] Genetic susceptibility with genes affecting antigen presentation and processing, interleukin 12/INF-gamma and IL 23/17, TNF-α/NK κB signalling, dendritic cell/macrophage function, epidermal barrier, antimicrobial defense and other risk factors are all involved in the pathogenesis. The nervous system, the vascular system, environmental factors, and infective agents are also believed to be involved in the keratinocyte dysfunction of psoriatic skin. Various factors (e.g., trauma, endocrine factors, psychosocial stress, infections or medications) may trigger psoriasis in genetically predisposed individuals. Epigenetic mechanisms may be a link between genetic and environmental factors.

## Background - Therapeutic Information

Patients with RA are typically treated with synthetic disease-modifying antirheumatic drugs (sDMARDs). If a predefined treatment target is not achieved, the patients will often receive addition of a biological DMARD (bDMARD), most commonly a TNF inhibitor (TNFi). If the target is still not achieved, switching to another TNFi or switching to another bDMARD with a different mode of action may be an opportunity. EULAR has recently updated the recommendations for drug management of RA.[20]

AS and axial SpA are also treated with bDMARDs. However, use of non-steroidal anti-inflammatory drugs (NSAIDs) has a more prominent role and bDMARDs should not be started unless the patient has failed at least two different NSAIDs. Among bDMARDs only TNFi have been approved for use in AS. ASAS and EULAR have published recommendations for management of AS[21] and ASAS has published recommendations for use of TNFi in SpA.[4] For AS five different TNFi have been approved and for non-radiographic axial SpA only certolizumab and adalimumab are formally approved. However, due to the similar efficacy of different TNFi it can be expected that some patients also with non-radiographic axial SpA are currently treated with other TNFi than certolizumab and adalimumab

The treatment strategy for PsA follows some of the same principles as for RA and AS/SpA. Patients will usually be treated with a sDMARD as the initial disease modifying therapy and will then receive a bDMARD if control of inflammation is not achieved.[22] Five different TNF inhibitors are approved for use in PsA and ustekinumab with inhibition of IL12-23 has also recently been approved for this indication.[23]

5-Aminosalicylates (5-ASAs) are the mainstay of UC pharmacotherapy for induction and maintenance of remission for patients with mild to moderate disease, but the effect of 5-ASAs in CD is questionable.[24,25] Corticosteroids are highly effective for induction of remission in both CD and UC, but not useful as maintenance therapy in either disease and carry significant undesirable side effects, including osteoporosis and increased risk of infection.

The thiopurines 6-mercaptopurine (6-MP) and azathioprine (AZA) are immunosuppressive agents which have a role in maintenance of remission in moderate to severe UC and CD, but their relatively slow onset of action precludes their use during flares of disease. Methotrexate has a role in the management of refractory CD especially in patients intolerant to thiopurines. Although used in the treatment of UC patients, the effect of methotrexate in this disease is still unresolved.[26]

TNFi such as infliximab (Remicade®), adalimumab (Humira®) and golimumab (Simponi®), have been studied and have proven useful for both induction and maintenance of remission in IBD.[27-30] In Norway infliximab and adalimumab are permitted to use in the treatment of moderate to severe UC and CD and golimumab only in the treatment of moderate to severe UC. In addition, the patients must have failed treatment with corticosteroids and/or immunosuppressants or shown to be intolerant to these agents. Although very effective for both induction and maintenance of remission in many IBD patients, treatment with TNFi has been associated with a number of serious adverse events (SAEs) involving hypersensitivity and infection. During maintenance therapy with these biological agents several patients loose their clinical response due to immunogenicity and formation of antibodies against the drug.[31] In some of the patients this problem can be solved by increasing the dose and/or shorten the interval between the drug administration.

Failure of medical therapy leads to colectomy in approximately 10% of patients with UC within 10 years.[32] Colectomy is considered to be an important adjunct treatment for refractory UC. However, colectomy with ileal pouch anal anastomosis is associated with complications, including high stool frequency, female infertility, and pouchitis.[33] Surgical removal of highly diseased or stenotic segments of bowel in CD is not curative. Clinical relapse occurs in a majority of patients with CD who undergo segmental resections, and the need for a second operation after some years is often seen.[34]

The chronicity of psoriasis requires long-term treatments that are effective and safe.[35] Patients have to cope with their psoriasis as well as the treatments for prolonged periods of time. Many patients are dissatisfied with their treatment.[36] For patients with mild disease topical agents (i.e., emollients, corticosteroids, vitamin-D or -A analogues, coal tar, dithranol) and phototherapy (broad-/narrowband UVB) are appropriate treatments.[37,38] Systemic therapies (i.e., methotrexate, cyclosporine, systemic retinoids) and photochemotherapy (PUVA) are recommended options for patients with more severe disease.[38-42] Many patients with moderate to severe plaque psoriasis receive less than optimal treatment.[36] Advancements in understanding the pathogenesis of psoriasis have been crucial to the development of biological therapies. These target the immune system and are suitable options for patients with extensive disease. Biological therapies for the treatment of psoriasis include anti-cytokine therapies (anti-tumour necrosis factor (TNF) therapies (adalimumab, etanercept, and infliximab) and a monoclonal antibody against interleukin (IL)-12 and IL-23 (ustekinumab). Biological therapies have been proven to suppress inflammation in plaque-type psoriasis and provide a rapid, effective and safe disease control in moderate-to-severe psoriasis.[43,44]

## Pre-Clinical & Clinical Experience with Investigational Medicinal Product (IMP)

A biosimilar drug is a bio-therapeutic product, which is similar in forms of quality, safety and efficacy to an already licensed reference bio-therapeutic product. Similarity is defined as the absence of relevant difference in the parameter of interest. CT-P13 is an immunoglobulin (IgG1 chimeric human-murine monoclonal antibody) bio-similar to originator infliximab. CT-P13 is produced in the same type of saline and has an identical amino acid sequence to infliximab. The drugs have been demonstrated to have comparable in vitro primary pharmacodynamics in a range of studies, comparable binding affinities to monomeric and trimeric forms of humane TNF-alpha, and comparable neutralising activity against the TNF-alpha sensitive mouse sarcoma cell line.[45,46]

Bio-similar infliximab was developed and tested by the South Korean company Celltrion who filed for EMA approval of the bio-similar infliximab product, CT-P13. European medicines agency (EMA) approved CT-P13 for RA, AS, PsA, IBD and chronic plaque psoriasis, ie. the indications in this study, in August 2013. Prior to this approval CT-P13 had undergone a stringent review process by EMA including examination of the amino acid sequence, animal studies and also clinical activity. One study in patients with RA had been performed (PLANETRA) comparing the biosimilar and innovator infliximab with regard to equivalence both for safety, efficacy and immunogenicity.[47] The equivalence between Remicade® and CT-P13 was demonstrated for all endpoints. A pharmacokinetic study performed in patients with AS (PLANETAS) also showed comparability between the innovator and biosimilar product.[48] Based on this information EMA approved CT-P13 for use not only in RA and AS, but also in PsA, IBD and chronic plaque psoriasis..

## Rationale for the Study and Purpose

This study will focus on switching from innovator infliximab to CT-P13, which is approved in Norway as Remsima® (Orion Pharma) and Inflectra® (Hospira). The rationale for the study is driven by financial motivation. Remsima® is currently 39% cheaper than Remicade® (Inflectra 33% cheaper). Thus, switching from Remicade® to biosimilar infliximab in patients on stable treatment will lead to considerable cost savings. However, such switching or automatic substitution is not part of the EMA approval and it is a general agreement that more data documenting that switching between biosimilar infliximabs is safe and maintains efficacy, are needed before automatic substitution should be applied. Follow-up studies from the PLANETRA[47] and PLANETAS[48] studies after one year have included switching of patients from originator infliximab to CT-P13 and no definite unfavourable safety signal or reduced efficacy have been observed in the extensions of the original trials. However, these studies have not yet been published as journal articles and some concerns have been raised during the last year about a safety signal in the extension of the PLANETAS study (reported adverse events during the second year by 48.9% of the patients continuing with CT-P13 versus 71.4% of the patients switching from INX to CT-P13). This observation and the fact that the extension studies have not been published has motivated us to take the opportunity to provide additional switch data.

Except for an observational pilot study with preliminary clinical data from a very small cohort of 23 IBD patients, no studies have previously been conducted with CT-P13 in ulcerative colitis, Crohn’s disease and chronic plaque psoriasis. However, the EMA approval applies all indications as for the originator infliximab (Remicade®). In this study all these patients groups will participate.

Thus, it is a need to provide more switch data to see if CT-P13 is non-inferior to the innovator infliximab (Remicade®) regarding efficacy, safety and immunogenicity. The treatment regimen after randomisation will be unchanged regarding dose and interval between infusions. The dose may vary according to indication (e.g. the standard dose for AS and IBD is 5 mg/kg whereas 3 mg/kg in RA).

# STUDY OBJECTIVES and related endpoints

CT-P13: Biosimilar infliximab (Remsima®); INX: Innovator infliximab (Remicade®)

|  | **Objectives** | **Endpoints** | **Assessments** |
| --- | --- | --- | --- |
| Primary | To assess if CT-P13 is non-inferior to innovator infliximab (INX) with regard to disease worsening in patients who have been on stable INX treatment for at least 6 months | Primary efficacy endpoint:   - Occurrence of disease worsening during the 52-week study period based on disease specific efficacy assessment scores | Section 7.2 |
| Secondary | To assess the safety and immunogenicity of CT-P13 compared to INX in patients who have been on stable INX treatment for at least 6 months | Safety endpoints   - Adverse events frequency - Serum drug concentrations - Immunogenicity parameters - Vital signs | Section 7.5 |
|  | To compare efficacy of CT-P13 to INX in patients who have been on stable INX treatment for at least 6 months | Secondary endpoints  Secondary efficacy endpoint   - Time from randomization to disease worsening   Generic:   - Patient and Physician Global assessment of disease activity - Occurrence of drug discontinuation - Time from randomization to drug discontinuation   Disease-specific:   - Inflammation assessed by biochemical parameters - RA and PsA: DAS28, MHAQ, RAID (for RA), PsAID (for PsA) - SpA: ASDAS, MHAQ, BASDAI - UC: Partial Mayo score, IBDQ - CD: HBI, IBDQ - Chronic plaque psoriasis: PASI, DLQI | Section 7.1 |
| Exploratory | To compare cost-effectiveness of CT-P13 to INX in patients who have been on stable INX treatment for at least 6 months | Exploratory endpoints   - EQ-5D - SF-36 - WPAI-GH - Use of health care resources | Section 7.6 |
|  | To assess the safety and efficacy of switching from INX to CT-P13 compared to continued treatment with CT-P13 during a 26-week follow-up period after completing the 52-week randomized period of NOR-SWITCH | Exploratory endpoints   - Same as in the main study period |  |

# Overall STUDY Design

The NOR-SWITCH study is designed as a randomized, double blind, controlled, parallel-group, multi-center, single-country, non-inferiority comparative phase IV study.

Any patient with a diagnosis of rheumatoid arthritis, psoriatic arthritis, spondyloarthritis, inflammatory bowel disease or chronic plaque psoriasis on stable treatment with INX for at least 6 months are potential study patients. Eligibility criteria are described in section 4.3 (inclusion criteria) and 4.4 (exclusion criteria). Eligible patients with informed consent will be randomized 1:1 according to the procedure described in section 10.2.2 to either continued INX treatment or CT-P13 treatment. Infliximab treatment regimen will be kept unchanged from the pre-randomization treatment regimen. Randomized treatment will be continued for the duration of the randomized trial period (52 weeks) with visits according to pre-randomization treatment regimen injection interval. If serum concentration measurements suggest dose adjustments of the INX treatment, the patient should receive the adjusted dose for at least one treatment period before inclusion in the NOR-SWITCH study.

Eligible and consenting patients still on randomized treatment after 52 weeks will be continued in a 26-week follow-up period with open-label CT-P13 treatment.

In order to identify the primary endpoint (occurrence of disease worsening), each study center will have a phone number for patients to call in case of increasing disease activity. If a patient is experiencing a potential disease flare, a visit will be arranged within one week to allow for a thorough examination and documentation of disease status.

| Study Period | Estimated date of first patient enrolled: October 1^st^ 2014  Anticipated recruitment period: October 1^st^ 2014 to July 15^th^ 2015  Estimated date of last patient completed randomized study period: July 15^th^ 2016  Estimated date of last patient completed open-label study period: January 15^th^ 2017 |
| --- | --- |
| Treatment Duration: | 52 weeks |
| Follow-up: | 26 weeks with open-label CT-P13 treatment |

A schematic of the study design is shown below:

Figure 1 Study Design


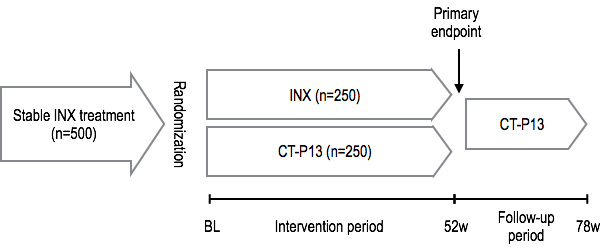


# STUDY POPULATION

## Selection of Study Population

Norwegian adult male and female patients with a clinical diagnosis of either rheumatoid arthritis, spondyloarthritis, psoriatic arthritis, ulcerative colitis, Crohn’s disease or chronic plaque psoriasis on stable treatment with INX during the last 6 months. Patients will potentially be recruited from all Norwegian hospitals providing treatment with INX for the mentioned diagnoses.

## Number of Patients

Five-hundred (500) patients are planned to be included in this trial.

## Inclusion Criteria

All of the following conditions must apply to the prospective patient at screening prior to receiving study agent (e.g.):

1. A clinical diagnosis of either rheumatoid arthritis, spondyloarthritis, psoriatic arthritis, ulcerative colitis, Crohn’s disease or chronic plaque psoriasis
2. Male or non-pregnant, non-nursing female
3. >18 years of age at screening
4. Stable treatment of innovator infliximab (Remicade®) during the last 6 months
5. Subject capable of understanding and signing an informed consent form
6. Provision of written informed consent

## Exclusion Criteria

Patients will be excluded from the study if they meet any of the following criteria:

1. Major co-morbidities, such as severe malignancies, severe diabetes mellitus, severe infections, uncontrollable hypertension, severe cardiovascular disease (NYHA class 3 or 4), severe respiratory diseases and/or other diseases including inflammatory conditions for which infliximab is contra-indicated.
2. Change of major co-medication during the last 2 months prior to randomization:

RA, SpA and PsA: Initiation of systemic corticosteroids or synthetic DMARDs or other medication which according to the investigator would interfere with the stability of the disease.

UC and CD: Initiation of systemic corticosteroids or an immunosuppressant or other medication which according to the investigator would interfere with the stability of the disease

Psoriasis: Initiation of methotrexate, cyclosporine or other medication which according to the investigator would interfere with the stability of the disease

1. Inadequate birth control, pregnancy, and/or breastfeeding. Adequate contraception includes oral, injected or implanted hormonal methods of contraception, placement of an intrauterine device or system, vasectomized partner or sexual abstinence.
2. Psychiatric or mental disorders, alcohol abuse or other substance abuse, language barriers or other factors which makes adherence to the study protocol impossible
3. Change in treatment with innovator infliximab (Remicade®) during the last 6 months due to disease related factors, not including dose/frequency adjustments due to drug concentration measurements
4. For patients with UC and CD: Functional colostomy or ileostomy. Extensive colonic resection with less than 25 cm of the colon left in situ.

## Specification of stable treatment

A stable treatment with INX is defined as

1. Unchanged dose and frequency, or
2. Adjustments of dose and/or frequencies which are based solely on drug concentration measurements

In the latter case, the patient should receive the adjusted dose for at least one treatment period before inclusion in the study.

Conditions for which the treatment with INX is regarded unstable includes (but are not limited to) treatment changes due to:

1. Clinical assessments of treatment efficacy
2. Adverse events or other safety issues

Minor changes in treatment interval according to clinical practice are acceptable.

## Inclusion Criteria for the 26-week Open-Label Follow-Up Period

In order to proceed into the open-label follow-up period, the patient must fulfill the following criteria:

1. Inclusion and randomization in the NOR-SWITCH study
2. Completion of the 52-week intervention period, and still on randomized treatment
3. Provision of written informed consent to the participate in the 26-week open-label follow-up period

Patients in the NOR-SWITCH study will be asked to participate in the open-label follow-up period if they received randomized treatment at the last visit of the intervention period.

# TREATMENT

For this study biosimilar infliximab (Remsima®) denoted as CT-P13 and innovator infliximab (Remicade®) denoted INX are defined as Investigational Medicinal Products (IMPs). The test treatment is CT-P13 and the comparator is INX. INX must have been given for at least 6 months prior to randomization.

After the 52-week randomized period, eligible and willing patients will enter an open-label follow-up period of 26 weeks with CT-P13 treatment.

## Drug Identity, Supply and Storage

The storage and preparation instructions for CT-P13 and INX will follow the package leaflet. The storage and administration will be performed according to local guiding in each participating centre. The IMP will be prepared for infusion by an unblinded nurse who is otherwise not involved in the treatment of the patient.

## Dosage and Drug Administration

The dose of the study medication will be identical to the last dose of INX before randomization. Both drugs will be administered as intravenous infusions. The study treatment will be administered to the patient by authorized personnel.

## Duration of Therapy

The patients will be treated in the intervention period for 52 +/- 4 weeks after randomization. The number of infusions will differ between different patients according to their dosing intervals before randomization. The last infusion in the intervention period should be performed between week 48 and week 56 after randomization. After the last infusion with randomized treatment, eligible and consenting patients will enter an open-label follow-up period of 26 +/- 4 weeks. The last infusion in the follow-up period should be performed between week 22 and 30 after completion of the intervention period.

## Premedication and Monitoring

The infusion time will be the same as before randomization, usually about one hour, but with some individual modifications which may be influenced by previous experience with for example infusion reactions. The enrolled patients will receive the same type and dosage of pre-medication (sometimes antihistamines) as they had prior to randomization. The patients will be monitored by a nurse in the infusion unit during the infusion and 30 minutes afterwards with regard to potential allergic reactions/infusion reactions.

## Schedule Modifications

Treatment will continue with the same dose and the same infusion intervals during both the 52-week intervention period and the 26-week follow-up period. However, changes in dose and interval may be permitted due to non-disease related factors such as intercurrent infections, surgery, vacation etc. at the discretion of the treating physician. Drug levels will be measured at each visit, but only be reported on request. Changes in dose/interval based on serum drug levels are not permitted.

## Concomitant Medication

Patients are allowed to continue with the same concomitant medication as prior to randomization. Such medication may include 5-ASAs, systemic corticosteroids, non-steroidal anti-inflammatory drugs (NSAIDs) and sDMARDs like methotrexate, azathioprine and 6-MP. All changes in concomitant medication should be documented. Worsening in disease leading to major changes in the concomitant treatment will lead to classification as worsening of disease (primary endpoint) . Other biological agents used to treat the same diseases are not permitted. Live attenuated vaccines are not recommended.

## Subject Compliance

Each treatment administration will be registered in the electronic case report form (eCRF) with dose and time of infusion, and if the infusion was successful.

## Drug Accountability

The responsible site personnel will treat study drug according to the practice at the study site, including accountability of receipt, administration to the patient, returned and/or destruction at the site.

The IMPs will be stored at the pharmacy according to the practice at the study site. After randomization, un-blinded authorized study personnel will order the IMP form the pharmacy in an unbroken original package and prepare the infusion solution.

## Drug Labeling

The IMPs will have standard labeling from the manufacturer including package insert until it is prepared by the un-blinded study personnel.

The IMPs (CT-P13, INX) will be ordered from the hospital pharmacy’s general drug supply according to the same procedures as for any patient who is given treatment with one of these drugs. The un-blinded nurse who prepares the infusion solution will record the batch number and expiry date from the original drug package on a separate list that is not available for the blinded study personnel. The list will contain successive so called mixing numbers (“Blandingsnummer”).  The batch number and expiry date will be recorded next to the mixing number which will be used for the respective infusion solution and recorded on the label used for the final infusion solution.

The infusion solution container will be labeled with the following information:

- The name of the Investigator
- Drug formulation, route of administration and dosing
- Study code; NOR-SWITCH study
- Unique patient identification number (see below) «For clinical investigation use only»
- Mixing number (see above)
- Date and time finishing mixing of the infusion
- “To be used no later than (date and time)”
- “If not used immediately after mixing, the solution may be stored for up to 24 hours in refrigerator (+2 – 8 ^o^C).”

The open-label treatment in the follow-up period will have standard labeling from the manufacturer including package insert.

## Subject Numbering

When the patient has signed the Informed Consent Form he/she will be randomized and assigned a unique patient identification number. Once assigned, this number cannot be reused for any other patient.

# STUDY procedures

The visits will be carried out according to the patient’s pre-randomization innovator infliximab treatment schedule. Innovator infliximab is currently infused with a treatment schedule of every 4 to 10 weeks. Over the 52±4 weeks study period the number of visits will be between 5 and 13.

Over the 26±4 weeks open-label follow-up period the number of visits will be between 3 and 6.

The assessments performed at visits are presented in section 7.

Prior to inclusion, assumed eligible patients will receive a letter with information about the study.

## Flow Chart

An event flow chart is presented in Appendix 17.1.

## By Visit

### Screening evaluation

All patients must sign and date the written informed consent form before any study specific assessments or procedures are performed. A screening assessment should be performed before the start of the study (or at the baseline visits for patients who fulfill the entry criteria). The following procedures have to be completed:

- A formal assessment of the eligibility criteria
- Urine sample for pregnancy test

### Baseline visit

Informed written consent must have been given voluntarily by each subject before any study specific procedures are initiated. In addition to the assessments and procedures performed at a regular visit, the following will be done:

1. Full blood samples for biobank will be drawn and stored in a freezer at -70° C
2. Study nurse/investigator assessments:
   - Demographics (sex, birth date and ethnic origin)
   - Clinical status (medical history, physical examination and vital signs)
   - Start of diagnosis, start of treatment with ongoing Remicade and start of first biologic treatment
   - Previous immunosuppressive treatment and surgery
   - Information on underlying disease
   - Education and smoking status
3. Review of inclusion/exclusion criteria
4. Treatment administration
   - Randomization
   - Pre-randomization treatment schedule to be followed in the study

### Regular visit

The sequence of assessments and procedures is to be standardized as follows:

1. Laboratory samples for hematology, clinical chemistry, urinalysis, fecal analysis (IBD), immunogenicity, drug concentration and biobank storage must be drawn/collected prior to the infusion, preferably on the same day and not more than 7 days before the infusion
2. Patient reported health outcomes assessments

- Patient Global Assessment of disease activity (NRS)
- EQ-5D
- SF-36
- WPAI-GH
- RA: MHAQ, RAID
- PsA: MHAQ, PsAID
- SpA: MHAQ, BASDAI
- UC and CD: IBDQ
- Chronic plaque psoriasis: DLQI

1. Study nurse/investigator assessments:

- Nurse/investigator global assessment of disease activity (NRS)
- Disease specific disease activity measures, preferably done by the same assessor for consistency
  - - RA and SpA: DAS28
    - SpA: ASDAS
    - UC: Partial Mayo score
    - CD: HBI
    - Psoriasis: PASI
- Assessment of disease worsening
- Registration of concomitant medication
- Safety assessments (AEs/SAEs, vital signs)

1. Treating physician:

- Review of laboratory results

1. Treatment administration

- Time and dose of injection

### Non-scheduled visit in case of disease worsening

If the patient suspects a disease worsening, he or she should contact the study site immediately and be seen there as soon as possible and within two weeks as the latest. The visit will include all assessments of a regular visit (with the exception of treatment administration).

### End of Study Visit

The end of study visit will be performed at or near week 52±4 and will include a formal end of study assignment in the eCRF in addition to all assessments of a regular visit.

### Withdrawal Visit

A withdrawal visit will include all assessments of a regular visit (with the exception of treatment administration) in addition to an assessment of reason for withdrawal, time of withdrawal and if the patient wishes to continue follow-up in the study.

### After End of Randomized Treatment (Follow-up)

Patients entering the open-label follow-up period will continue with study visits at the time of the therapeutic infusions and assessments will be the same as at a regular visit during the intervention period.

## Criteria for Patient Discontinuation

Patients may be discontinued from study at any time. Specific reasons for discontinuing a patient from this study are:

- Voluntary discontinuation by the patient who is at any time free to discontinue his/her participation in the study, without prejudice to further treatment
- Major protocol deviation
- Incorrect enrolment i.e., the patient does not meet the required inclusion/exclusion criteria for the study
- Patient lost to follow-up

## Procedures for Discontinuation

### Patient Discontinuation

Patients have the right to withdraw from the study at any time for any reason. In the case that a patient decides to prematurely withdraw from the study, he or she should be asked if they can still be contacted for further information, so that a final evaluation can be made with an explanation of why the patient is withdrawing from the study, including assessment of possible adverse events. Although a subject is not obliged to give his or her reason(s) for withdrawing prematurely from a trial, the investigator should make a reasonable effort to ascertain the reason(s), while fully respecting the subject's rights.

If possible, at the last visit of the patient, all assessments of the ”Withdrawal visit” will be done.

The investigator is obliged to follow up any significant AE/SAE until the outcome is either recovered or resolved, recovering or resolving, not recovered or not resolved, recovered or resolved with sequelae, fatal or unknown.

### Treatment discontinuation

Patients who for some reason (e.g. pregnancy, reactions to treatment, surgical interventions or other safety issues) withdraw from the study medication will be asked to continue follow-up in the study.

### Trial Discontinuation

The whole trial may be discontinued at the discretion of the PI or the sponsor in the event of any of the following:

- Occurrence of AEs unknown to date in respect of their nature, severity and duration
- Medical or ethical reasons affecting the continued performance of the trial
- Difficulties in the recruitment of patients

The sponsor and principal investigator will inform all investigators, the relevant Competent Authorities and Ethics Committees of the termination of the trial along with the reasons for such action. If the study is terminated early on grounds of safety, the Competent Authorities and Ethics Committees will be informed within 15 days.

## Laboratory Tests

Hematology, clinical chemistry, urine and fecal samples will be drawn and, depending on availability, analyzed at the local laboratory according to hospital procedures. If any required tests are not available locally, samples will be referred to other laboratories according to local practice. Serum samples for measurements of drug concentrations and anti-drug antibodies will be sent to a central laboratory for analysis and biobanking (see sections 7.3 and 7.4).

## Linkage to other registers

In addition to the variables collected in this study, patients will be asked to give consent to collection of data from registries such as The Norwegian Prescription Database, The Norwegian Health Economics Administration, and the Norwegian Patient Register. This will allow certain outcomes to potentially be obtained though linkage to national medical or public registers and databases to answer research questions related to safety and health economics. Examples of such outcomes are cancer and other serious adverse events, health care utilization, work participation and social benefits. The Norwegian Arthritis Registry (NorArthritis) and The Norwegian Qualtiy Registry for Biologic Drugs (NOKBIL), The Cancer Registry of Norway (Kreftregisteret), the Norwegian Patient Registry (Norsk pasientregister – NPR), the Cause of Death Registry (Dødsårsaksregisteret), the Norwegian Prescription Database (Reseptregisteret), the Norwegian Myocardial Infarction Register (Norsk hjerteinfarktregister), the Norwegian Surveillance System for Communicable Diseases (Meldingssystem for smittsomme sykdommer – MSIS) and The Norwegian Labour and Welfare Administration (NAV) are potential data sources. NOR-DMARD is also a potential data source for patients who have previously been enrolled in the NOR-DMARD study. The patient consent form includes information about linkage.

Participation in international collaboration involving sharing of data from the NOR-SWITCH study and merging of NOR-SWITCH data with other (similar) studies will be based on fully de-identified data.

# assessments

## Efficacy Assessments

### General efficacy assessments:

Patient Global Assessment of Disease Activity (PGA)

PGA is measured on a numeric rating scale (NRS) 0-10 (0=none, 10=very severe) according to the question: “How active was your disease on average during the last week?”

Physician Global Assessment of Disease Activity (PhGA)

PhGA is measured on a numeric rating scale (NRS) 0-10 (0=none, 10=very severe) according to the question: “Please rate the patient’s overall (global) disease activity.”

Inflammation assessment by biochemical parameters

Inflammation is measured by C-reactive protein (CRP), the Erythrocyte Sedimentation Rate (ESR) for the inflammatory joint diseases, fecal calprotectin for the inflammatory bowel diseases according to hospital/laboratory standard procedures.

Drug discontinuation

If randomized treatment (INX or CT-P13) is permanently discontinued during the 52-week treatment period, the discontinuation date will be registered.

### Disease specific efficacy assessments: RA, PsA

Disease Activity Score using 28 joints (DAS28)

The DAS28 composite score includes the 28 tender and swollen joint counts, ESR and a PGA on a NRS (PGA, see above).[49] The DAS28 is calculated as follows:

**DAS28 = 0.56*sqrt(tender28) + 0.28*sqrt(swollen28) + 0.70*Ln(ESR) + 0.14*PGA**

High disease activity is defined as a DAS28 value >5.1, moderate disease activity as DAS28 >3.2 – 5.1, low disease activity as a DAS28-value of 2.6 – 3.2, and remission as DAS28 <2.6

Rheumatoid Arthritis Impact of Disease (RAID) score

The RAID questionnaire[50,51] was developed by the European League Against Rheumatism (EULAR) as a patient-derived composite score. It includes seven domains with the following relative weights: pain (0.21), functional disability (0.16), fatigue (0.15), emotional well-being (0.12), sleep (0.12), coping (0.12) and physical well-being (0.12) each rated on an NRS (0-10). See appendix 17.2. The rates of each domain are weighted and summed to form a score in the range of 0-10. It will only be used for patients with RA.

Psoriatic Arthritis Impact of Disease (PsAID) score

The PsAID questionnaire with 9 domains of health (PsAID-9) was developed by EULAR to calculate a score for clinical trials reflecting the impact of PsA from the patient’s perspective. [52] The nine domains with relative weights are: pain (0.174), fatigue (0.131), skin (0.121), work and/or leisure activities (0.110), function (0.107), discomfort (0.098), sleep (0.089), coping (0.087) and anxiety (0.085), each rated on an NRS (0-10). See appendix 17.3. The rates of each domain are weighted and summed to form a score in the range of 0-10. It will only be used for patients with PsA.

### Disease specific efficacy assessments: SpA

Bath Ankylosing Spondylitis Disease Activity Index (BASDAI)

The BASDAI was developed to define disease activity in patients with ankylosing spondylitis.[53] It includes six questions pertaining to the five major symptoms of ankylosing spondylitis: fatigue, spinal pain, joint pain/swelling, areas of localized tenderness, morning stiffness duration and morning stiffness severity. Each question is scored on an NRS (0-10). See appendix 17.4. The two morning stiffness scores are averaged and added to the average of the other scores forming a total score in the range of 0-10.

Ankylosing Spondylitis Disease Activity Score (ASDAS)

The ASDAS composite score includes

- Total back pain: NRS 0-10 (0=none, 10=very severe) according to the BASDAI Question 2 (“How would you describe the overall level of AS neck, back or hip pain you have had during the last week”)
- Patient global assessment of disease activity: NRS 0-10 (0=none, 10=Very severe) of the question ”How active was your spondylitis on average during the last week?”. The general PGA score described in section 7.1.1 will be used.
- Peripheral pain/swelling: NRS 0-10 (0=none, 10=very severe) according to the BASDAI Question 3 (“How would you describe the overall level of pain/swelling in joints other than neck, back or hip you have had during the last week”).
- Duration of morning stiffness: NRS 0-10 (0=0h, 5=1h, 10=2h or more) according to the BASDAI Question 6 (“How long does your morning stiffness last from the time you wake up during the last week?”)
- C-reactive protein (CRP) in mg/liter

The ASDAS-CRP is calculated as follows:

**ASDAS=0.121*total back pain + 0.110*patient global + 0.073*peripheral pain/swelling + 0.058*duration of morning stiffness + ln(CRP+1)**

Very high disease activity is defined as an ASDAS value >3.5, high disease activity as ASDAS 2.1 – 3.5, moderate disease activity as ASDAS 1.3 – 2.1 and inactive disease as ASDAS < 1.3.[54]

### Disease specific efficacy assessments: Ulcerative colitis

Partial Mayo Score

The Mayo score is one of the most commonly used activity indices in placebo-controlled clinical trials for ulcerative colitis.[55] It consists of four components (rectal bleeding, stool frequency, physician rating of disease activity, and mucosal appearance at endoscopy) rated from 0–3 that are summed to give a total score that ranges from 0–12. The non-invasive partial Mayo score does not require an endoscopy, and thereby ranging from 0-9 (see appendix 17.5).[27]

Remission is defined as a partial Mayo score of ≤ 2 with no individual subscore >1.

### Disease specific efficacy assessments: Crohn’s disease

Harvey-Bradshaw Index (HBI)

The Harvey-Bradshaw index[56] was presented in 1980 as a simpler version of the Crohn's disease activity index (CDAI)[57] to quantify the symptoms of Crohn’s disease. It consists of only clinical parameters. See appendix 17.6. Remission is defined as a HBI score ≤ 4 points.

### Disease specific efficacy assessments: Psoriasis

Psoriasis Area and Severity Index (PASI)

The PASI is the most commonly used activity score in clinical trials for psoriasis.[58] It is a measure of redness, thickness and scaliness of lesions (each graded 0-4), weighted by the area and location of involvement. It scores from 0 (no disease) to 72 (maximal disease severity).

Mild to moderate psoriasis is defined as PASI < 10, moderate to severe psoriasis between 10 and 20 and severe psoriasis above 20.

## Assessment of Disease Worsening

### Disease worsening in RA and PsA

A disease worsening in RA and PsA is defined as an increase in DAS28 of ≥ 1.2 from randomization and a minimum DAS score of 3.2.

### Disease worsening in SpA

A disease worsening in AS/SpA is defined as an increase in ASDAS of ≥1.1 from randomization and a minimum ASDAS of 2.1.

### Disease worsening in ulcerative colitis

A disease worsening in ulcerative colitis is defined as an increase in Partial Mayo score of ≥ 3 points from randomization and a minimum partial Mayo score of ≥ 5 points.

### Disease worsening in Crohn’s disease

A disease worsening in Crohn’s disease is defined as an increase in HBI of ≥ 4 points from randomization and a minimum HBI score of 7 points.

### Disease worsening in psoriasis

A disease worsening in psoriasis is defined as an increase in PASI of ≥ 3 points from randomization and a minimum PASI score of 5.

### Patient and investigator consensus on disease worsening

If a patient does not fulfill the formal definition, but experiences a clinically significant worsening according to both the investigator and patient and which leads to a major change in treatment this should be considered as a disease worsening but recorded separately in the CRF.

## Immunogenicity and Serum Drug Concentration Assessments

Serum samples will be drawn from all participants at all visits. The samples will be sent to the central laboratory at Oslo University Hospital, Radiumhospitalet, where serum infliximab levels will be measured using an automated immunofluorometric assay (IFMA) on the AutoDELFIA platform. Results will be recorded in a database on a secure server according to institutional guidelines, and transferred to the PI upon conclusion of the clinical trial. In exceptional cases, serum infliximab levels will be reported to clinicians during the trial upon request (see section 5.5). Antibodies to infliximab/CT-P13 will be assessed after the conclusion of the clinical trial.

## Biobank samples

Serum samples will be collected at all visits, sent to the central laboratory at Oslo University Hospital, Radiumhospitalet, for serum drug level measurements. Samples will then be aliquoted and stored in a serum biobank. Full blood samples will be collected at first visit only. All samples will be in a certified biobank in a freezer at -70° C. The samples from the biobank will be used for research purposes only, and may include measurement of cytokines and other known or potential new markers of inflammation or damage, such as interleukins, interferons, metalloproteases, transforming growth factor, TNFs, adhesion molecules etc. as well as DNA/RNA analyses (genomics and proteomics). DNA/RNA information will be used to assess possible associations between gene expressions and response/immunogenicity. Some analyses might take place in other countries if necessary.

## Safety and Tolerability Assessments

Safety will be monitored by the assessments described below as well as the collection of AEs at every visit. Significant findings that are present prior to the signing of informed consent must be included in the relevant medical history/ current medical condition page of the CRF. For details on AE collection and reporting, refer to Section 8.

### Physical examination

A general physical examination (including the cardiovascular, respiratory, GI and neurological systems) should be performed at each visit, and recorded as normal or abnormal, with a description of abnormalities. Diagnosis of new abnormalities, or worsening of abnormalities, should be recorded as an AE if appropriate.

### Vital signs

Vital signs including pulse rate, systolic and diastolic blood pressure and body weight will be assessed at all visits. Height will be measured at baseline.

### Laboratory assessments

The following laboratory tests will be recorded at all visits. Local laboratories will be used.

Hematology / complete blood count

Hemoglobin, hct, erythrocytes, white blood cells with differentials, platelet counts.

Blood chemistry

AST and/or ALT, ALP, albumine, creatinine, random glucose, potassium, sodium.

Urinalyses

Dipstick for blood, protein, glucose (with microscopic examination as clinically indicated).

Pregnancy test.

Acute phase reactants

CRP is measured by high sensitivity CRP nephelometry (mg/dL) and ESR by the Westergren method (mm/hr), according to local practice.

Fecal analysis

Calprotectin.

## Other Assessments

Modified Heath Assessment Questionnaire

The Stanford Health Assessment Questionnaire (HAQ) was introduced in the 1980s and is now widely used in evaluation of physical function in patients with inflammatory joint diseases (IJD).[59] A shortened version of the HAQ, the Modified Health Assessment Questionnaire (MHAQ)[60] reduced the number of items from 20 in the original HAQ to eight, and improved the feasibility in clinical practice. Each item is scored on a categorical 0-3 scale and the sum score is divided by 8 to form the MHAQ score 0.0 to 3.0. See appendix 17.7. The MHAQ will only be presented to patients with IJD.

Inflammatory Bowel Disease Questionnaire (IBDQ)

The IBDQ is widely used tool to measure health-related quality of life in patients with inflammatory bowel diseases. The questionnaire consists of 32 questions scored in four domains: bowel symptoms, emotional health, systemic systems and social function.[61] See appendix 17.8. The IBDQ will only be presented to patients with IBD.

Dermatology Life Quality Index (DLQI)

The DLQI is a simple self-administered, easy and user-friendly validated questionnaire used to measure the health-related quality of life of adult patients suffering from a skin disease.[62] It consists of 10 questions concerning patients' perception of the impact of skin diseases on different aspects of their health related quality of life over the last week.  It has been validated for adult dermatology patients aged 16 years and older. The items of the DLQI encompass aspects such as symptoms and feelings, daily activities, leisure, work or school, personal relationships and the side effects of treatment.   Each question is scored on a 4-point Likert scale: Not at all/Not relevant=0, A little=1, A lot=2 and Very much=3.  Scores of individual items (0-3) are added to yield a total score (0-30); higher scores mean greater impairment of patient's QoL. See appendix 17.9. The DLQI will only be presented to patients with chronic plaque psoriasis.

SF-36

The SF-36 is a multi-purpose, short-form health survey with 36 questions.[63] It yields an 8-scale profile of functional health and well-being scores as well as psychometrically-based physical and mental health summary measures and a preference-based health utility index (SF-6D).[64] It is a generic measure, as opposed to one that targets a specific age, disease, or treatment group. Accordingly, the SF-36 has proven useful in surveys of general and specific populations, comparing the relative burden of diseases, and in differentiating the health benefits produced by a wide range of different treatments. See appendix 17.10.

EQ-5D

EQ-5D is a utility instrument for measurement of health related quality of life.[65,66] Applicable to a wide range of health conditions and treatments, it provides a simple descriptive profile and a single index value for health status. See appendix 17.11.

Work Productivity and Activity Impairment Questionnaire: General Health (WPAI:GH)

Worker productivity is generally subdivided into 2 components: absenteeism and presenteeism. The concept of absenteeism has been defined as productivity loss due to health-related absence from work, while presenteeism refers to reduced performance or productivity while at work due to health reasons. Absenteeism may include personal time off, sick days off work, time on short and/or long-term work disability, or time on worker’s-compensated days; and presenteeism could be characterized as the time not being on the task, or decreased work quality and quantity. Patients will be asked to answer the Work Productivity and Activity Impairment Questionnaire: General Health V2.0 (WPAI:GH).[67] See appendix 17.12.

The WPAI yields four types of scores:

1.  Absenteeism (work time missed)

2.  Presenteeism (impairment at work/reduced on-the-job effectiveness)

3.  Work productivity loss (overall work impairment / absenteeism plus presenteeism)

4.  Activity Impairment

Resource use

The following types of resource use will be captured:

- Use of biologics
- Use of other pharmaceuticals (Norwegian Prescription Database)
- Use of somatic hospital services (in-patient and out-patient)(Norwegian Patient Register)
- Use of GP services and emergency room services (HELFO database - The Norwegian Health Economics Administration database)
- Use of social benefits (NAV)

Simplified Psoriasis Index (SPI)

The Simplified Psoriasis Index (SPI) is a summary measure of psoriasis with separate components for current severity (SPI-s), psychosocial impact (SPI-p), and past history and interventions (SPI-i).[68] A pseudoscore for SPI is computed for this study using the PASI score multiplied by 1.13 (as estimated in [68]) for the SPI-s score, the Patient Global Assessment of Disease Activity (PGA) for SPI-p and baseline information for SPI-i.

# Safety MONItoring and reporting

The investigator is responsible for the detection and documentation of events meeting the criteria and definition of an adverse event (AE) or serious adverse event (SAE). Each patient will be instructed to contact the investigator immediately should they manifest any signs or symptoms they perceive as serious.

The methods for collection of safety data are described below.

## Definitions

### Adverse Event (AE)

An AE is any untoward medical occurrence in a patient administered a pharmaceutical product and which does not necessarily have a causal relationship with this treatment.

An adverse event (AE) can therefore be any unfavorable and unintended sign (including an abnormal laboratory finding), symptom, or disease temporally associated with the use of a medicinal (investigational) product, whether or not related to the medicinal (investigational) product.

The term AE is used to include both serious and non-serious AEs.

If an abnormal laboratory value/vital sign are associated with clinical signs and symptoms, the sign/symptom should be reported as an AE and the associated laboratory result/vital sign should be considered additional information that must be collected on the relevant CRF.

### Serious Adverse Event (SAE)

Any untoward medical occurrence that at any dose:

1. Results in death
2. Is immediately life-threatening
3. Requires in-patient hospitalization or prolongation of existing hospitalization
4. Results in persistent or significant disability or incapacity
5. Is a congenital abnormality or birth defect
6. Is an important medical event that may jeopardize the subject or may require medical intervention to prevent one of the outcomes listed above

Medical and scientific judgment is to be exercised in deciding on the seriousness of a case. Important medical events may not be immediately life-threatening or result in death or hospitalization, but may jeopardize the subject or may require intervention to prevent one of the listed outcomes in the definitions above. In such situations, or in doubtful cases, the case should be considered as serious. Hospitalization for administrative reason (for observation or social reasons) is allowed at the investigator’s discretion and will not qualify as serious unless there is an associated adverse event warranting hospitalization.

### Suspected Unexpected Serious Adverse Reaction (SUSAR)

Adverse Reaction: all untoward and unintended responses to an investigational medicinal product related to any dose administered;

Unexpected Adverse Reaction: an adverse reaction, the nature or severity of which is not consistent with the applicable product information.

Suspected Unexpected Serious Adverse Reaction: SAE (see section 8.1.2) that is unexpected as defined in section 8.2 and possibly related to the investigational medicinal product(s).

## Expected Adverse Events

Expected AEs/SAEs for the IMPs according to the IMPs Summary of Product Characteristics (SmPC) will be recorded in the eCRF.

## Disease Progression/Recurrence

Events which are definitely due to disease progression will not be reported as an AE/SAE. However, if the investigator considers that there was a causal relationship between treatment with IMP or protocol design/procedures and the disease progression/recurrence, then this must be reported as an AE/SAE.

## Time Period for Reporting AE and SAE

For each patient the standard time period for collecting and recording AE and SAEs will begin at start of study treatment and will continue for at least 30 days following the last dose of study treatment for each patient.

During the course of the study all AEs and SAEs will be proactively followed up for each patient. Events should be followed up to resolution, unless the event is considered by the investigator to be unlikely to resolve due to the underlying disease. Every effort should be made to obtain a resolution for all events, even if the events continue after discontinuation/study completion.

## Recording of Adverse Events

If the patient has experienced adverse event(s), the investigator will record the following information in the CRF:

- The nature of the event(s) will be described by the investigator in precise standard medical terminology (i.e. not necessarily the exact words used by the patient).
- The duration of the event will be described in terms of event onset date and event ended data.
- The intensity of the adverse event will be described according to Common Terminology Criteria for Adverse Events version 4.0 (CTCAE)
- The Causal relationship of the event to the study medication will be assessed as one of the following:

Unrelated:

There is not a temporal relationship to the administration of IMP (too early, or late, or IMP not taken), or there is a reasonable causal relationship between non-IMP, concurrent disease, or circumstance and the AE.

Unlikely:

There is a temporal relationship to IMP administration, but there is not a reasonable causal relationship between the investigational product and the AE.

Possible:

There is reasonable causal relationship between the IMP and the AE. Dechallenge information is lacking or unclear.

Probable:

There is a reasonable causal relationship between the IMP and the AE. The event responds to dechallenge. Rechallenge is not required.

Definite:

There is a reasonable causal relationship between the IMP and the AE.

- Action taken
- The outcome of the adverse event – whether the event is resolved or still ongoing.

It is important to distinguish between serious and severe AEs. Severity is a measure of intensity whereas seriousness is defined by the criteria in Section 8.1.2. An AE of severe intensity need not necessarily be considered serious. For example, nausea that persists for several hours may be considered severe nausea, but is not an SAE. On the other hand, a stroke that results in only a limited degree of disability may be considered a mild stroke, but would be an SAE.

## Reporting Procedure

### AEs and SAEs

All adverse events and serious adverse events that should be reported as defined in section 8.1.1 will be recorded in the patient's eCRF.

SAEs must be reported by the investigator to the sponsor as outlined in the ISF within 24 hours after the site has gained knowledge of the SAE. Every SAE must be documented by the investigator on the SAE pages in the eCRF. The initial report shall promptly be followed by detailed, written reports if necessary. The initial and follow-up reports shall identify the trial subjects by unique code numbers assigned to the latter.

The sponsor keeps detailed records of all SAEs reported by the investigators and performs an evaluation with respect to seriousness, causality and expectedness.

### SUSARs

SUSARs will be reported to the Competent Authority according to national regulation. The following timelines should be followed:

The sponsor will ensure that all relevant information about suspected serious unexpected adverse reactions that are fatal or life-threatening is recorded and reported as soon as possible to the Competent Authority, and in any case no later than seven (7) days after knowledge by the sponsor of such a case, and that relevant follow-up information is subsequently communicated within an additional eight (8) days.

All other suspected serious unexpected adverse reactions will be reported to the Competent Authority concerned and to the Ethics Committee concerned as soon as possible but within a maximum of fifteen (15) days of first knowledge by the sponsor.

SUSARs will be reported using the CIOMS form since Diakonhjemmet Hospital is not connected to EudraVigilance.

### Annual Safety Report

Once a year throughout the clinical trial, the sponsor will provide the Competent Authority with an annual safety report. The format will comply with national requirements.

### Clinical Study Report

The adverse events and serious adverse events occurring during the study will be discussed in the safety evaluation part of the Clinical Study Report.

## Procedures in Case of Emergency

The investigator is responsible for assuring that there are procedures and expertise available to cope with emergencies during the study. All patients will at inclusion receive a card with contact information about the sponsor and local investigator, as well as emergency contact information. As described in section 6.2.4 all patients will receive an extra visit within one week in case of suspected flare in disease activity.

In the event of an SAE, the Investigator may only break the treatment code if the appropriate future management of the patient necessitates knowledge of the current treatment. Although it is advantageous to retain the blind for all patients prior to final trial analysis, when an SAE may be a serious adverse reaction unexpected or otherwise judged reportable on an expedited basis, it is recommended that the blind should be broken only for that specific patient, by the sponsor or its designee, even if the Investigator has not broken the blinding.

# Data management and monitoring

## Electronic Case Report Forms (CRFs)

The designated investigator staff will enter the data required by the protocol into the electronic Case report forms (eCRF). The Principal Investigator is responsible for assuring that data entered into the eCRF is complete, accurate, and that entry is performed in a timely manner. The electronic signature of the investigator will attest the accuracy of the data on each CRF. If any assessments are omitted, the reason for such omissions will be noted on the CRFs. Corrections, with the reason for the corrections will also be recorded. A complete list of authorized study personnel will be maintained during the study, and only study personnel authorized by the principal investigator or coordinating investigator will be allowed to sign the eCRF.

After database lock, the investigator will receive a CD-ROM or paper copies of the subject data for archiving at the investigational site.

A web-based eCRF software solution that adheres to GCP will be used to collect study data (Viedoc™, Uppsala, Sweden).

## Source Data

The medical records for each patient should contain information, which is important for the patient’s safety and continued care, and to fulfill the requirement that critical study data should be verifiable.

To achieve this, the medical records of each patient should clearly describe at least:

- That the patient is participating in the study, e.g. by including the enrollment number and the study code or other study identification;
- Date when Informed Consent was obtained from the patient and statement that patient received a copy of the signed and dated Informed Consent;
- Results of all assessments confirming a patient’s eligibility for the study;
- Diseases (past and current; both the disease studied and others, as relevant);
- Surgical history, as relevant;
- Treatments withdrawn/withheld due to participation in the study;
- Results of assessments performed during the study;
- WHO performance status assessments conducted as part of the study, if applicable;
- Treatments given, changes in treatments during the study and the time points for the changes;
- Visits to the clinic / telephone contacts during the study, including those for study purposes only;
- Non-Serious Adverse Events and Serious Adverse Events (if any) including causality assessments;
- Date of, and reason for, discontinuation from study treatment;
- Date of, and reason for, withdrawal from study;
- Date of death and cause of death, if available;
- Additional information according to local regulations and practice.

Patient reported outcome (PRO) measures such as SF-36, EQ-5D, M-HAQ, IBDQ and DLQI not recorded in an electronic patient journal (EPJ) system is recorded on paper CRFs or directly into the eCRF. If these measures are recorded directly in the eCRF, the eCRF is source data. If they are recorded on paper and then entered into the eCRF, then the paper CRF is source data.

## Study Monitoring

The investigator will be visited on a regular basis by the Clinical Study Monitor, who will check the following:

- Informed consent process
- Reporting of adverse events and other safety data
- Adherence to protocol
- Maintenance of required regulatory documents
- Study Supply accountability
- Facilities and equipment (example: laboratory)
- Data completion on the eCRFs including source data verification (SDV).

The monitor will review the relevant eCRFs for accuracy and completeness and will ask the site staff to adjust any discrepancies as required.

Monitoring of data completion will be performed within the eCRF solution. When the responsible study monitor has checked and verified the CRFs, the data will be locked for further handling and statistical evaluation.

Sponsor’s representatives (e.g. monitors, auditors) and/or competent authorities will be allowed access to source data for source data verification in which case a review of those parts of the hospital records relevant to the study may be required.

## Confidentiality

The investigator shall arrange for the secure retention of the patient identification and the code list. Patient files shall be kept for the maximum period of time permitted by each hospital. The study documentation (CRFs, Site File etc.) shall be retained and stored during the study and for 15 years after study closure. All information concerning the study will be stored in a safe place inaccessible to unauthorized personnel.

## Database management

A separate data management plan will be written describing the set-up and quality control of the eCRF, data entering procedures, range and logical checks, coding, security and coding.

# Statistical methods and data analysis

## Determination of Sample Size

Sample size calculation has been based on the primary endpoint (proportion of patients experiencing disease worsening during the study period of 52 weeks). Data from the Norwegian NOR-DMARD registry showed a yearly INX treatment failure rate of 18% per year in patients on stable INX for at least one year. Expecting a higher occurrence of disease worsening compared to treatment failure, we assume that 30% of the patients will experience a disease worsening within 52 weeks. A non-inferiority margin of 15% was regarded appropriate based on the PLANETRA study [47] and discussions with the Norwegian regulatory body.

If there is truly no difference between INX and CT-P13 treatment on the proportion of patients with disease worsening after 52 weeks, 394 patients (197 in each arm) are required to be 90% sure that the upper limit of a one-sided 97.5% confidence interval (or equivalently a two-sided 95% confidence interval) will exclude a difference in favor of INX of more than 15%. Different combinations of remission rates and non-inferiority margins are summarized in Table 1 and Table 2.

Because of the non-inferiority design, the primary population will be the per-protocol (PP) population. To adjust for protocol violators (estimated to 20%), a total of 492 (246 in each arm) will be randomized.

Table 1: The numbers in the cells represent the total number of patients needed in total. All calculations are based on a power of 80% and alpha 2.5%

| ***Non-inferiority Margin*** | ***10% disease worsening at 48 w*** | ***20% disease worsening at 48 w*** | ***30% disease worsening at 48 w*** |
| --- | --- | --- | --- |
| **10%** | 248 | 504 | 660 |
| **15 %** | 126 | 224 | 294 |
| **20 %** | 72 | 126 | 166 |

Table 2: The numbers in the cells represent the total number of patients needed in total. All calculations are based on a power of 90% and alpha 2.5%.

| ***Non-inferiority Margin*** | ***10% disease worsening at 48 w*** | ***20% disease worsening at 48 w*** | ***30% disease worsening at 48 w*** |
| --- | --- | --- | --- |
| **10%** | 380 | 674 | 884 |
| **15 %** | 170 | 300 | 394 |
| **20 %** | 96 | 170 | 222 |

## Randomization

### Allocation- sequence generation

Eligible patients will be allocated in a 1:1 ratio between INX and CT-P13 treatment, using a computer randomization procedure stratified by diagnosis (RA, SpA, PsA, UC, CD, chronic plaque psoriasis). The randomization will be blocked within each stratum.

Details of block size and allocation sequence generation will be provided in a separate document that is unavailable to those who enroll patients or assign treatment.

### Allocation- procedure to randomize a patient

The computer-generated randomized allocation sequence will be imported into the eCRF system and made available to site personnel responsible for study treatment preparation. Only personnel authorized by the principal investigator for preparing treatment will have access to treatment allocation, and the allocation will not be available until the patient has signed the informed consent form and deemed eligible to participate in the study. That is, authorized personnel will only know the allocation of included patients, but not for future patients.

Once the patient has been included, the site person authorized for treatment preparation will log into the eCRF system to reveal the allocation. Further procedure will be as follows:

1. Prepare the allocated treatment for administration
2. Record the batch number and date, time and dose of administration into the eCRF
3. Prepare and append label to the infusion bag
4. Dispatch all packaging identifying the allocated treatment
5. Log out of the eCRF system to prevent blinded site staff knowledge of allocation

Site staff should take measures to maintain the blinding, e.g. not storing medication such that it is evident which treatment has been given by identifying missing medication.

### Blinding and emergency unblinding

The following personnel will be un-blinded to allocation:

- The statistician preparing the randomized allocation sequence
- The data manager importing the allocation sequence into the eCRF system and providing access to the allocation sequence
- Site personnel authorized to prepare study treatment
- Other study personnel authorized to be un-blinded, e.g. laboratory personnel responsible for drug concentration analyses etc.

Authorized site personnel will only have access to allocation of patients included at the specific site, and to no other sites.

All other persons will be blinded to treatment allocation, including

- Patients
- Investigators
- Personnel assessing outcomes
- Monitors

Un-blinding of the treatment allocation is permissible only if the safety and well-being of the patient is being compromised. The decision to reveal the treatment allocation during the study may only be done by the principal investigator. The date and time of un-blinding must be documented in the eCRF and in the patient’s hospital records.

In the event of an SAE, the Investigator may only break the treatment code if the appropriate future management of the patient necessitates knowledge of the current treatment. Although it is advantageous to retain the blind for all patients prior to final trial analysis, when an SAE may be a serious adverse reaction unexpected or otherwise judged reportable on an expedited basis, it is recommended that the blind should be broken only for that specific patient, by the sponsor or its designee, even if the Investigator has not broken the blinding.

All study personnel will be unblinded to the treatment during the open-label follow-up period (but still blinded for the treatment during the preceding intervention period).

## Population for Analysis

The following populations will be considered for the analyses:

- The intention to treat (ITT) population is defined as all randomized patients, regardless of protocol adherence.
- The per-protocol population (PP) is a subset of the ITT population and consists of patients who sufficiently comply with the protocol. Criteria for inclusion in the PP population will be specified in the statistical analysis plan, and the final criteria will be defined prior to database lock.
- The follow-up population is defined as all patients entering the open-label follow-up period.
- The safety population will include all subjects who have received at least one dose of study medication. Subjects who withdraw from the study will be included in the safety analysis. A list of withdrawn subjects, with reasons for withdrawal, will be made.

In superiority trials, the norm is to perform the primary analysis on the ITT population. This is regarded a conservative approach because inclusion of patients with poor compliance to protocol will presumably bias the analysis towards the null hypothesis (no treatment difference). In a non-inferiority trial, the aim is to show no clinically significant treatment difference, and the intention-to-treat principle is no longer regarded as conservative. The primary analysis will therefore be performed on the PP population.

## Planned analyses

The main statistical analysis is planned when

- The planned number of patients have been included
- All included patients have either finalized their last assessment of the intervention period or has/is withdrawn according to protocol procedures
- All data from the intervention period have been entered, verified and validated according to the data management plan

Prior to the main statistical analysis, the data base will be locked for further entering or altering of data. A separate statistical analysis plan (SAP) will provide further details on the planned statistical analyses. The SAP will be finalized, signed and dated prior to database lock. The treatment allocation will be revealed after the database lock and used in the statistical analysis. There will be no interim analysis.

Deviation from the original statistical plan will be described and justified in the Clinical Study Report.

## Statistical Analysis

### Statistical model

This randomized clinical trial aims primarily to describe and estimate efficacy parameters and test pre-specified statistical hypotheses.

The primary variable will be analyzed using a logistic regression model. There will be two baseline covariates included in the analysis: the stratification factor used at randomization (diagnosis), and the treatment duration of INX at baseline. Although this is a multicenter study, study site will not be used for stratification or adjustment in the analysis due to small sample sizes within site. However, sensitivity analyses will be performed to assess the impact of site adjustment on the study conclusions. Other pre-specified covariates included in sensitivity analyses include age, use of disease-specific co-medication (methotrexate, azathioprine or similar) and levels of neutralizing antibodies at baseline. The statistical analysis plan (SAP) will detail these procedures, as well as alternative and further supportive evaluations, such as analyses including unbalanced baseline predictors or modifications of the logistic regression model in case validity assumptions are not met.

The primary analysis will be performed on the primary per protocol population.

### Primary variable

Statistical hypothesis (non-inferior test)

Null hypothesis: The difference in proportion of patients who experiences disease worsening is at least 15% in favor of INX compared to CT-P13.

Alternative hypothesis: The difference in proportion of patients who experiences disease worsening is less than 15% in favor of INX compared to CT-P13.

The primary variable will be evaluated by the 95% confidence limits. A conclusion of non-inferiority will be made if the 95% confidence limits of the estimated treatment difference fully lie within the inferiority margin of 15%. The inferiority margin of 15% was regarded appropriate based on the PLANETRA study [47] and discussions with the Norwegian regulatory body.

If the data indicate that one of the groups is superior to the other, a superiority test on the 5% significance level will be performed according to the following hypothesis:

Statistical hypothesis (superiority test):

Null hypothesis: There is no difference between INX and CT-P13 with regards to the proportion of patients who experiences disease worsening.

Alternate hypothesis: There is a difference between INX and CT-P13 with regards to the proportion of patients who experiences disease worsening, i.e. a two-sided test will be employed.

This analysis will be performed on the ITT population.

### Secondary/exploratory variables

Between-group comparisons will be performed for the primary endpoint on secondary populations in addition to secondary efficacy endpoints on all populations. The order of secondary statistical hypothesis tests will be prespecified to enable a valid closed test procedure and thereby controlling the familywise error rate. The order will be presented in the SAP.

Hypotheses testing

The primary efficacy analyses will be performed using a logistic regression model (see section 10.5.1 above for details on statistical model).

The between-group comparisons for secondary variables will be tested as for the primary variable where applicable and additional analyses will be performed based on the following methods (but not limited to):

- Continuous secondary variables will be subject to repeated measures mixed models or appropriate non-parametric alternatives

- Binary response variables will be analyzed using logistic regression (possibly adjusting for within-subject dependencies by generalized estimating equations) or chi-square/Mantel-Haenszel test

- Time-to-event variables will be analyzed using the Kaplan-Meier method and comparisons between the two groups will be performed using the log rank test or Cox regression analyses.

Unless otherwise specified, all statistical hypotheses will be tested as the primary variable, i.e. with an assessment of non-inferiority based on the 95% confidence limits of the estimated difference between the groups. Non-inferiority margins of the secondary endpoints will be specified in the SAP prior to analysis. If the data indicate that one of the groups is superior to the other, a superiority test on the 5% significance level will be performed as for the primary variable on the ITT population. Thus, the non-inferiority analyses will be done in the PP population and the superiority analyses in the ITT population as appropriate.

Efficacy analyses

All patients included in the PP population will form the primary analysis population of the study. All efficacy analyses will be presented with the results from the hypothesis testing (by p-value) in addition to estimates and 95% confidence limits of the treatment effect. For the primary variable specifically, this will be the estimated odds ratio and relative risk with corresponding 95% confidence limits.

Safety analyses

The safety analyses population will include all patients who completed at least one follow-up visit. Safety analyses will be descriptive and presented as summary tables by treatment group and (if applicable) by visit.

Quality of life and disability analyses

Quality of life and disability will be assessed using SF-36, EQ-5D, MHAQ (IJD), IBDQ (IBD) and DLQI (chronic plaque psoriasis). These scores will be summarized by descriptive summary tables at baseline and over time, and at the end of study. Missing data at end of study will be replaced by the last valid post-baseline assessment.

Other analyses/subanalyses

We will perform subgroup analyses according to diagnoses groups (RA, SpA, PsA, UC, CD, chronic plaque psoriasis) on the appropriate primary and secondary variables using methods described above.

Other exploratory subgroup analyses of primary, secondary and exploratory efficacy variables may be performed if appropriate. The decision to include such analyses will be made on basis of the collected data.

24-week open-label follow-up period

Patients switching from INX to CT-P13 will be compared with patients who continued treatment wCT-P13.

### Health economic analyses

All patients will, with assistance from a study nurse, be asked to fill in the two standard instruments (questionnaires) to capture health related quality of life (HRQOL): SF-36 and EQ-5D. These instruments will be used at each visit.

Use of health care (costs) will be captured by the following registers: The Norwegian Patient Register (hospital services), The Norwegian Prescription Register (pharmaceuticals), The Norwegian Health Economics Administration database (emergency room and general practitioner services), Statistics Norway KOSTRA database (nursing services) and the Norwegian Welfare and Labour Administration NAV (social benefits). We will assign unit costs to each type of service by means of the DRG price list, and the price list of the Norwegian Medicines Agency (Innsatsstyrt finansiering 2013;Normaltariff for privat spesialistpraksis 2012-2013;Moger and Kristiansen).

For each patient we will, based on HRQOL data, estimate the number of QALYs obtained during the 52-week study period in line with methods used previously (Bohmer et al. 717-23;Fjalestad et al. 599-605) and adjust for any baseline imbalances (Manca, Hawkins, and Sculpher 487-96). We will use EQ-5D and also translate SF-36-data into utilities according to a validated method (Brazier, Roberts, and Deverill 271-92). For each patient we will estimate one year costs based on register data for utilization of health care and the unit costs.

The mean 52-week QALYs and cost in the two treatment arms will be used to estimate an incremental cost-effectiveness ratio (ICER), for all patients and according to diagnostic group. Not all patients in the randomized trial will have complete 12 months data. We will therefore impute missing data (Glick and Doshi). We will use bootstrapping to estimate confidence intervals of the incremental costs and QALYs and to present uncertainty in cost-effectiveness acceptability curves.

### Missing data

In the primary per protocol population, we expect few or no missing data. If, however, missing data is regarded as having a significant effect on the conclusions of the trial, sensitivity analyses with different methods for handling missing data will be included. Such methods may include complete case analyses, last observation carried forward, worst case/best case imputation and multiple imputation techniques.

# STUDY MANAGEMENT

The Study will be managed within the NorCRIN network. The NorCRIN secretariat will:

- Draw up contracts between sponsor and the individual trial sites
- Draw up contracts between sponsor and third-parties
- Set-up of the electronic Trial Master File (TMF)
- Prepare Investigator Site Files (ISFs)
- Contribute to trial initiation meetings
- Facilitate a channel for public study information
- Prepare and send out news letters

## Investigator Delegation Procedure

The principal investigator is responsible for making and updating a “delegation of tasks” listing all the involved co-workers and their role in the project. He will ensure that appropriate training relevant to the study is given to all of these staff, and that any new information of relevance to the performance of this study is forwarded to the staff involved.

## Protocol Adherence

Investigators ascertain they will apply due diligence to avoid protocol deviations.

All significant protocol deviations will be recorded and reported in the Clinical Study Report (CSR).

## Study Amendments

If it is necessary for the study protocol to be amended, the amendment and/or a new version of the study protocol (Amended Protocol) must be notified to and approved by the Competent Authority and the Ethics Committee according to EU and national regulations.

## Audit and Inspections

Authorized representatives of a Competent Authority and Ethics Committee may visit the center(s) to perform inspections, including source data verification. Likewise the representatives from the sponsor may visit the center(s) to perform an audit. The purpose of an audit or inspection is to systematically and independently examine all study-related activities and documents to determine whether these activities were conducted, and data were recorded, analyzed, and accurately reported according to the protocol, Good Clinical Practice (ICH GCP), and any applicable regulatory requirements. The principal investigator will ensure that the inspectors and auditors will be provided with access to source data/documents.

# Ethical and regulatory requirements

The study will be conducted in accordance with ethical principles that have their origin in the Declaration of Helsinki and are consistent with ICH/Good Clinical Practice and applicable regulatory requirements. Registration of patient data will be carried out in accordance with national personal data laws.

## Ethics Committee Approval

The study protocol, including the patient information and informed consent form to be used, must be approved by the regional ethics committee before enrolment of any patients into the study.

The principle investigator is responsible for informing the ethics committee of any serious and unexpected adverse events and/or major amendments to the protocol as per national requirements.

## Other Regulatory Approvals

The protocol will be submitted and approved by the applicable competent authorities before commencement of the study.

The protocol will also be registered in www.clinicaltrials.gov before inclusion of the first patient.

## Informed Consent Procedure

The investigator is responsible for giving the patients full and adequate verbal and written information about the nature, purpose, possible risk and benefit of the study. They will be informed as to the strict confidentiality of their patient data, but that their medical records may be reviewed for trial purposes by authorized individuals other than their treating physician.

It will be emphasized that the participation is voluntary and that the patient is allowed to refuse further participation in the protocol whenever she/he wants. This will not prejudice the patient’s subsequent care. Documented informed consent must be obtained for all patients included in the study before they are registered in the study. This will be done in accordance with the national and local regulatory requirements. The investigator is responsible for obtaining signed informed consent.

A copy of the patient information and consent will be given to the patients. The signed and dated patient consent forms will be filed in the Investigator Site File binder.

In order to proceed into the open-label follow-up period after the intervention period, patients will be given full and adequate verbal and written information about the nature, purpose, possible risk and benefit of attending the follow-up period. A copy of the patient information and consent for the follow-up period will be given to the patients, and the signed and dated patient consent form will be filed in the Investigator Site File Binder.

## Subject Identification

The investigator is responsible for keeping a list of all patients (who have received study treatment or undergone any study specific procedure) including patient’s date of birth and personal number, full names and last known addresses.

The patients will be identified in the eCRFs by patient number, initials and date of birth.

# Trial sponsorship and financing

The study is sponsored by funds at Diakonhjemmet Hospital, which has been received from the Regional Health Authority Helse Sør-Øst. The total funding of the study of 20 million NOK is based on a specific allocation in the governmental budget for 2014.

# Trial insurance

The Principal investigator has insurance coverage for this study through membership of the Drug Liability Association (see http://www.laf.no for more details).

# Publication policy

Upon study completion and finalization of the study report the results of this study will either be submitted for publication and/or posted in a publicly assessable database of clinical study results.

The results of this study will also be submitted to the Competent Authority and the Ethics Committee according to EU and national regulations.

All personnel who have contributed significantly with the planning and performance of the study (Vancouver convention 1988) may be included in the list of authors.

# REFERENCES

1 Kvien TK, Glennas A, Knudsrod OG, *et al.* The prevalence and severity of rheumatoid arthritis in Oslo. Results from a county register and a population survey. *Scand J Rheumatol* 1997;**26**:412–8.

2 Uhlig T, Kvien TK, Glennas A, *et al.* The incidence and severity of rheumatoid arthritis, results from a county register in Oslo, Norway. *J Rheumatol* 1998;**25**:1078–84.

3 Aletaha D, Neogi T, Silman AJ, *et al.* 2010 rheumatoid arthritis classification criteria: an American College of Rheumatology/European League Against Rheumatism collaborative initiative. *Ann Rheum Dis* 2010;**69**:1580–8. doi:10.1136/ard.2010.138461

4 van der Heijde D, Sieper J, Maksymowych WP, *et al.* 2010 Update of the international ASAS recommendations for the use of anti-TNF agents in patients with axial spondyloarthritis. *Ann Rheum Dis* 2011;**70**:905–8. doi:10.1136/ard.2011.151563

5 Bakland G, Nossent HC. Epidemiology of spondyloarthritis: a review. *Curr Rheumatol Rep* 2013;**15**:351. doi:10.1007/s11926-013-0351-1

6 Haglund E, Bremander AB, Petersson IF, *et al.* Prevalence of spondyloarthritis and its subtypes in southern Sweden. *Ann Rheum Dis* 2011;**70**:943–8. doi:10.1136/ard.2010.141598

7 Rudwaleit M, van der Heijde D, Landewe R, *et al.* The Assessment of SpondyloArthritis International Society classification criteria for peripheral spondyloarthritis and for spondyloarthritis in general. *Ann Rheum Dis* 2011;**70**:25–31. doi:10.1136/ard.2010.133645

8 Taylor W, Gladman D, Helliwell P, *et al.* Classification criteria for psoriatic arthritis: development of new criteria from a large international study. *Arthritis Rheum* 2006;**54**:2665–73. doi:10.1002/art.21972

9 Sartor RB. Mechanisms of disease: pathogenesis of Crohn's disease and ulcerative colitis. *Nature clinical practice Gastroenterology & hepatology* 2006.

10 Nestle FO, Kaplan DH, Barker J. Psoriasis. *N Engl J Med* 2009;**361**:496–509. doi:10.1056/NEJMra0804595

11 Gelfand JM, Troxel AB, Lewis JD, *et al.* The risk of mortality in patients with psoriasis: results from a population-based study. *Arch Dermatol* 2007;**143**:1493–9. doi:10.1001/archderm.143.12.1493

12 Parisi R, Symmons DPM, Griffiths CEM, *et al.* Global epidemiology of psoriasis: a systematic review of incidence and prevalence. *J Invest Dermatol* 2013;**133**:377–85. doi:10.1038/jid.2012.339

13 Danielsen K, Olsen AO, Wilsgaard T, *et al.* Is the prevalence of psoriasis increasing? A 30-year follow-up of a population-based cohort. *Br J Dermatol* 2013;**168**:1303–10. doi:10.1111/bjd.12230

14 Meier M, Sheth PB. Clinical spectrum and severity of psoriasis. *Curr Probl Dermatol* 2009;**38**:1–20. doi:10.1159/000232301

15 Gottlieb A, Korman NJ, Gordon KB, *et al.* Guidelines of care for the management of psoriasis and psoriatic arthritis: Section 2. Psoriatic arthritis: overview and guidelines of care for treatment with an emphasis on the biologics. *J Am Acad Dermatol* 2008;**58**:851–64. doi:10.1016/j.jaad.2008.02.040

16 Mehta NN, Yu Y, Pinnelas R, *et al.* Attributable risk estimate of severe psoriasis on major cardiovascular events. *Am J Med* 2011;**124**:775.e1–6. doi:10.1016/j.amjmed.2011.03.028

17 Rapp SR, Feldman SR, Exum ML, *et al.* Psoriasis causes as much disability as other major medical diseases. *J Am Acad Dermatol* 1999;**41**:401–7.

18 Meyer N, Paul C, Feneron D, *et al.* Psoriasis: an epidemiological evaluation of disease burden in 590 patients. *J Eur Acad Dermatol Venereol* 2010;**24**:1075–82. doi:10.1111/j.1468-3083.2010.03600.x

19 Ryan C, Korman NJ, Gelfand JM, *et al.* Research gaps in psoriasis: opportunities for future studies. *J Am Acad Dermatol* 2014;**70**:146–67. doi:10.1016/j.jaad.2013.08.042

20 Smolen JS, Landewe R, Breedveld FC, *et al.* EULAR recommendations for the management of rheumatoid arthritis with synthetic and biological disease-modifying antirheumatic drugs. *Ann Rheum Dis* 2010;**69**:964–75. doi:10.1136/ard.2009.126532

21 Braun J, van den Berg R, Baraliakos X, *et al.* 2010 update of the ASAS/EULAR recommendations for the management of ankylosing spondylitis. *Ann Rheum Dis* 2011;**70**:896–904. doi:10.1136/ard.2011.151027

22 Smolen JS, Landewe R, Breedveld FC, *et al.* EULAR recommendations for the management of rheumatoid arthritis with synthetic and biological disease-modifying antirheumatic drugs: 2013 update. *Ann Rheum Dis* 2014;**73**:492–509. doi:10.1136/annrheumdis-2013-204573

23 Kavanaugh A, Ritchlin C, Rahman P, *et al.* Ustekinumab, an anti-IL-12/23 p40 monoclonal antibody, inhibits radiographic progression in patients with active psoriatic arthritis: results of an integrated analysis of radiographic data from the phase 3, multicentre, randomised, double-blind, placebo-controlled PSUMMIT-1 and PSUMMIT-2 trials. *Ann Rheum Dis* 2014;**73**:1000–6. doi:10.1136/annrheumdis-2013-204741

24 Dignass A, Lindsay JO, Sturm A, *et al.* Second European evidence-based consensus on the diagnosis and management of ulcerative colitis Part 2: Current management. *J Crohns Colitis* 2012;**6**:991–1030. doi:10.1016/j.crohns.2012.09.002

25 Dignass A, Van Assche G, Lindsay JO, *et al.* The second European evidence-based Consensus on the diagnosis and management of Crohn's disease: Current management. *J Crohns Colitis* 2010;**4**:28–62. doi:10.1016/j.crohns.2009.12.002

26 Mehta SJ, Silver AR, Lindsay JO. Review article: strategies for the management of chronic unremitting ulcerative colitis. *Aliment Pharmacol Ther* 2013;**38**:77–97. doi:10.1111/apt.12345

27 Rutgeerts P, Sandborn WJ, Feagan BG, *et al.* Infliximab for induction and maintenance therapy for ulcerative colitis. *N Engl J Med* 2005;**353**:2462–76. doi:10.1056/NEJMoa050516

28 Hanauer SB, Feagan BG, Lichtenstein GR, *et al.* Maintenance infliximab for Crohn's disease: the ACCENT I randomised trial. *Lancet* 2002;**359**:1541–9. doi:10.1016/S0140-6736(02)08512-4

29 Colombel J-F, Sandborn WJ, Rutgeerts P, *et al.* Adalimumab for maintenance of clinical response and remission in patients with Crohn's disease: the CHARM trial. *Gastroenterology* 2007;**132**:52–65. doi:10.1053/j.gastro.2006.11.041

30 Sandborn WJ, Feagan BG, Marano C, *et al.* Subcutaneous golimumab induces clinical response and remission in patients with moderate-to-severe ulcerative colitis. *Gastroenterology* 2014;**146**:85–95–quize14–5. doi:10.1053/j.gastro.2013.05.048

31 Moss AC, Brinks V, Carpenter JF. Review article: immunogenicity of anti-TNF biologics in IBD - the role of patient, product and prescriber factors. *Aliment Pharmacol Ther* 2013;**38**:1188–97. doi:10.1111/apt.12507

32 Solberg IC, Lygren I, Jahnsen J, *et al.* Clinical course during the first 10 years of ulcerative colitis: results from a population-based inception cohort (IBSEN Study). *Scand J Gastroenterol* 2009;**44**:431–40. doi:10.1080/00365520802600961

33 Penna C, Dozois R, Tremaine W, *et al.* Pouchitis after ileal pouch-anal anastomosis for ulcerative colitis occurs with increased frequency in patients with associated primary sclerosing cholangitis. *Gut* 1996;**38**:234–9.

34 El-Hachem S, Regueiro M. Postoperative Crohn's disease: prevention and treatment. *Expert Rev Gastroenterol Hepatol* 2009;**3**:249–56. doi:10.1586/egh.09.21

35 Papoutsaki M, Costanzo A. Treatment of psoriasis and psoriatic arthritis. *BioDrugs* 2013;**27 Suppl 1**:3–12. doi:10.1007/BF03325637

36 Dubertret L, Mrowietz U, Ranki A, *et al.* European patient perspectives on the impact of psoriasis: the EUROPSO patient membership survey. *Br J Dermatol* 2006;**155**:729–36. doi:10.1111/j.1365-2133.2006.07405.x

37 Mason A, Mason J, Cork M, *et al.* Topical treatments for chronic plaque psoriasis: an abridged Cochrane systematic review. *J Am Acad Dermatol* 2013;**69**:799–807. doi:10.1016/j.jaad.2013.06.027

38 Paul C, Gallini A, Archier E, *et al.* Evidence-based recommendations on topical treatment and phototherapy of psoriasis: systematic review and expert opinion of a panel of dermatologists. *J Eur Acad Dermatol Venereol* 2012;**26 Suppl 3**:1–10. doi:10.1111/j.1468-3083.2012.04518.x

39 Almutawa F, Alnomair N, Wang Y, *et al.* Systematic review of UV-based therapy for psoriasis. *Am J Clin Dermatol* 2013;**14**:87–109. doi:10.1007/s40257-013-0015-y

40 Dogra S, Mahajan R. Systemic methotrexate therapy for psoriasis: past, present and future. *Clin Exp Dermatol* 2013;**38**:573–88. doi:10.1111/ced.12062

41 Mrowietz U. Cyclosporine as maintenance therapy in patients with severe psoriasis. *J Am Acad Dermatol* 2013;**69**:308–9. doi:10.1016/j.jaad.2012.09.005

42 Sbidian E, Maza A, Montaudie H, *et al.* Efficacy and safety of oral retinoids in different psoriasis subtypes: a systematic literature review. *J Eur Acad Dermatol Venereol* 2011;**25 Suppl 2**:28–33. doi:10.1111/j.1468-3083.2011.03993.x

43 Novelli L, Chimenti MS, Chiricozzi A, *et al.* The new era for the treatment of psoriasis and psoriatic arthritis: perspectives and validated strategies. *Autoimmun Rev* 2014;**13**:64–9. doi:10.1016/j.autrev.2013.08.006

44 Papoutsaki M, Osorio F, Morais P, *et al.* Infliximab in psoriasis and psoriatic arthritis. *BioDrugs* 2013;**27 Suppl 1**:13–23. doi:10.1007/BF03325638

45 Dörner T, Strand V, Castañeda-Hernández G, *et al.* The role of biosimilars in the treatment of rheumatic diseases. *Ann Rheum Dis* 2013;**72**:322–8. doi:10.1136/annrheumdis-2012-202715

46 Schneider CK. Biosimilars in rheumatology: the wind of change. *Ann Rheum Dis* 2013;**72**:315–8. doi:10.1136/annrheumdis-2012-202941

47 Yoo DH, Hrycaj P, Miranda P, *et al.* A randomised, double-blind, parallel-group study to demonstrate equivalence in efficacy and safety of CT-P13 compared with innovator infliximab when coadministered with methotrexate in patients with active rheumatoid arthritis: the PLANETRA study. *Ann Rheum Dis* 2013;**72**:1613–20. doi:10.1136/annrheumdis-2012-203090

48 Park W, Hrycaj P, Jeka S, *et al.* A randomised, double-blind, multicentre, parallel-group, prospective study comparing the pharmacokinetics, safety, and efficacy of CT-P13 and innovator infliximab in patients with ankylosing spondylitis: the PLANETAS study. *Ann Rheum Dis* 2013;**72**:1605–12. doi:10.1136/annrheumdis-2012-203091

49 Prevoo ML, van 't Hof MA, Kuper HH, *et al.* Modified disease activity scores that include twenty-eight-joint counts. Development and validation in a prospective longitudinal study of patients with rheumatoid arthritis. *Arthritis Rheum* 1995;**38**:44–8.

50 Gossec L, Dougados M, Rincheval N, *et al.* Elaboration of the preliminary Rheumatoid Arthritis Impact of Disease (RAID) score: a EULAR initiative. *Ann Rheum Dis* 2009;**68**:1680–5. doi:10.1136/ard.2008.100271

51 Gossec L, Paternotte S, Aanerud GJ, *et al.* Finalisation and validation of the rheumatoid arthritis impact of disease score, a patient-derived composite measure of impact of rheumatoid arthritis: a EULAR initiative. *Ann Rheum Dis* 2011;**70**:935–42. doi:10.1136/ard.2010.142901

52 Gossec L, de Wit M, Kiltz U, *et al.* A patient-derived and patient-reported outcome measure for assessing psoriatic arthritis: elaboration and preliminary validation of the Psoriatic Arthritis Impact of Disease (PsAID) questionnaire, a 13-country EULAR initiative. *Ann Rheum Dis* 2014;**73**:1012–9. doi:10.1136/annrheumdis-2014-205207

53 Garrett S, Jenkinson T, Kennedy LG, *et al.* A new approach to defining disease status in ankylosing spondylitis: the Bath Ankylosing Spondylitis Disease Activity Index. *J Rheumatol* 1994;**21**:2286–91.

54 Machado P, Landewe R, Lie E, *et al.* Ankylosing Spondylitis Disease Activity Score (ASDAS): defining cut-off values for disease activity states and improvement scores. *Ann Rheum Dis* 2011;**70**:47–53. doi:10.1136/ard.2010.138594

55 Schroeder KW, Tremaine WJ, Ilstrup DM. Coated oral 5-aminosalicylic acid therapy for mildly to moderately active ulcerative colitis. A randomized study. *N Engl J Med* 1987;**317**:1625–9. doi:10.1056/NEJM198712243172603

56 Harvey RF, Bradshaw JM. A simple index of Crohn's-disease activity. *Lancet* 1980;**1**:514.

57 Best WR, Becktel JM, Singleton JW, *et al.* Development of a Crohn‘s disease activity index. National Cooperative Crohn’s Disease Study. *Gastroenterology* 1976;**70**:439–44.

58 Fredriksson T, Pettersson U. Severe psoriasis--oral therapy with a new retinoid. *Dermatologica* 1978;**157**:238–44.

59 Fries JF, Spitz P, Kraines RG, *et al.* Measurement of patient outcome in arthritis. *Arthritis Rheum* 1980;**23**:137–45.

60 Pincus T, Summey JA, Soraci SA, *et al.* Assessment of patient satisfaction in activities of daily living using a modified Stanford Health Assessment Questionnaire. *Arthritis Rheum* 1983;**26**:1346–53.

61 Guyatt G, Mitchell A, Irvine EJ, *et al.* A new measure of health status for clinical trials in inflammatory bowel disease. *Gastroenterology* 1989;**96**:804–10.

62 Finlay AY, Khan GK. Dermatology Life Quality Index (DLQI)--a simple practical measure for routine clinical use. *Clin Exp Dermatol* 1994;**19**:210–6.

63 Ware JE, Sherbourne CD. The MOS 36-item short-form health survey (SF-36). I. Conceptual framework and item selection. *Med Care* 1992;**30**:473–83.

64 Brazier J, Roberts J, Deverill M. The estimation of a preference-based measure of health from the SF-36. *J Health Econ* 2002;**21**:271–92.

65 Brooks R. EuroQol: the current state of play. *Health Policy* 1996;**37**:53–72.

66 EuroQol Group. EuroQol--a new facility for the measurement of health-related quality of life. *Health Policy* 1990;**16**:199–208.

67 Reilly MC, Zbrozek AS, Dukes EM. The validity and reproducibility of a work productivity and activity impairment instrument. *Pharmacoeconomics* 1993;**4**:353–65.

68 Chularojanamontri L, Griffiths CEM, Chalmers RJG. The Simplified Psoriasis Index (SPI): a practical tool for assessing psoriasis. *J Invest Dermatol* 2013;**133**:1956–62. doi:10.1038/jid.2013.138

# APPENDICES

## Trial flow chart

|  | **Screening Evaluation** | **Baseline visit** | **Regular visit**^6)^ | **End of study visit** |
| --- | --- | --- | --- | --- |
| Informed consent | X |  |  |  |
| Eligibility assesment | X |  |  |  |
| Demographics |  | X |  |  |
| Medical history |  | X |  |  |
| Physical Examination |  | X |  |  |
| Vital signs |  | X | X | X |
| Laboratory samples^1)^ |  | X | X | X |
| Biobank samples |  | X^2)^ | X^3)^ | X^3)^ |
| Patient reported outcomes^4)^ |  | X | X | X |
| Assessments of disease activity^5)^ |  | X | X | X |
| Adverse event |  | X | X | X |
| Record of concomitant medication |  | X | X | X |
| Treatment administration |  | X | X |  |

1. Hemoglobin, HTC, erythrocytes, white blood cells with differentials, platelet counts, AST and/or ALT, ALP, albumin, creatinine, random glucose, potassium, sodium, urinalysis (blood, protein, glucose), CRP, ESR, calprotectin (IBD)
2. Serum and fullblood
3. Only serum
4. Consisting of:

- Patient Global Assessment of disease activity (NRS)
- EQ-5D
- SF-36
- WPAI-GH
- RA: M-HAQ RAID
- PsA: M-HAQ, PsAID
- SpA: M-HAQ, BASDAI
- UC and CD: IBDQ
- Psoriasis: DLQI

1. Consisting of:

- Nurse/investigator global assessment of disease activity (NRS)
- RA and SpA: DAS28
- SpA: ASDAS
- UC: Partial Mayo score
- CD: HBI
- Psoriasis: PASI

1. Visits carried out according to patient’s pre-randomization treatment shcedule

## RAID questionnaire

## PsAID Questionnaire

## BASDAI questionnaire

## Partial Mayo Score

|  | **Assessment Category** | | |
| --- | --- | --- | --- |
| **Score** | **Stool frequency^1^** | **Rectal bleeding^2^** | **Physician’s global assessment^3^** |
| 0 | Normal number of stools | No blood seen | Normal |
| 1 | One to two stools more than normal | Streaks of blood with stool less than half the time | Mild disease |
| 2 | Three to four stools more than normal | Obvious blood with stool most of the time | Moderate disease |
| 3 | Five or more stools than normal | Blood alone passes | Severe disease |
| Subscore | 0-3 | 0-3 | 0-3 |

1. Each patient serves as his or her own control to establish the degree of abnormality of the stool frequency.
2. The daily bleeding score represents the most severe bleeding of the day.
3. The physician’s global assessment acknowledges the three other criteria, the patient’s daily recollection of abdominal discomfort and general sense of well being, and other observations, such as physical findings and the patient’s performance status.

## Harvey-Bradshaw Index

| 1. General well-being  (yesterday) | - Very well = 0 - Slightly below par = 1 - Poor = 2 - Very poor = 3 - Terrible = 4 |
| --- | --- |
| 2. Abdominal pain  (yesterday) | - None = 0 - Mild = 1 - Moderate = 2 - Severe = 3 |
| 3. Number of liquid or soft stools per day (yesterday) = |  |
| 4. Abdominal mass | - None = 0 - Dubious = 1 - Definite = 2 - Definite and tender = 3 |
| 5. Complications  (Check any that apply; score one per item except for first box) | - None - Arthralgia - Uveitis - Erythema nodosum - Aphthous ulcers - *Pyoderma gangrenosum* - Anal fissure - New fistula - Abcess |

Add scores of questions 1 through 5 to compute the Harvey-Bradshaw Index

## MHAQ

Please check the response that best describes your usual abilities OVER THE COURSE OF THE LAST WEEK

| Are you able to: | Without any difficulty | With some difficulty | With much difficulty | Unable to do |
| --- | --- | --- | --- | --- |
| Dress yourself, including tying shoelaces and doing buttons? | - 0 | - 1 | - 2 | - 3 |
| Get in and out of bed? | - 0 | - 1 | - 2 | - 3 |
| Lift a full cup or glass to your mouth? | - 0 | - 1 | - 2 | - 3 |
| Walk outdoors on flat ground? | - 0 | - 1 | - 2 | - 3 |
| Wash and dry your entire body? | - 0 | - 1 | - 2 | - 3 |
| Bend down to pick up clothing from the floor? | - 0 | - 1 | - 2 | - 3 |
| Turn regular faucets on and off? | - 0 | - 1 | - 2 | - 3 |
| Get in and out of a bus, car, train, or airplane? | - 0 | - 1 | - 2 | - 3 |
|  |  |  |  |  |

## IBDQ


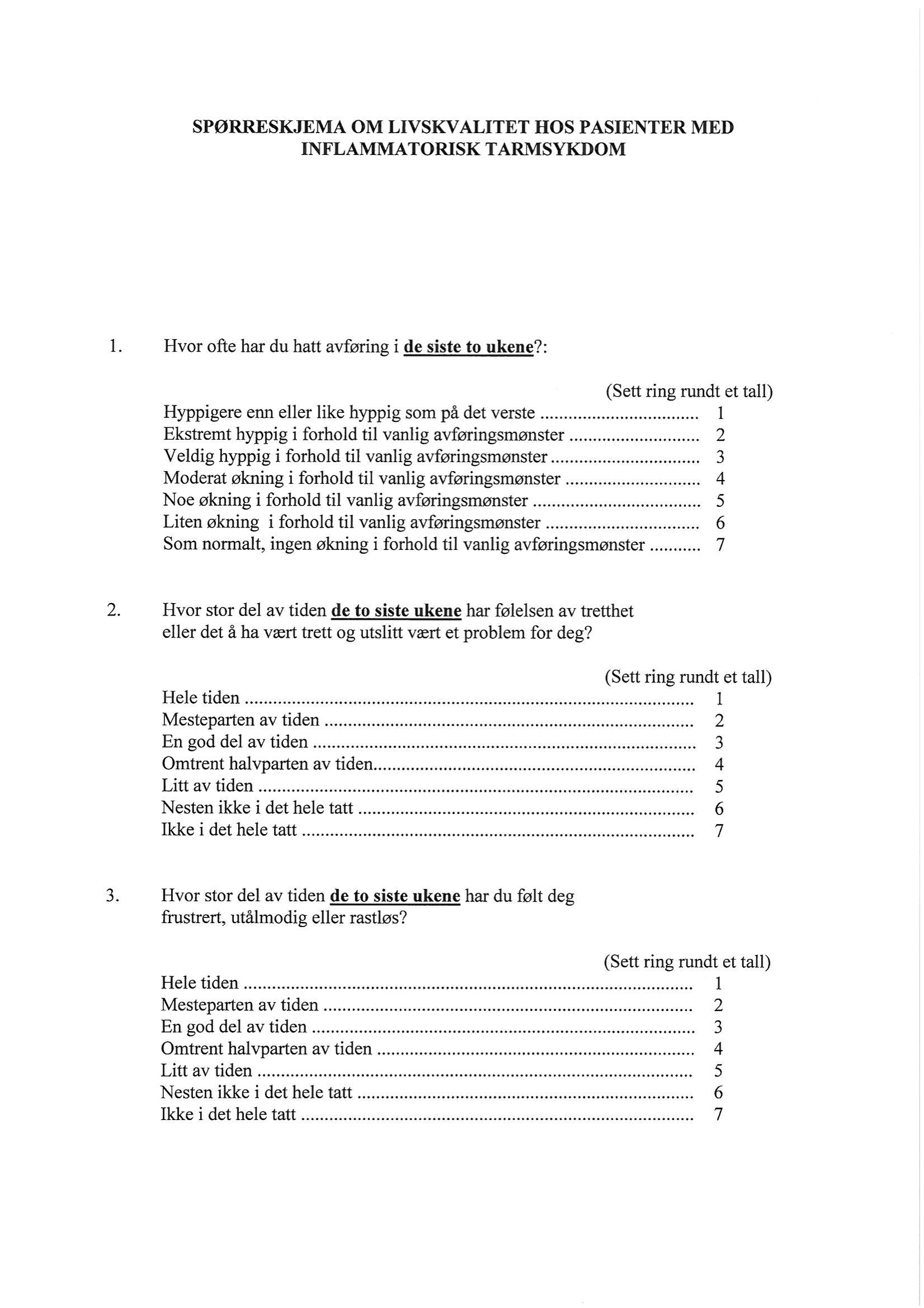


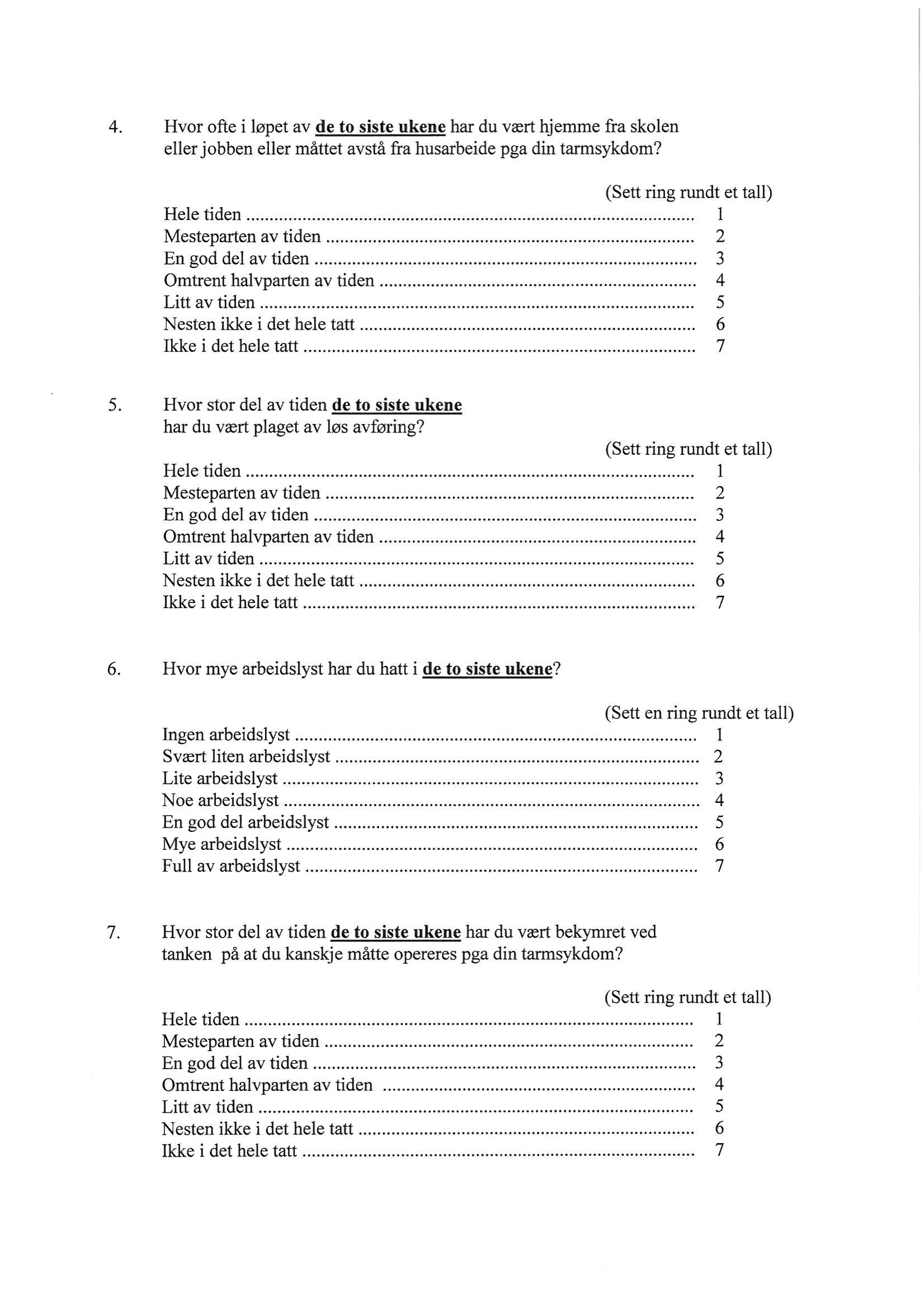


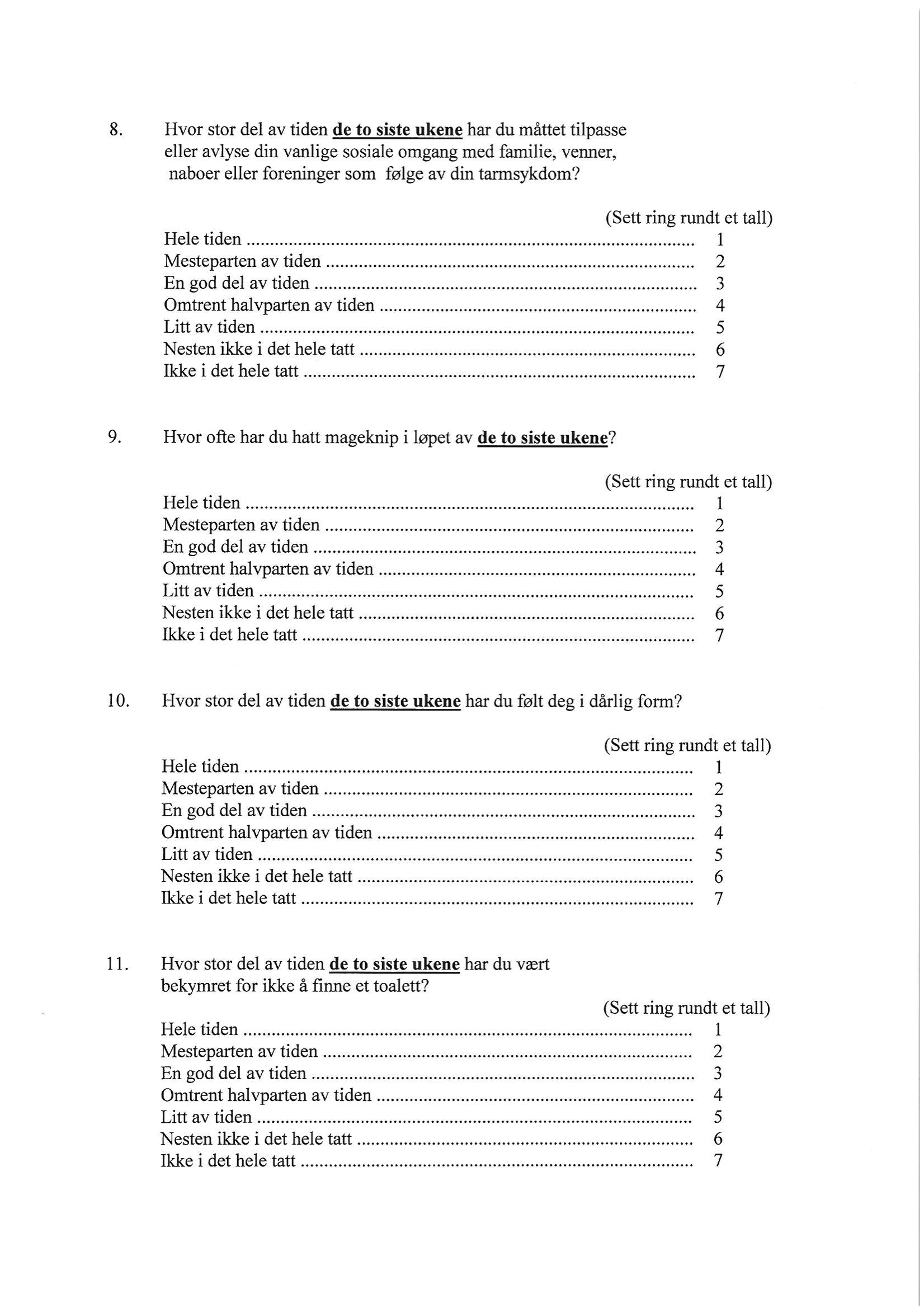


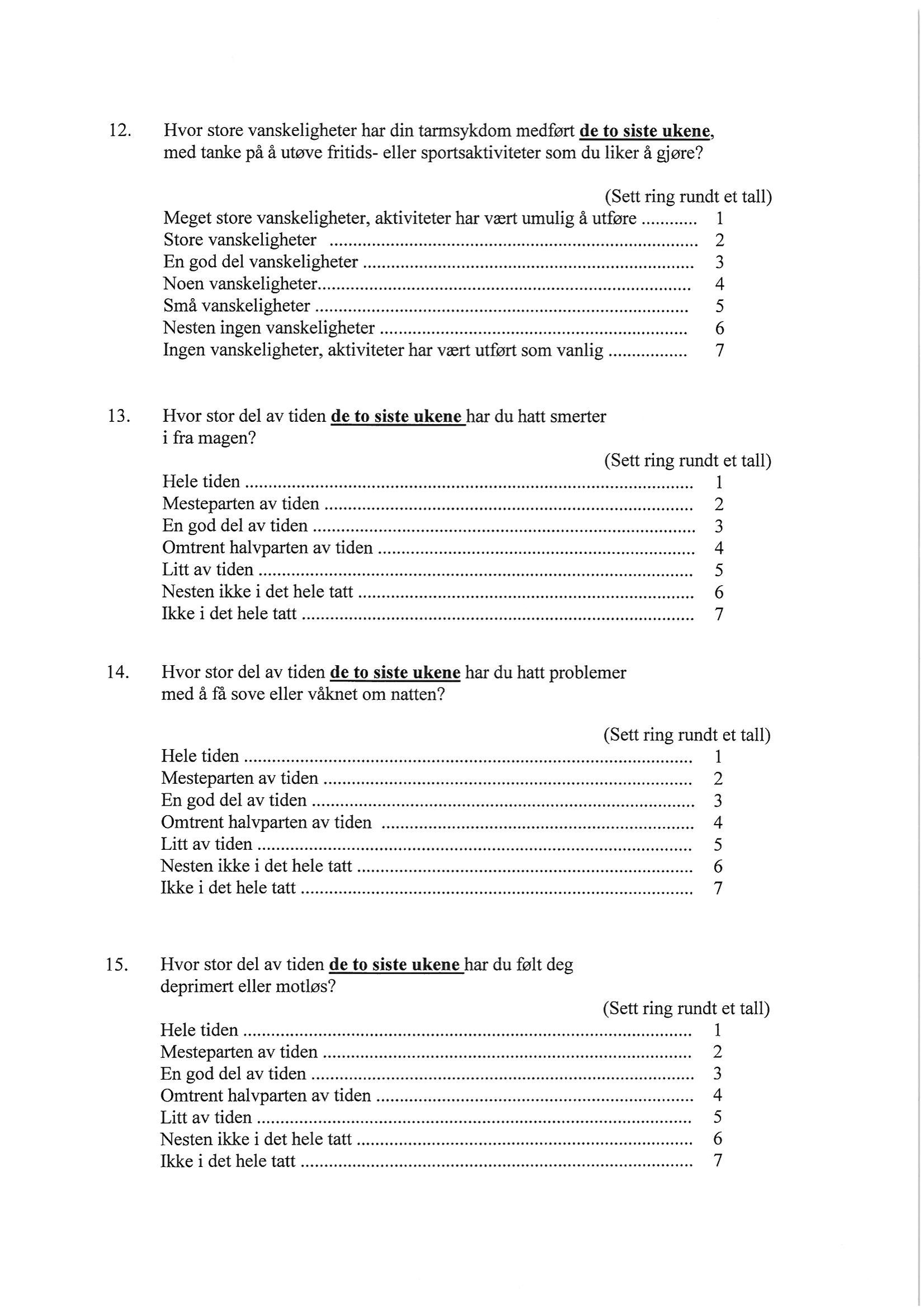


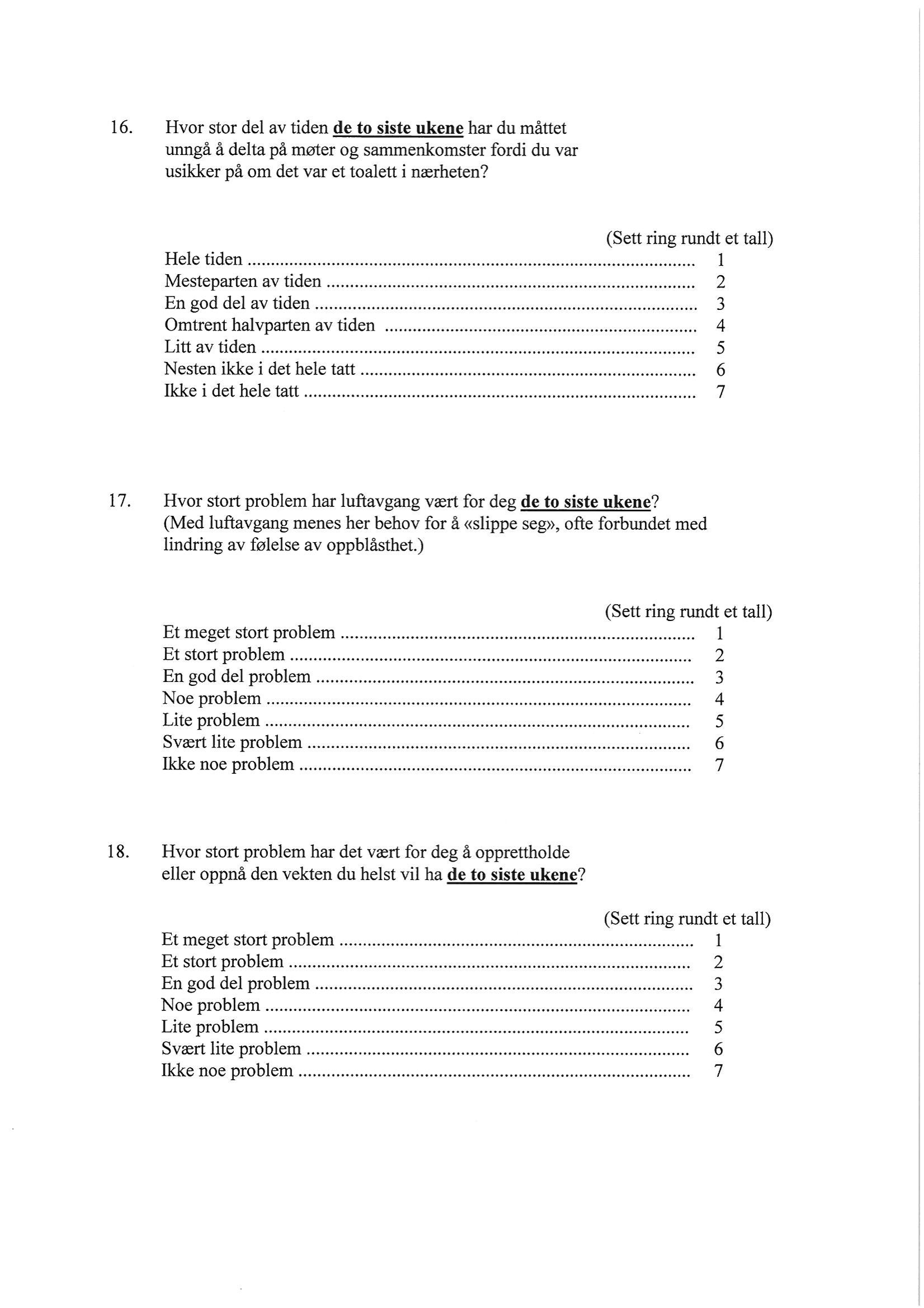


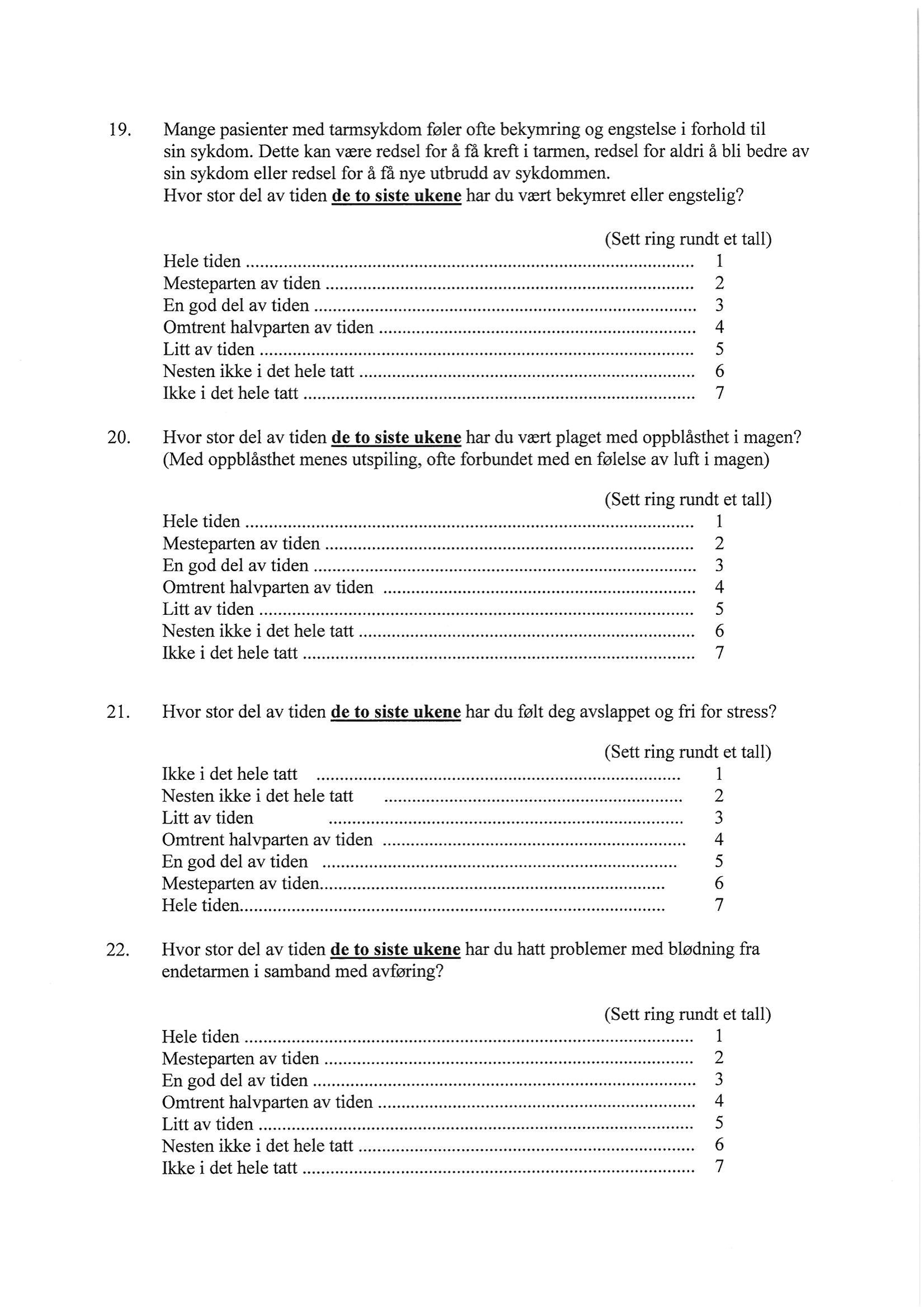


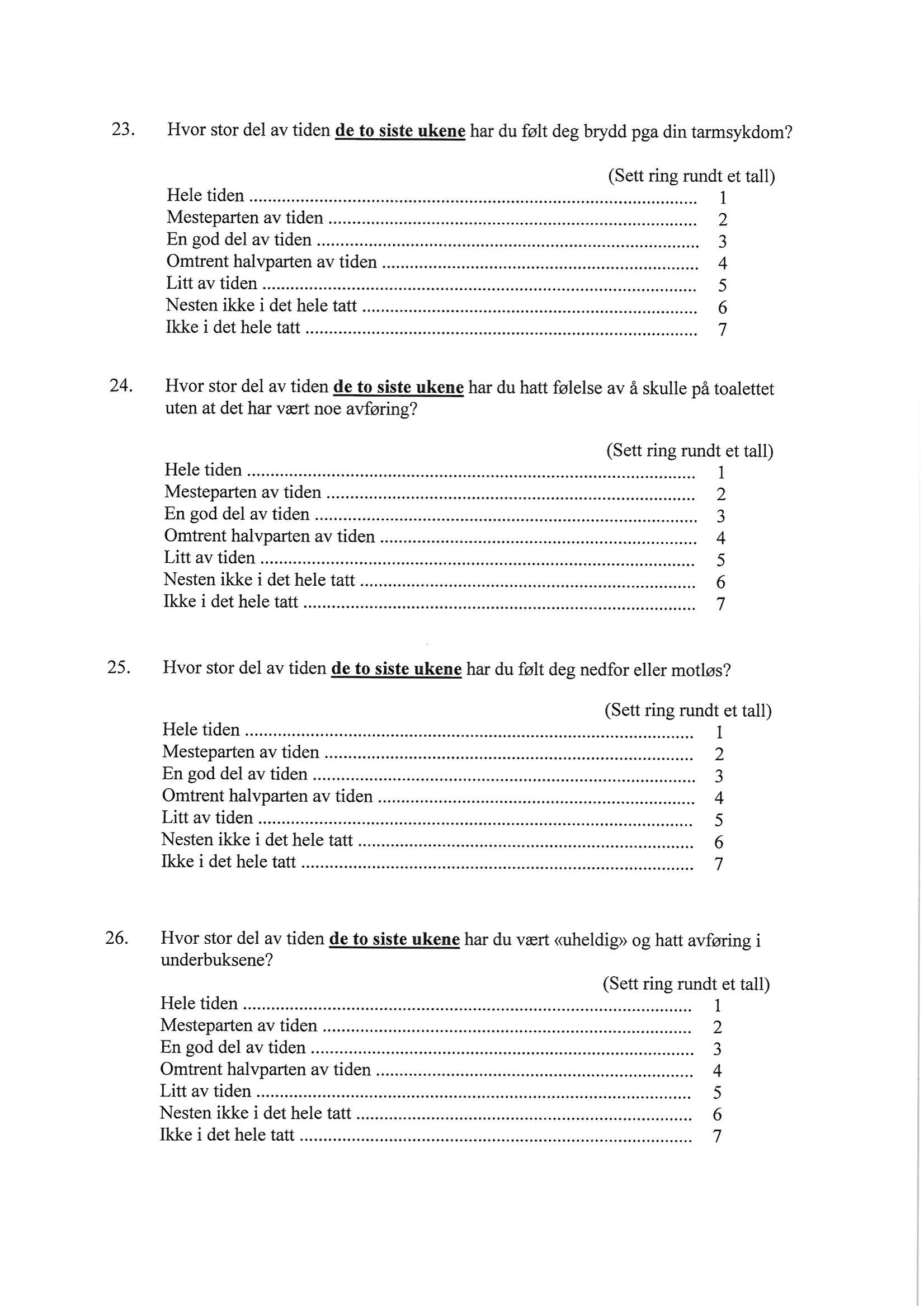


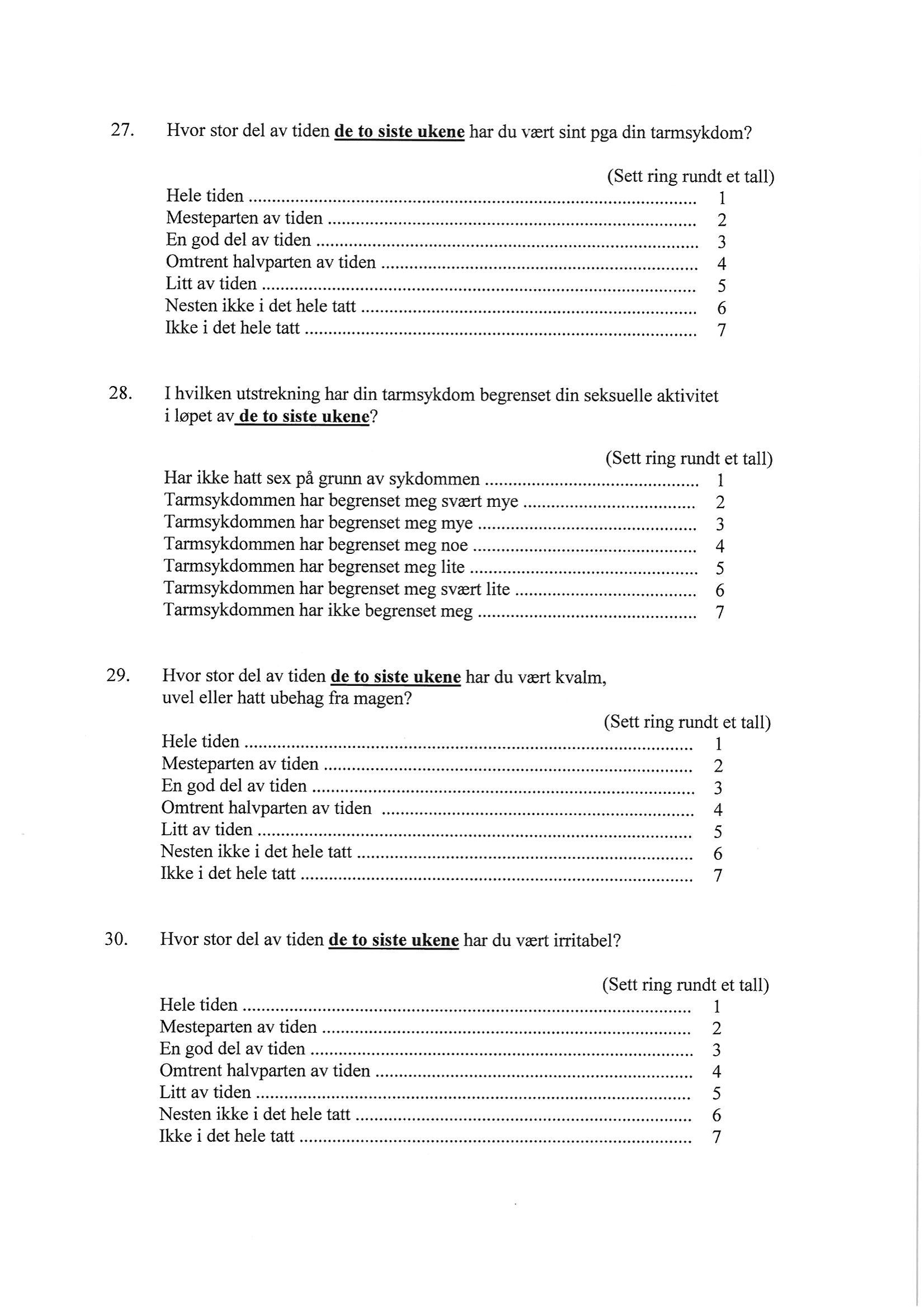


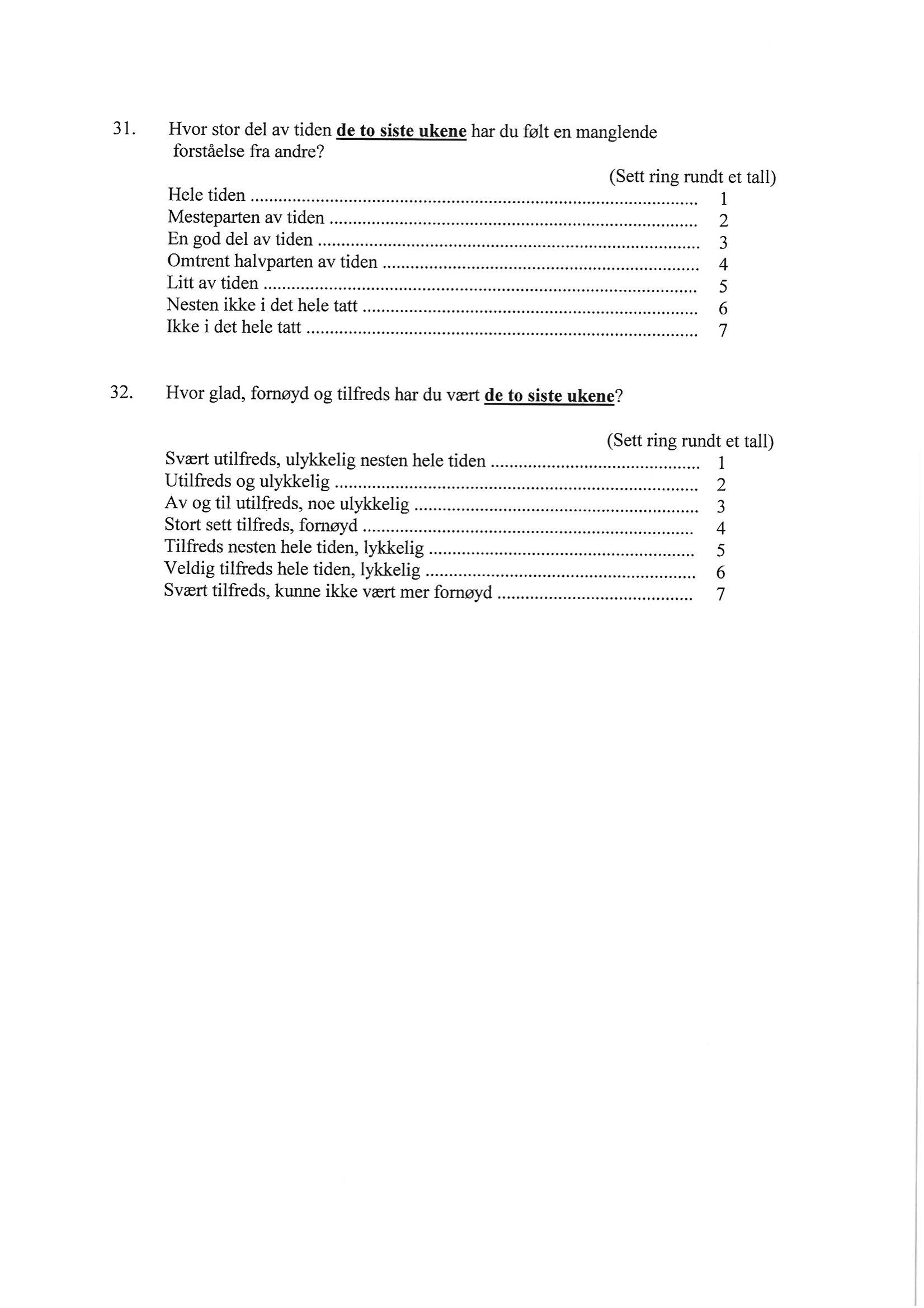


## DLQI

The aim of this questionnaire is to measure how much your skin problem has affected your life OVER THE LAST WEEK. Please tick one box for each question.

1. Over the last week, how itchy, sore, Very much 

painful or stinging has your skin A lot 

been? A little 

Not at all 

2. Over the last week, how embarrassed Very much 

or self conscious have you been because A lot 

of your skin? A little 

Not at all 

3. Over the last week, how much has your Very much 

skin interfered with you going A lot 

shopping or looking after your home or A little 

garden? Not at all  Not relevant 

4. Over the last week, how much has your Very much 

skin influenced the clothes A lot 

you wear? A little 

Not at all  Not relevant 

5. Over the last week, how much has your Very much 

skin affected any social or A lot 

leisure activities? A little 

Not at all  Not relevant 

6. Over the last week, how much has your Very much 

skin made it difficult for A lot 

you to do any sport? A little 

Not at all  Not relevant 

7. Over the last week, has your skin prevented Yes 

you from working or studying? No  Not relevant 

If "No", over the last week how much has A lot 

your skin been a problem at A little 

work or studying? Not at all 

8. Over the last week, how much has your Very much 

skin created problems with your A lot 

partner or any of your close friends A little 

or relatives? Not at all  Not relevant 

9. Over the last week, how much has your Very much 

skin caused any sexual A lot 

difficulties? A little 

Not at all  Not relevant 

10. Over the last week, how much of a Very much 

problem has the treatment for your A lot 

skin been, for example by making A little 

your home messy, or by taking up time? Not at all  Not relevant 

## SF-36


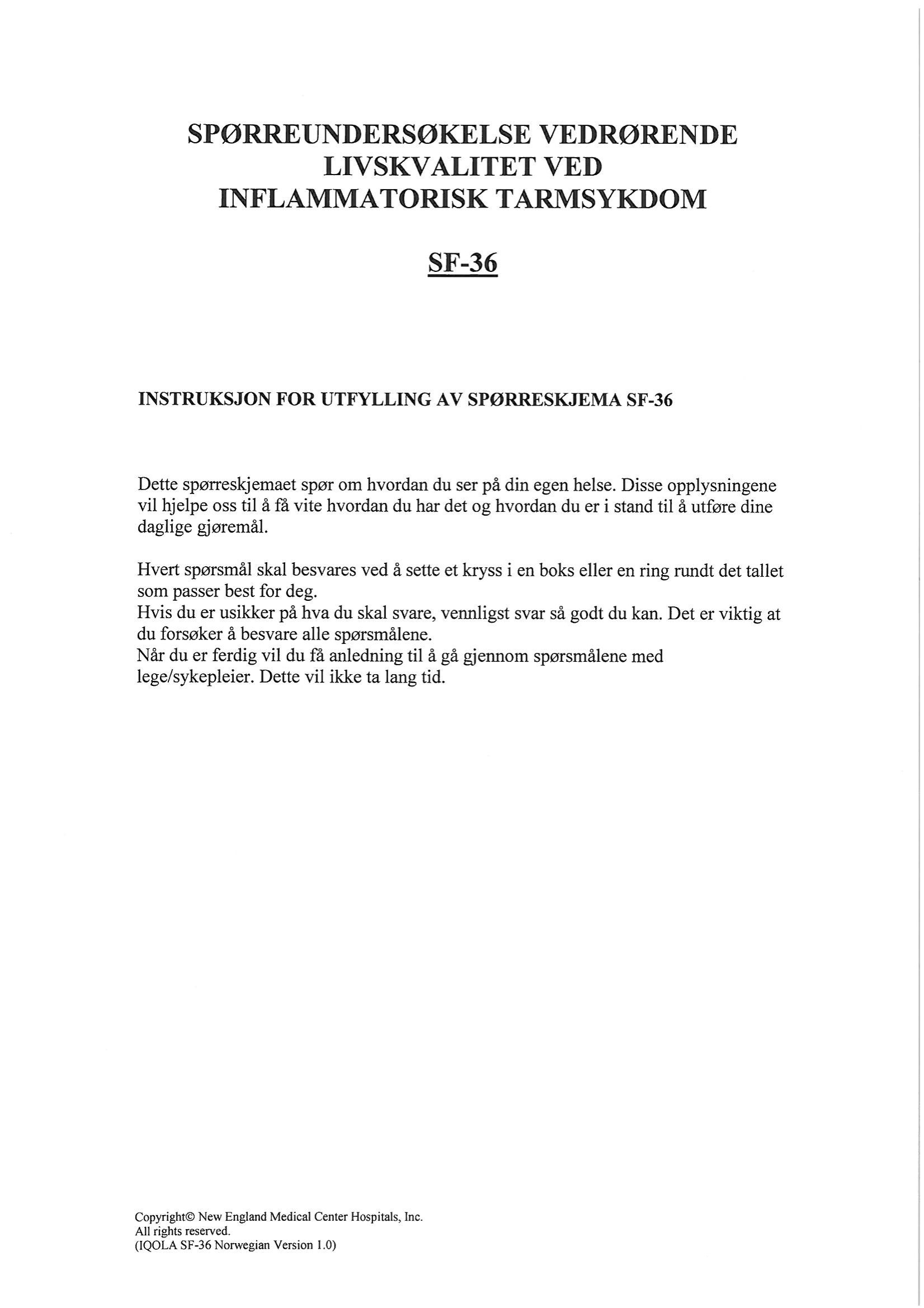


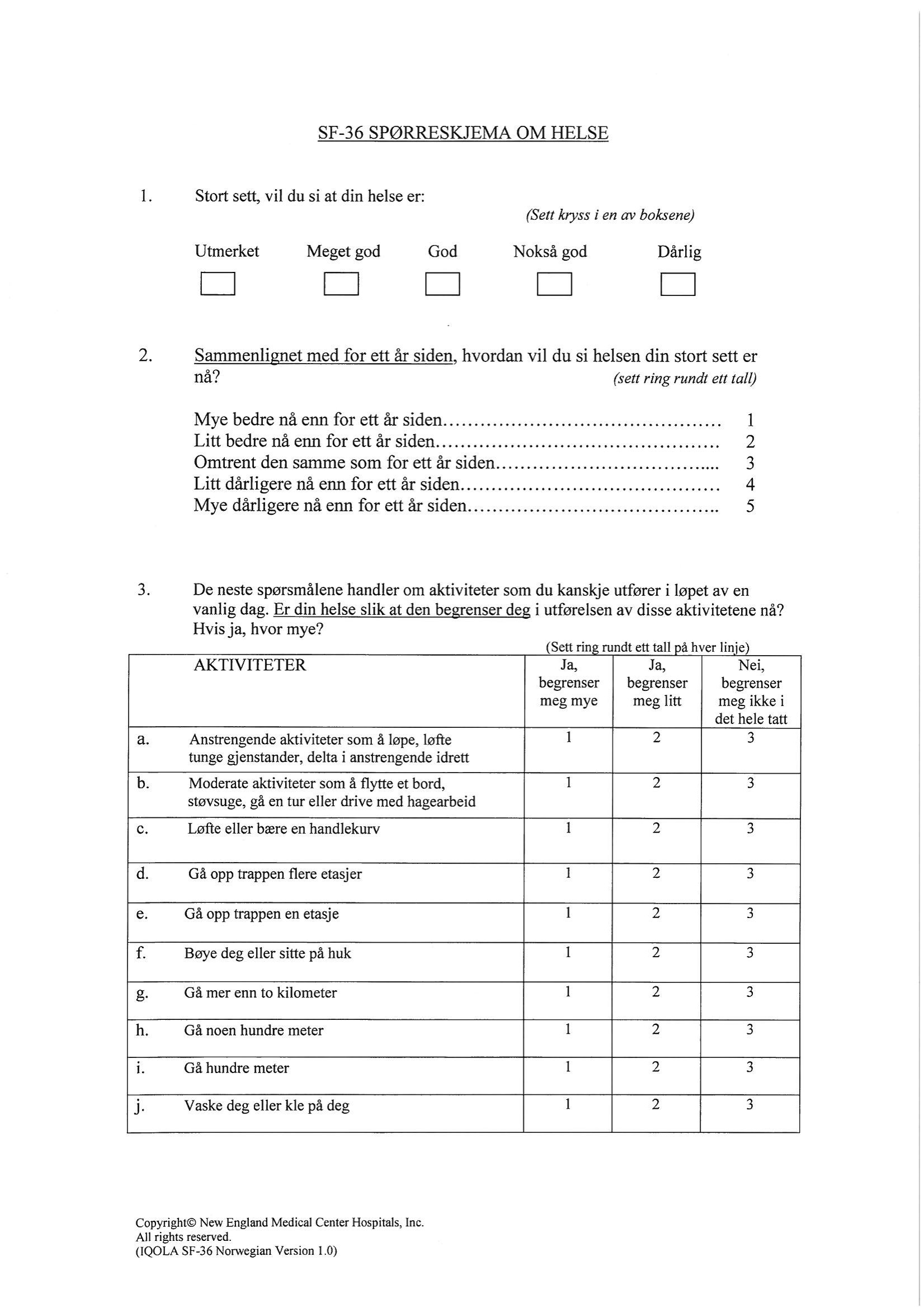


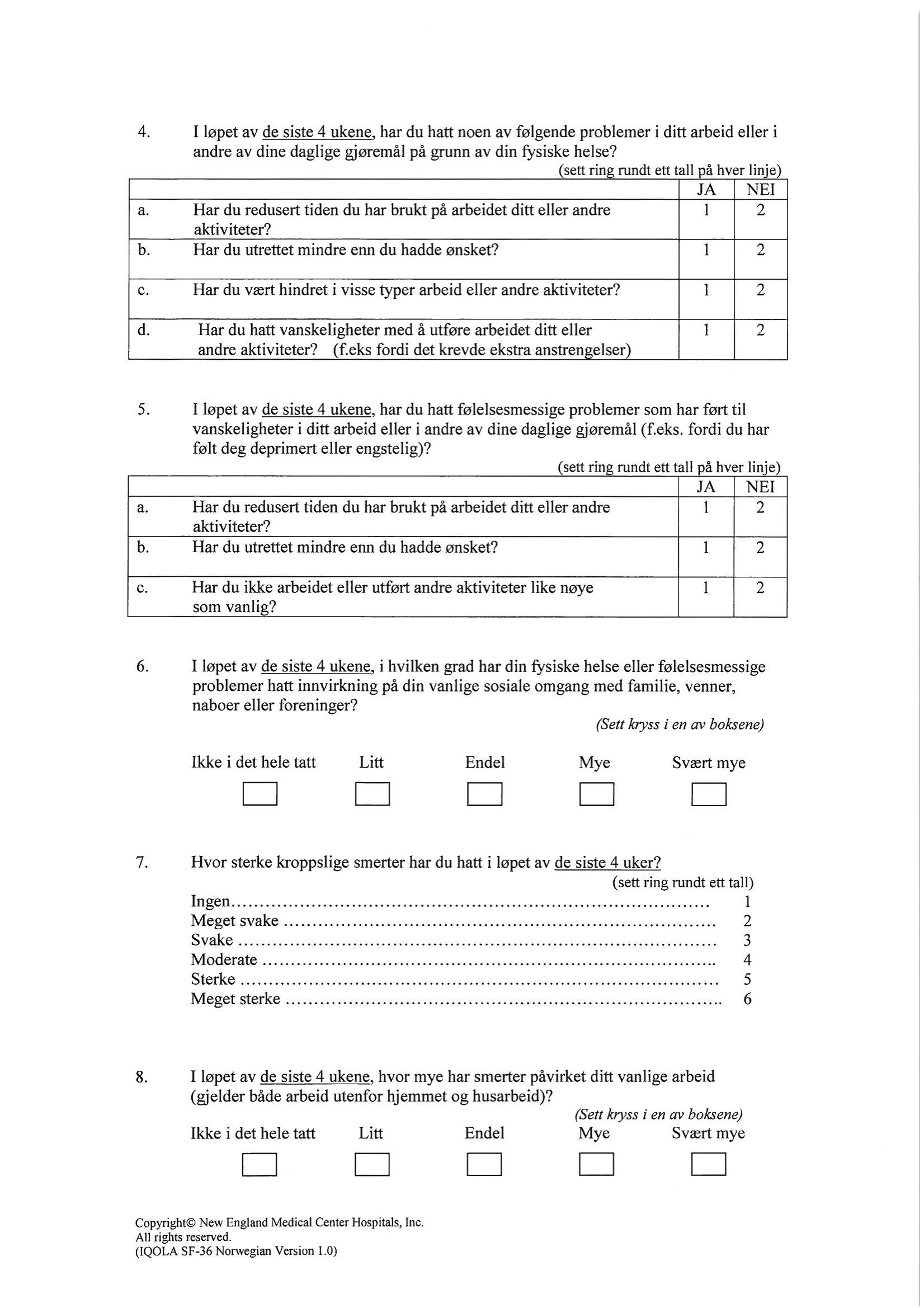


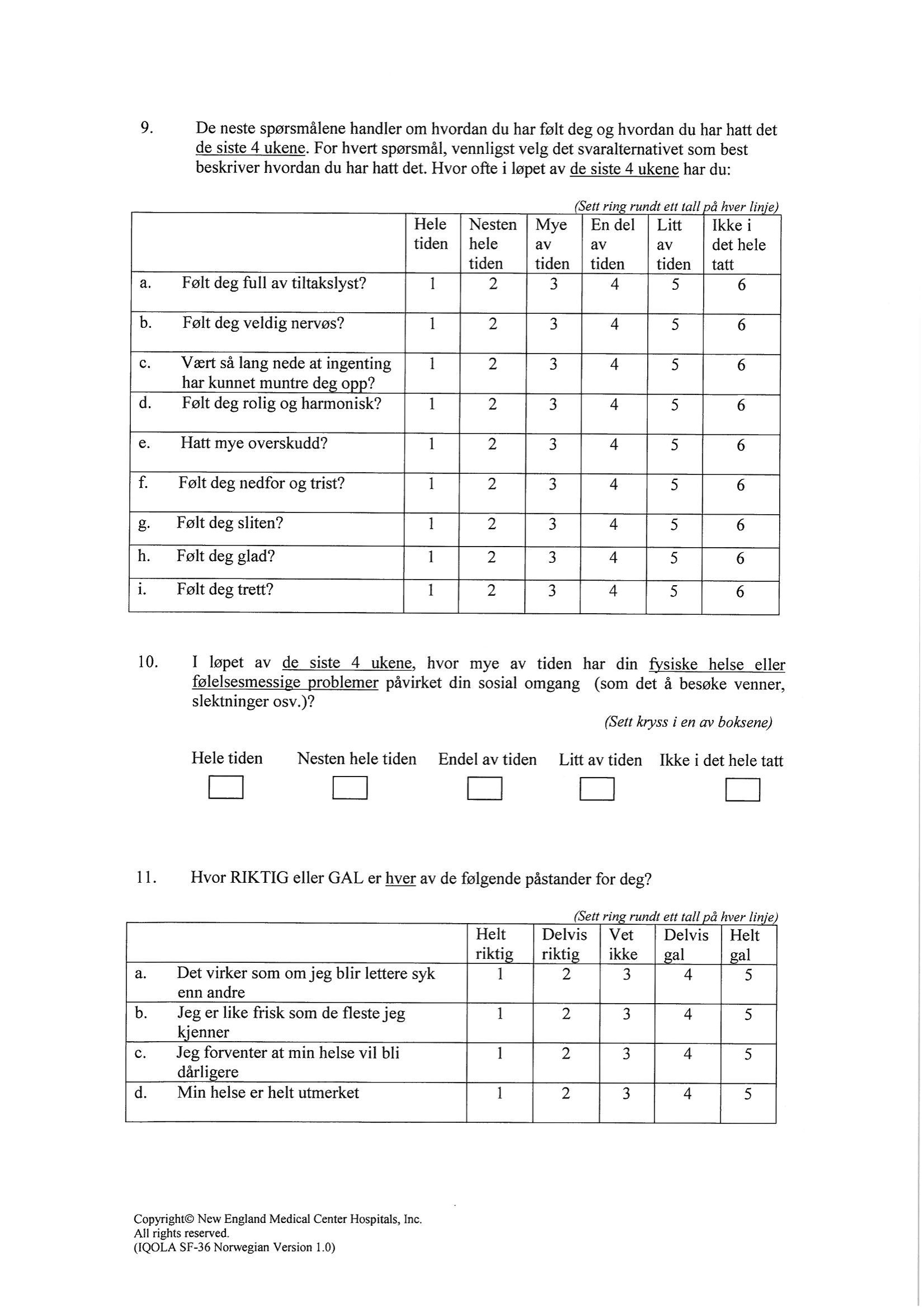


## EQ-5D

|  | **NOEN SPØRSMÅL OM LIVSKVALITET**  **EQ-5D** |  |
| --- | --- | --- |
|  | Vis hvilke utsagn som passer best på din helsetilstand i dag ved å sette et kryss i en av rutene utenfor hver av gruppene nedenfor. |  |
|  | **Gange**  Jeg har ingen problemer med å gå omkring. _1_  Jeg har litt problemer med å gå omkring. _2_  Jeg er sengeliggende. _3_ |  |
|  | **Personlig stell**  Jeg har ingen problemer med personlig stell. _1_  Jeg har litt problemer med å vaske meg eller kle meg. _2_  Jeg er ute av stand til å vaske meg eller kle meg. _3_ |  |
|  | **Vanlige gjøremål** *(for eksempel arbeid, studier, husarbeid, familie- eller fritidsaktiviteter)*  Jeg har ingen problemer med å utføre mine vanlige gjøremål. 1  Jeg har litt problemer med å utføre mine vanlige gjøremål. 2  Jeg er ute av stand til å utføre mine vanlige gjøremål. 3 |  |
|  | **Smerte/ubehag**  Jeg har verken smerte eller ubehag. 1  Jeg har moderat smerte eller ubehag. 2  Jeg har sterk smerte eller ubehag. 3 |  |
|  | **Angst/depresjon**  Jeg er verken engstelig eller deprimert. _1_  Jeg er noe engstelig eller deprimert. _2_  Jeg er svært engstelig eller deprimert. _3_ |  |

## WPAI:GH

**Work Productivity and Activity Impairment Questionnaire:**

**General Health V2.0 (WPAI:GH)**

The following questions ask about the effect of your health problems on your ability to work and perform regular activities. By health problems we mean any physical or emotional problem or symptom. *Please fill in the blanks or circle a number, as indicated.*

1. Are you currently employed (working for pay)? ____ NO ____ YES

*If NO, check “NO” and skip to question 6.*

The next questions are about the **past seven days**, not including today.

2. During the past seven days, how many hours did you miss from work because of your health problems? *Include hours you missed on sick days, times you went in late, left early, etc., because of your health problems. Do not include time you missed to participate in this study.*

*_____*HOURS

3. During the past seven days, how many hours did you miss from work because of any other reason, such as vacation, holidays, time off to participate in this study?

_____HOURS

4. During the past seven days, how many hours did you actually work?

_____HOURS *(If “0”, skip to question 6.)*

5. During the past seven days, how much did your health problems affect your productivity while you were working?

*Think about days you were limited in the amount or kind of work you could do, days you accomplished less than you would like, or days you could not do your work as carefully as usual. If health problems affected your work only a little, choose a low number. Choose a high number if health problems affected your work a great deal.*

Consider only how much health problems affected
productivity while you were working.

| Health problems had no effect on my work |  |  |  |  |  |  |  |  |  |  |  | Health problems completely prevented me from working |
| --- | --- | --- | --- | --- | --- | --- | --- | --- | --- | --- | --- | --- |
|  | 0 | 1 | 2 | 3 | 4 | 5 | 6 | 7 | 8 | 9 | 10 |  |

CIRCLE A NUMBER

6. During the past seven days, how much did your health problems affect your ability to do your regular daily activities, other than work at a job?

*By regular activities, we mean the usual activities you do, such as work around the house, shopping, childcare, exercising, studying, etc. Think about times you were limited in the amount or kind of activities you could do and times you accomplished less than you would like. If health problems affected your activities only a little, choose a low number. Choose a high number if health problems affected your activities a great deal.*

Consider only how much health problems affected your ability
to do your regular daily activities, other than work at a job.

| Health problems had no effect on my daily activities |  |  |  |  |  |  |  |  |  |  |  | Health problems completely prevented me from doing my daily activities |
| --- | --- | --- | --- | --- | --- | --- | --- | --- | --- | --- | --- | --- |
|  | 0 | 1 | 2 | 3 | 4 | 5 | 6 | 7 | 8 | 9 | 10 |  |

CIRCLE A NUMBER
